# Background Filtering of Clinical Metagenomic Sequencing with a Library Concentration-Normalized Model

**DOI:** 10.1128/spectrum.01779-22

**Published:** 2022-09-22

**Authors:** Juan Du, Jingjia Zhang, Dong Zhang, Yiwen Zhou, Pengfei Wu, Wenchao Ding, Jun Wang, Chuan Ouyang, Qiwen Yang

**Affiliations:** a Department of Clinical Laboratory, State Key Laboratory of Complex Severe and Rare Diseases, Peking Union Medical College Hospital, Peking Union Medical College, Chinese Academy of Medical Sciences, Beijing, China; b Hangzhou Matridx Biotechnology Co., Ltd., Hangzhou, Zhejiang, China; Houston Methodist Hospital

**Keywords:** metagenomic sequencing, background filtering, premodeling, linear regression, clinical settings

## Abstract

Metagenomic next-generation sequencing (mNGS) can accurately detect pathogens in clinical samples. However, wet-lab contamination constrains mNGS analysis and may result in erroneous interpretation of results. Many existing methods rely on large-scale observational microbiome studies and may not be applicable to clinical mNGS tests. By generation of a pretrained profile of common laboratory contaminants, we developed an mNGS noise-filtering model based on the inverse linear relationship between microbial sequencing reads and sample library concentration, named the background elimination and correction by library concentration-normalized (BECLEAN) model. Its efficacy was evaluated with bacteria- and yeast-spiked samples and 28 cerebrospinal fluid (CSF) specimens. The diagnostic accuracy, precision, sensitivity, and specificity of BECLEAN with reference to conventional methods and diagnosis were 92.9%, 86.7%, 100%, and 86.7%, respectively. BECLEAN led to a dramatic reduction of background noise without affecting the true-positive rate and thus can provide a time-saving and convenient tool in various clinical settings.

**IMPORTANCE** Most of the existing methods to remove wet-lab contamination rely on large-scale observational microbiome studies and may not be applicable to clinical mNGS testing in individual cases. In clinical settings, only a handful of samples might be sequenced in a run. The lab-specific microbiome can complicate existing statistical approaches for removing contamination from small-scale clinical metagenomic sequencing data sets; thus, use of a preliminary lab-specific training set is necessary. Our study provides a rapid and accurate background-filtering tool for clinical metagenomic sequencing by generation of a pretrained profile of common laboratory contaminants. Notably, our work demonstrates that the inverse linear relationship between microbial sequencing reads and library concentration can serve to identify true contaminants and evaluate the relative abundance of a taxon in samples by comparing the observed microbial reads to the model-predicted value. Our findings extend the previously published research and demonstrate confirmatory results in clinical settings.

## INTRODUCTION

Metagenomic next-generation sequencing (mNGS) has rapidly emerged as a promising diagnostic tools for infectious diseases in various clinical settings. It can generate both species-level taxonomic resolution and functional genomic information without *a priori* knowledge of the potential pathogens. However, its high sensitivity can also be a drawback that can undermine its application potential, because it easily detects ubiquitous contaminating DNAs. The contaminants may distort taxonomic distributions and relative frequencies in microbial data sets, which may lead to erroneous interpretations and identifications, especially during the analysis of samples with low microbial biomass (1–5). Contamination arising from homologous, similar, or host sequences in bioinformatics analysis is one of the common problems in mNGS analysis, and several tools have been developed to tackle it. Tennessen et al. (6), Lu et al. (7), and Parrello et al. (8) described methods for identifying or removing contaminants from genomes, whereas Burnham et al. developed a low-biomass background correction tool to remove the noise, which was informed by the uniformity of the coverage of microbial genomes and the batch variation (1). Moreover, DeconSeq (9), CS-SCORE (10), and GenCoF (11) were developed to remove human DNA contamination from metagenomic data sets. When it comes to intraspecies and cross-species contamination, several tools, including CroCo (12), ConFindr (13), and Recentrifuge (14), are viable.

On the other hand, contamination can emanate from the reagents, consumables, environment, technicians, or equipment at any point during sample collection, nucleic acid extraction, or library preparation (15–17). The established best practices for mitigating wet-lab contamination constraints focus on inclusion of appropriate laboratory controls during sampling and processing (1, 2). One way of minimizing background noise for certain contaminants is by eliminating sequences that do not reach certain arbitrary thresholds, based on negative-control (NC) or blank-control read counts (18). However, this approach relies on the precondition that the input biomass of negative controls be kept on the same (or similar) level as test samples, because the frequency of contaminating DNA is inversely proportional to the total nucleic acid amount and the prevalence of contaminants will be higher in negative controls than in true samples, due to the absence of competing DNA in the sequencing process (19). Thus, use of negative controls or blank controls to calculate an arbitrary threshold for background filtering is vulnerable, particularly when samples of diverse origins or different types are sequenced together in the absence of appropriate corresponding negative controls. Considering that the biomass and microbiome of controls and samples vary greatly in most cases, it is wiser to use a set of dynamic threshold values to remove contaminants, rather than one fixed set of threshold values.

In the December 2018 issue of *Microbiome*, Davis et al. provided a user-friendly R package entitled decontam and validated their approach on multiple data sets to demonstrate robust detection of contaminating sequences in metagenomic sequencing results (19). The decontam program implements two core methods to identify external contamination: (i) the frequency-based model and (ii) the prevalence-based model. Analyzing sequences with decontam eliminates the need to assign an arbitrary threshold for background filtering and reduces reliance on an *a priori* set list of known contaminants. In April 2019, Zinter et al. (5) presented an amendment to the method of Davis et al. and provided an ingenious approach for quantifying contaminating nucleic acid by associating sequencing read output with the mass of a spike-in control. Their approach relies on incorporation of multiple spike-in DNA or RNA controls into the sample nucleic acid, with the approximate amount of original sample calculated based on the following equation: contaminant mass (in picograms) = spike-in mass (in picograms) × (contaminant reads/spike-in reads) (5, 20). However, the approaches developed by Zinter et al. (5) and Davis et al. (19) both depend on metadata generated from collections of large batches of specimens and controls, which might be a time-consuming process. In a clinical diagnostic laboratory, several samples of diverse origins must be sequenced rapidly and perhaps without the flexibility for experimental design that might be used in an optimal setting to statistically identify contaminants from within a sample set; timely and accurate detection of pathogens is critical for clinical diagnosis and the administration of appropriately targeted antibiotics. In these cases, rapid separation of true microbial components from contaminants by bioinformatics analysis may not be adequately served by the existing methods of Davis et al. and Zinter et al., because those approaches both depend on large metadata sets generated from a set of biological samples or controls.

Based on the assumption that generation of a pretrained profile (training set) of common laboratory contaminants may be useful in clinical settings, here we provide a premodeling solution to differentiate between wet-lab contaminant versus truly present microbial taxa. This approach does not rely on incorporation of spike-in controls to calculate biomass, but rather utilizes the inverse linear relationship between microbial sequencing reads and sample library concentration; it is accordingly termed the background elimination and correction by library concentration-normalized (BECLEAN) model. Furthermore, we validated the model by using contaminant spiked-in samples and 28 clinical cerebrospinal fluid (CSF) specimens, and the model was demonstrated to be effective and promising in mNGS background filtering under varied conditions.

## RESULTS

### Premodeling.

**FIG 1:**
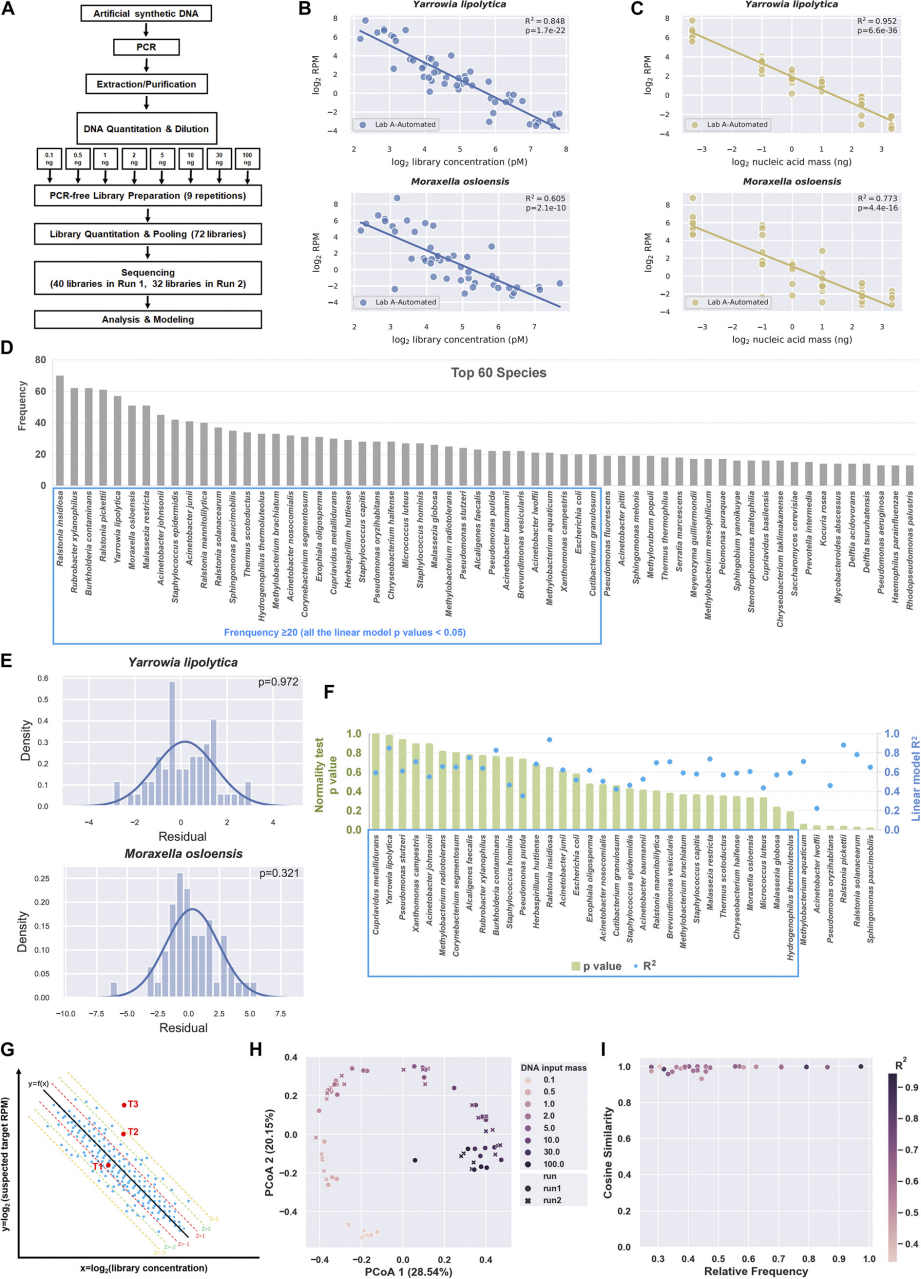
Establishment of BECLEAN and background species profiling. (A) Flowchart of the experimental design for BECLEAN modeling. (B) Contaminant sequencing reads were inversely proportional to library concentration. For a total *n* of 72 in the training data set, sequencing reads for Yarrowia lipolytica and Moraxella osloensis were normalized per million (RPM). The linear regressions associating log_2_-transformed library concentration with log_2_-transformed RPM of Yarrowia lipolytica and Moraxella osloensis are described, with *R*^2^ and *P* values. (C) Contaminant sequencing reads were also inversely proportional to sample input mass. The linear regressions associating log_2_-transformed sample input mass with log_2_-transformed RPM of Yarrowia lipolytica and Moraxella osloensis are described with the *R*^2^ and *P* values. (D) The 60 most frequent contaminants identified in BECLEAN model in lab A. The species with frequencies of occurrence of ≥20 (blue box) were considered the most likely background contaminants, whose *P* values all were <0.05. (E) A histogram of the residual (the difference between each observed value and the model-predicted value) and its probability density distribution for each observation. Residuals approximated a near-normal distribution. (F) The *P* values of the normality test and linear regression *R*^2^ of the 38 most frequent (≥20) contaminants identified in BECLEAN model in lab A. Only 32 candidates passed the test (*P > *0.05) and were kept as background species for subsequent analysis (blue box). (G) Diagram of how BECLEAN filtering works. A *Z*-score can be calculated to describe the deviation from the model-predicted value for a given sample, thus indicating the probability of background contamination or true component in the sample. T1, T2 are probable background contaminants; for T3 there is a high probability that the outlier represents a true component in the sample. (H) PCoA based on Bray-Curtis distances of serially diluted artificial DNA samples sequenced in two runs. The color of data points gradually deepen as DNA input mass increases from 0.1 ng to 100 ng. Dots and crosses represent libraries sequenced in run 1 and run 2, respectively. The microbial profile varied with DNA input mass, but no significant difference in microbial profile was found between the 2 sequencing runs. (I) Similarity of background contaminant models based on two separate runs. All taxa with different relative frequencies showed a high cosine similarity (close to 1.0) between the two models. Each point represents a background species. The *x*-coordinate value indicates the relative frequency of a certain background species in two runs, and the *y*-coordinate value indicates the cosine value of the angle between the two model vectors. Color bar shows the average *R*^2^ of the two models.

To preliminarily generate a profile of common laboratory contaminants and figure out the dynamic threshold values for background filtering, we sought to capture the characteristics of the contaminants from a set of training samples and establish the statistical relationship (premodeling) between contaminant reads and input biomass. We proposed that the quantity of microbial reads for a given taxon in a given sample can be described according to its deviation from the value predicted by the statistical model, and this value can be used to identify whether the taxon is a contaminant or a true microbial component in the sample. Hence, we first performed multiple sequencing by using an artificial DNA fragment which had no sequence similarity with known species (see Table S1 in the supplemental material), to allow for definite alignment after sequencing. The sequenced samples were comprised of different amounts (100 pg to 100 ng, 8 input levels, and 9 duplicates for each group) of the artificial DNA as input template (Fig. 1A), as opposed to environmental or reagent-only samples. In total, we analyzed the data set of 72 libraries (in 2 sequencing runs) prepared from serial dilutions of synthetic DNA in lab A, which rationalized exploration of background contaminants in reagents, consumables, environment, technicians, and equipment. Interestingly, we found the logarithm of read counts per million total reads (RPM) to base 2 to be inversely proportional to log_2_-transformed library concentration for some taxa (e.g., Yarrowia lipolytica and Moraxella osloensis) (Fig. 1B), albeit with a slightly smaller *R*^2^ value compared to that generated by a linear regression model featuring log_2_-transformed sample input mass (Fig. 1C). To identify the most probable contaminating taxa and minimize the effect of random variation, we selected the top 38 species with a frequency of occurrence of ≥20 (*n* = 72) as candidates (Fig. 1D), each of which was described by a linear model (see Fig. S1, left panel). Some of the taxa detected in 29 of these samples had reads outside the linear range (see Materials and Methods) and were therefore excluded from modeling, with an average sequence-to-sample ratio of 0.000013% (0.000008% to 0.000046%; median ratio, 0.000011%). Of the top 38 suspected microbes identified by the modeling process, the residuals of 32 taxa model fits approximated a near-normal distribution (e.g., Yarrowia lipolytica and Moraxella osloensis) (Fig. 1E) and were thought to be significant background contaminants (*P* > 0.05) (Fig. 1F and Fig. S2). The approximately normal distribution of the residuals indicated that the deviation of detected microbial reads from the model-predicted value could serve to identify outliers to the linear relationship while also accounting for varying statistical power at different points along the linear regression. To exclude nucleic acid contamination originating from aerosolized PCR amplicons, we also investigated the genome distribution of reads for each taxon (see Fig. S3) in the data set. Once the model was established, a *Z*-score of a given taxon in the test sample could be calculated from the sample library concentration based on the model fit from the training data set, thus describing the deviation of read density from the model-predicted value, which could then identify outliers. The high *Z*-score was because the read density for the taxon was above what was predicted from contaminants based on the library concentration using the trained model. Then, we can use the sample library concentration and the *Z*-score metric (*Z*-score = 3, set as the threshold) to predict the RPM scope of a certain contaminant taxon in a given sample and to evaluate the relative abundance of a taxon in samples by comparing the detected RPM to the model-predicted value. A diagram of how BECLEAN identifies outlier among contaminants is provided in Fig. 1G. The red dots T1 and T2 represent most-probable contaminants, whereas T3 represents a high likelihood of finding the suspected target taxon in the sample metagenome.

To evaluate the effect of random variation of our premodeling process, we performed principal coordinates analysis (PCoA) to examine the similarity of the microbial communities in 2 separate premodeling sequencing runs. The scatterplot in Fig. 1H shows the taxonomic compositional profiles of libraries of different input biomass when there was no significant difference between the two sequencing runs (permutational multivariate analysis of variance [PERMANOVA] *P* = 0.924). We also established 2 models using the data sets of run 1 and run 2 and inspected the consistency between these two models. As shown in Fig. 1I, species with different relative frequencies showed a high congruence between the 2 models. These results indicated a good reproducibility and robustness of the BECLEAN premodeling approach under relatively stable conditions.

### Verification using contaminant-spiked samples.

**FIG 2:**
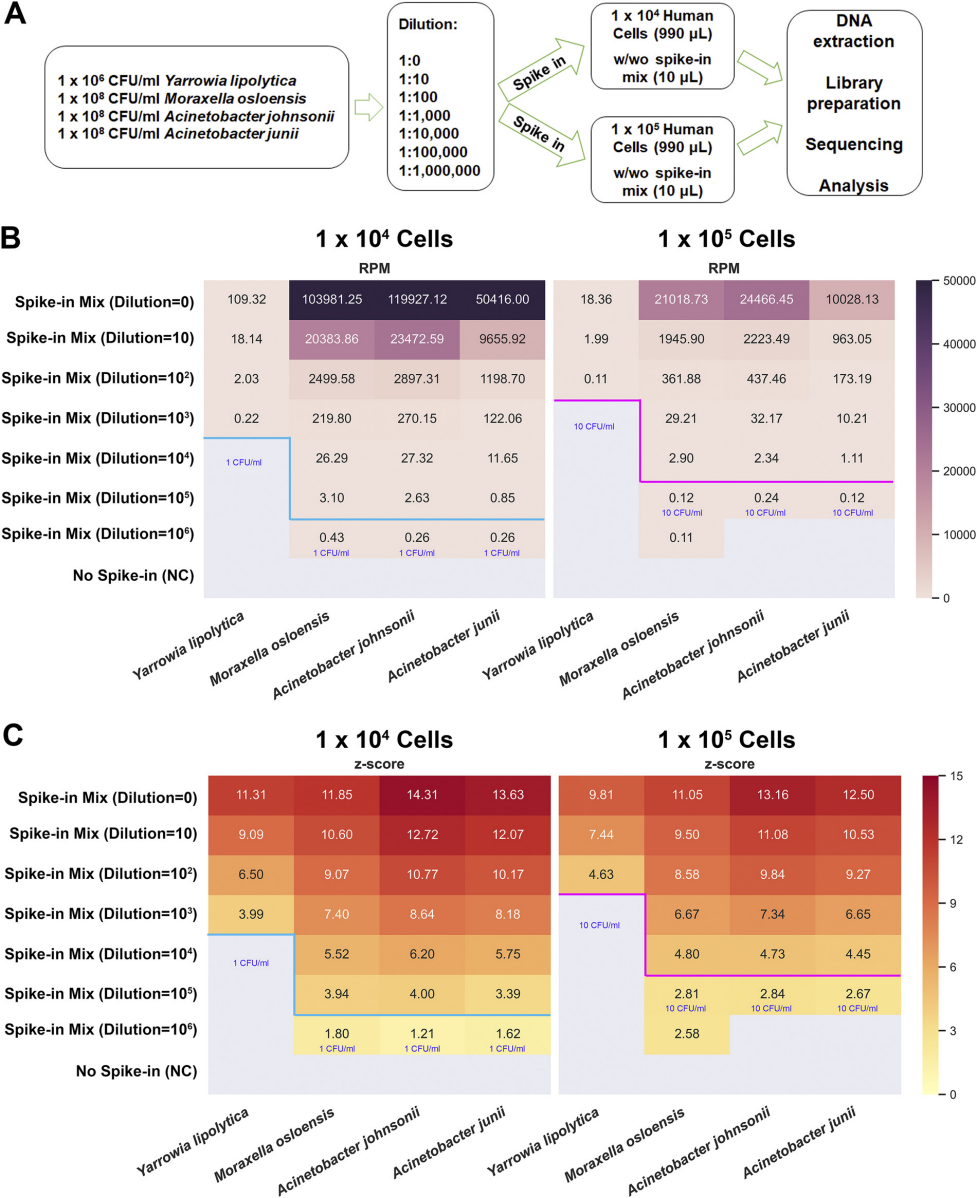
Verification using contaminant-spiked samples. (A) Flowchart of experimental design for verification. (B) Heatmap analysis of the normalized sequencing reads (RPM) of 4 spike-in species (column). Rows are experimental groups with different incorporation amounts of the spike-in mixture. (C) Heatmap analysis of the *Z*-score of 4 spike-in species (column). Rows are experimental groups with different incorporation amounts of spike-in mixture. The blue and magenta lines indicate each identification and filtering limit of BECLEAN for 1 × 10^4^ and 1 × 10^5^ human cells in background, respectively.

To verify the efficacy of BECLEAN filtering and to demonstrate how much biomass is necessary for taxa identified as contaminants in the training set to be accepted as real members of the community in a given sample, we sequenced serial dilutions of samples, with or without a spike-in mix of 4 background contaminants identified in the above-mentioned training data set (including Yarrowia lipolytica, Moraxella osloensis, Acinetobacter johnsonii, and Acinetobacter junii) (Fig. 2A). The BECLEAN pretraining model (Fig. 1) was reused to calculate the *Z*-score (the likelihood of being either contaminants or truly present microbial components) for these 4 spike-in microorganisms. The RPM and BECLEAN *Z*-scores for each taxon are shown in Fig. 2B and C, respectively. For 1 × 10^4^ human cells (mimicking a low-biomass input), BECLEAN removed these 4 microbes to <10 CFU/mL, as their *Z*-scores were less than 3, even though mNGS was able to detect 1 CFU/mL for Moraxella osloensis, Acinetobacter johnsonii, and Acinetobacter junii. Meanwhile, for 1 × 10^5^ human cells (mimicking median biomass input), the limit of detection (LOD) of mNGS was about 100 CFU/mL for Yarrowia lipolytica, 1 CFU/mL for Moraxella osloensis, and 10 CFU/mL for Acinetobacter johnsonii and Acinetobacter junii, but BECLEAN was able to filter these 4 microbes to <100 CFU/mL. These results indicate that BECLEAN may be able to identify those manually spiked-in background species of median or high abundance as true positives, whereas BECLEAN may fail to distinguish between low-abundant spiked-in microbes and endogenous contaminants.

### Validation using CSF samples.

**FIG 3:**
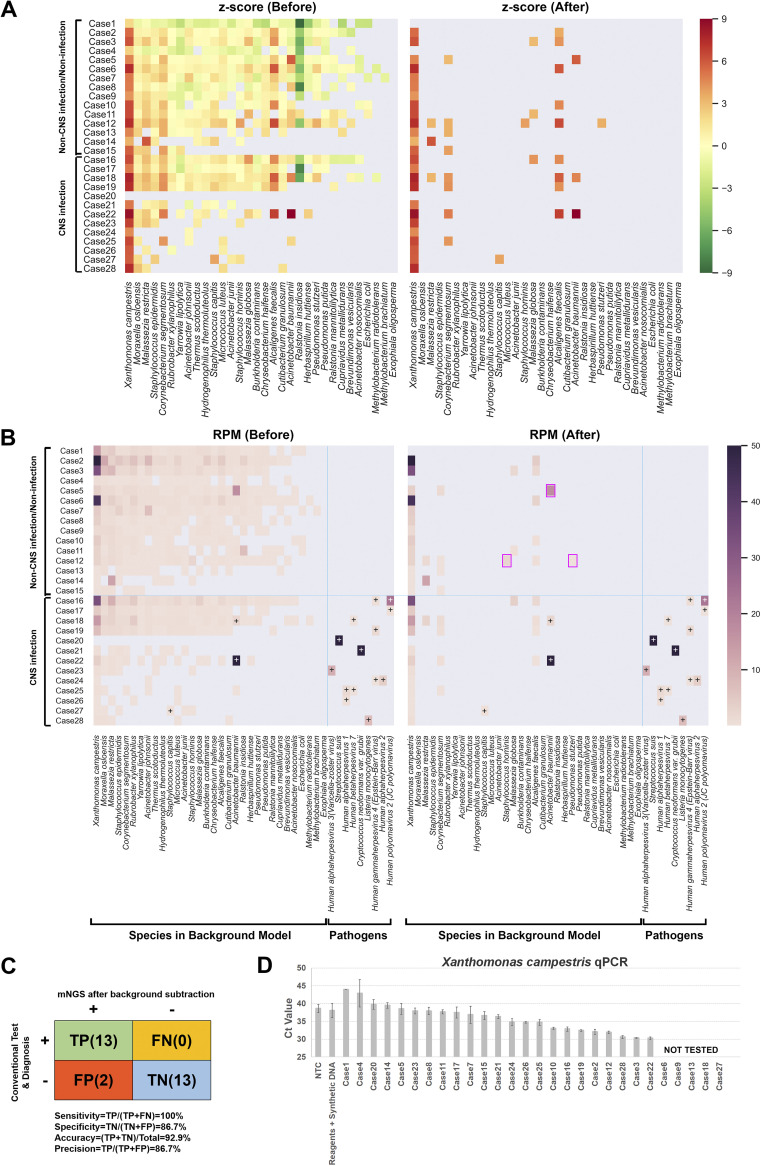
Clinical validation of BECLEAN by using CSF samples. (A) Heatmap analysis of the *Z*-score of 32 background contaminants (column) for 28 CSF samples. Rows are individual samples tested. Abundance matrix before (left) and after (right) application of BECLEAN filtering is shown. (B) Heatmap analysis of normalized sequencing reads (RPM) of 32 background contaminants and 9 suspected pathogens (column) for 28 CSF samples. Rows are individual samples tested. Abundance matrix before (left) and after (right) application of BECLEAN filtering is shown. The magenta boxes indicate probable false-positive detection even after BECLEAN filtering. The box with a plus sign indicates pathogens identified by mNGS in agreement with conventional test or clinical diagnosis. (C) Accuracy of mNGS after application of BECLEAN filtering relative to clinical test or diagnosis of 28 CSF samples. A 2 × 2 contingency table shows the results of the clinical study. TP, true positive, the count of samples with exactly right pathogen(s) reported by mNGS after filtering; TN, true negative, count of true-negative samples in which mNGS did not report any pathogens after filtering; FP, false positive, count of negative samples with at least one pathogen reported by mNGS after filtering; FN, false nagative, count of positive samples with at least one positive pathogen not reported by mNGS. (D) qPCR test for Xanthomonas campestris in 23 CSF samples, library preparation reagents, and no-template control (NTC). Five CSF samples were not tested due to the lack of remaining sample and nucleic acid.

CSF is one of the typical types of samples with low microbial biomass and is susceptible to endogenous contaminants and erroneous interpretations during mNGS analysis. We performed a single-blinded, retrospective study to validate the differential diagnosis of mNGS after BECLEAN filtering using 28 CSF samples, without inclusion of appropriate laboratory controls during sampling and processing. We used the same batch of reagents for validation experiments and the aforementioned modeling. *Z*-scores were calculated for each of the 32 contaminants identified in our model (Fig. 3A, left panel), where only those with a *Z*-score of >3 were retained after BECLEAN filtering (Fig. 3A, right panel). After background filtration, indeed only a few bacterial species could be identified as potential pathogens in some CSF samples (Fig. 3B). We noted that Xanthomonas campestris, Malassezia restricta, Malassezia globosa, Corynebacterium segmentosum, Alcaligenes faecalis, Staphylococcus capitis, Staphylococcus hominis, Pseudomonas stutzeri, and Acinetobacter baumannii, either ubiquitous or common colonizing microorganism on the skin, could not be completely removed from some samples after background filtration. This suggests BECLEAN may not eliminate contaminants highly abundant in the environment, as well as true community members in specimens. Accordingly, Xanthomonas campestris, *Malassezia restricta*, *Malassezia globosa*, *Corynebacterium segmentosum*, and Alcaligenes faecalis were indeed considered true-positive components in a given sample but clinically irrelevant. The common contaminant Acinetobacter baumannii, detected in case 22 and case 18, and Staphylococcus capitis, detected in case 27, were considered true positives and clinically relevant (see Table S2). In contrast, Acinetobacter baumannii identified in case 5 and Staphylococcus hominis and Pseudomonas stutzeri identified in case 12 were regarded as false positives because they were potential pathogens but clinically irrelevant. By comparing the results of conventional etiology testing and clinical diagnosis, the accuracy, precision, sensitivity, and specificity of the mNGS approach were calculated to be 92.9%, 86.7%, 100%, and 86.7%, respectively (Fig. 3C and Table S2). More importantly, the generation of BECLEAN pretrained models was practically efficient and time-saving in clinical settings where only a handful of samples (or even a single sample) might be sequenced in a run, whereas small-scale sample sequencing and insufficiently rigorous case-control design was not the typical use case for the R package decontam employed by Davis et al.

Notably, Xanthomonas campestris was identified in most CSF specimens even after BECLEAN filtering (Fig. 3A and B). We speculate that this organism contaminated the samples at collection or pretreatment with saline dilution. Therefore, we performed a quantitative PCR (qPCR) test to confirm the presence of Xanthomonas campestris in these samples. Compared with the no-template control and control group, the cycle threshold values of contaminating endogenous DNA in some CSF samples were indeed lower (Fig. 3D). This implies traces of Xanthomonas campestris nucleic acid constituted those sample DNAs and will be identified as outliers in the linear regression model.

### Comparison between BECLEAN and decontam.

Next, we evaluated the performance of BECLEAN in a clinical diagnostic laboratory by comparing it to another background removal tool, decontam, which relies on similar statistics principles but without the premodeling procedure to identify contamination. Instead, decontam uses a data set consisting of multiple negative-control samples (e.g., reagent-only or blank sampling instrument samples) and test samples to build frequency-based or prevalence-based models. However, in real-world clinical settings, samples of diverse origins must be sequenced rapidly and perhaps without the flexibility for experimental design (including multiple appropriate controls) that might be used in an optimal setting to statistically identify contaminants from within a sample set. To obtain an evaluation data set suitable for both decontam and BECLEAN, we not only sequenced some libraries for BECLEAN premodeling but also collected several negative CSF samples as well as reagent-only blank controls for decontam modeling. In particular, BECLEAN premodeling was first performed through analysis of 80 libraries prepared from the artificial DNA (used in Fig. 1) of different amounts (100 pg to 100 ng, 8 input levels, and 10 duplicates for each group) and sequenced in two separate runs (see Fig. S4A). PCoA showed that there was no significant difference between the identified microbial profiles of the two separate sequencing runs (PERMANOVA *P* = 0.977) (Fig. S4C). All background contaminants had considerable congruence in models established from these two runs (e.g., Moraxella osloensis, Escherichia coli, and Pseudomonas aeruginosa) (see Fig. S4B), and identified contaminants with different relative frequencies showed a high congruence between the 2 models. Besides, the higher the relative frequency of a certain taxon was, the higher the similarity between these models was, both in terms of cosine similarity and *R*^2^ value of the linear regression model (see Fig. S4D and E).

**FIG 4:**
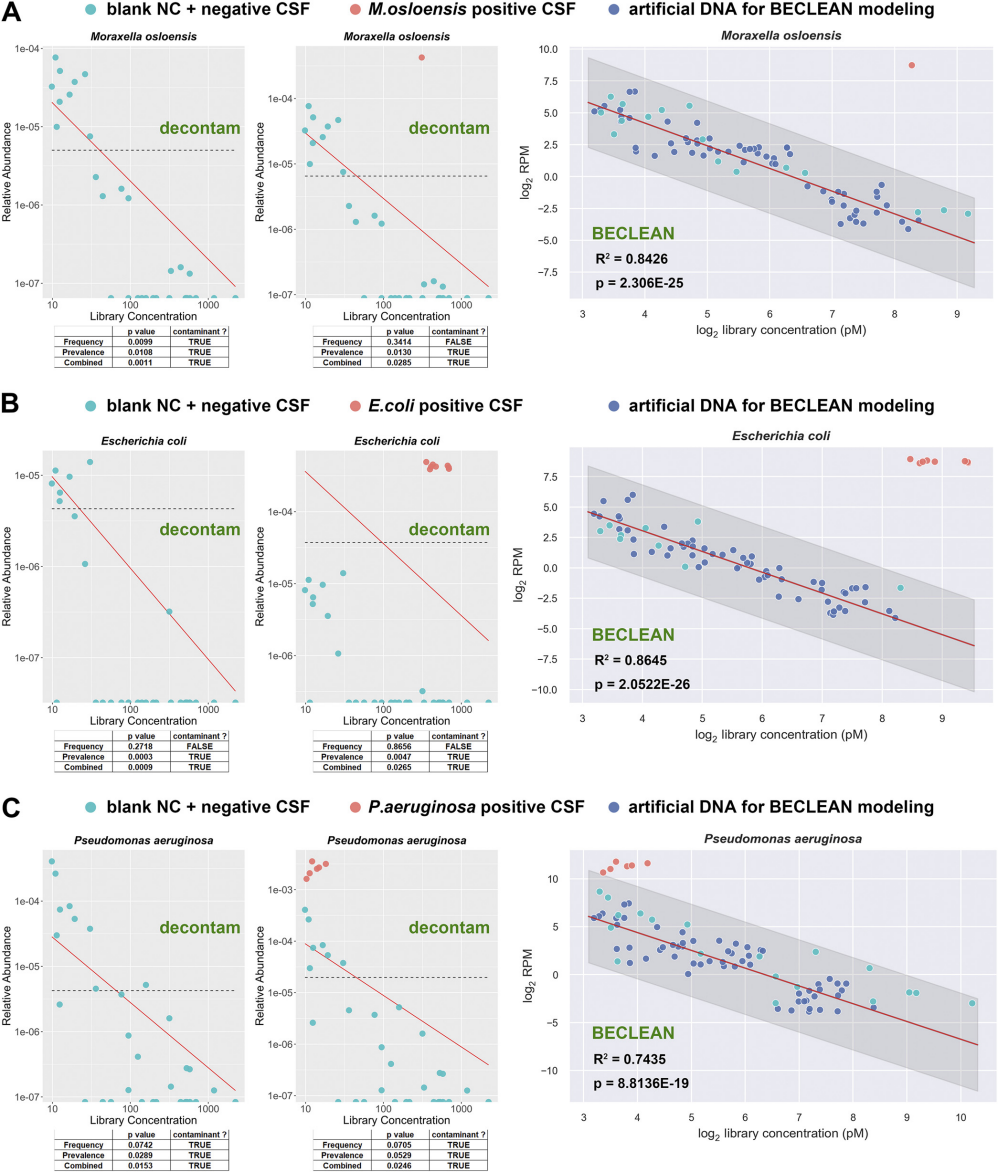
Comparison between BECLEAN and decontam. BECLEAN and decontam modeling and analysis for Moraxella osloensis (A), Escherichia coli (B), and Pseudomonas aeruginosa (C) using different data sets. Left panels, frequency-based distribution plot generated by decontam when analyzing blank NC and negative CSF samples only. Tables below the plots shows the *P* values and conclusions of each method under the default threshold of corresponding data set. Middle panels, frequency-based model after incorporating certain positive sample(s). Tables below the plots shows the *P* values and conclusion of each method under default threshold of corresponding data set. Right panels, preestablished model (red line) and *Z* ± 3 region (gray area) of BECLEAN, and read distribution of negative and positive samples. Cyan dots, blank NC and negative CSF samples; red dots, pathogen-incorporated positive samples; blue dots, artificial DNA samples for BECLEAN modeling.

For decontam modeling, we prepared some libraries from 27 negative CSF samples validated by both culture and mNGS test in the clinical laboratory of Peking Union Medical College Hospital, as well as 5 blank NC control samples, using the same batch of reagents and protocol as for the BECLEAN premodeling procedure. When we used the decontam R package to identify background contaminants from the data set from those samples, we found that DNA concentrations of 4 blank NC and 8 negative CSF samples were below the detection limit of the Qubit double-stranded DNA (dsDNA) high-sensitivity assay, and so the effect of decontam modeling was compromised by the inaccurate DNA quantification solution (see Fig. S5). Surprisingly, library concentration was a better indicator of sample biomass and more suitable for decontam modeling under such a situation (Fig. 4A to C, left panels). Moreover, decontam identified well the same contaminants in the data set consisting of blank NC and negative CSF samples as BECLEAN did (Moraxella osloensis [Fig. 4A], Escherichia coli [Fig. 4B], and Pseudomonas aeruginosa [Fig. 4C]; left panels show decontam results and right panels shown BECLEAN results). All NC and negative CSF samples were within the *Z* ± 3 region of the 3 preestablished models of BECLEAN, consistent with the distribution of the modeling data set and suggesting stability of the background profile across different data sets under the same experimental setting (Fig. 4, right panels, and Fig. S4B).

Afterwards, we spiked a diluted bacteria culture suspension (Moraxella osloensis, Escherichia coli, or Pseudomonas aeruginosa, all identified by decontam and BECLEAN) into several separate negative-control CSF samples to mimic three kinds of pathogen-positive clinical specimens: (i) 1 Moraxella osloensis-positive sample with high biomass, (ii) 8 Escherichia coli-positive samples with high biomass, and (iii) 6 Pseudomonas aeruginosa*-*positive samples with low biomass. To mimic a scenario in which various types of samples of diverse origins might be tested and analyzed together in a clinical diagnostic laboratory, these positive samples were added into the aforementioned data set and analyzed by decontam and BECLEAN. It can be seen that the signals of spiked-in contaminants showed an outlier pattern (Fig. 4A to C, red dots in middle and right panels). BECLEAN well identified all positive samples (*Z* ≥ 3) based on the preestablished model (Fig. 4A to C, right panels). However, decontam failed to recognize spiked-in Pseudomonas aeruginosa and still regarded them as contaminants (Fig. 4C, middle panel), because the difference between the positive and negative signals was not remarkable (*Z* score range, 3.09 to 4.64) when only a small amount of Pseudomonas aeruginosa was added into negative CSF samples. On the other hand, the *P* values of frequency-based models for Moraxella osloensis and Escherichia coli increased markedly (Fig. 4A and B, middle panels) after the incorporation of positive samples, indicating that decontam’s statistical model noted the presence of outliers and found that the signals no longer fit the contaminant pattern well. Notably, decontam only provides a group analysis result of whether a taxon within all samples meets the characteristics of contamination; it is not able to identify whether a taxon within each sample is contaminant. To sum up, decontam may be vulnerable when distinguishing contamination from true microbial taxa for analysis of data sets generated from different clinical sample types harboring the same pathogens of widely different abundances. Besides, when the positive signal in a real sample is close to the contaminant pattern, it will be difficult for decontam to distinguish them as well.

### Background contaminants identified by BECLEAN under different circumstances.

Previous studies revealed that changes in reagents, consumables, environment, technicians, equipment, or protocols could critically impact sequence-based microbiome analyses (2, 3, 16, 17, 21), which may be an important caveat for effective implementation for BECLEAN. Therefore, it is necessary to regularly monitor the contaminant profile following any significant changes in experimental variables. We proposed to sequence several low-biomass NC samples (the artificial DNA with xenobiotic sequence used in the premodeling process) in each run and compare, with PCoA and PERMANOVA, those samples with similar samples used for modeling. If there were significant differences in the background composition between the two batches of low-biomass NCs, it would be necessary to revalidate the BECLEAN model.

**FIG 5:**
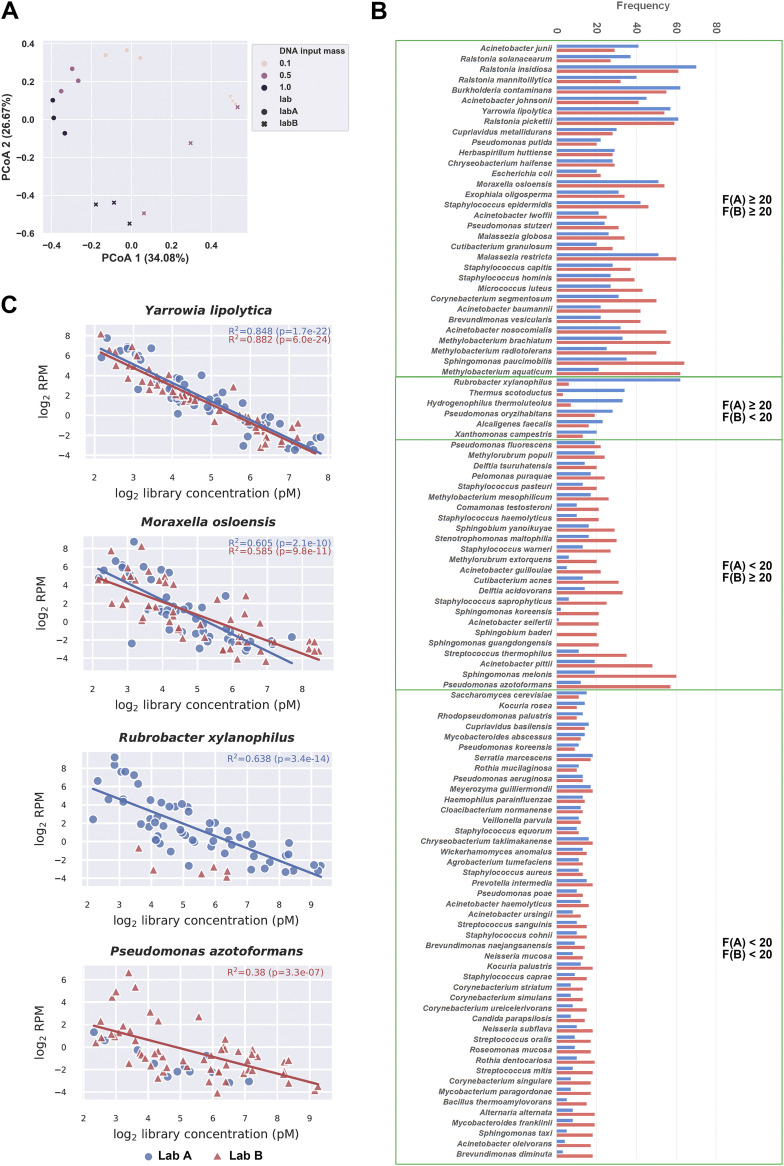
Background contaminants identified by BECLEAN under different circumstances. (A) PCoA based on Bray-Curtis distances of same artificial DNA samples with low input mass sequenced in two labs. Colors of data points gradually deepen as DNA input mass increases from 0.1 ng to 1 ng. Dots and crosses represent libraries prepared in lab A and lab B, respectively. The microbial profile showed significant differences between the 2 labs. (B) The frequency distribution of all the background contaminants identified in BECLEAN models in lab A and lab B. Taxa were grouped and labeled according to their occurrence frequency in two models (green boxes). F, occurrence frequency; A, lab A; B, lab B. (C) BECLEAN modeling was established in lab A and lab B, using the same batch of library preparation reagents. The linear regressions associating log_2_-transformed library concentration with log_2_-transformed RPM of Yarrowia lipolytica, Moraxella osloensis, Rubrobacter xylanophilus and Pseudomonas azotoformans are described with the *R*^2^ and *P* values.

To test the above-mentioned proposal and evaluate the reproducibility of BECLEAN, we decided to perform a second modeling in a second lab (lab B) using another training data set (*n* = 72), with the same batch of reagents and protocol for lab A premodeling. In addition, before premodeling in lab B, several artificial DNA samples with low input mass were used for library preparation in both lab A and lab B, with the same batch of reagents and protocol. After those NC libraries from 2 labs were sequenced and analyzed, PCoA based on Bray-Curtis distances demonstrated that the microbial profiles between the 2 labs might be somehow different (Fig. 5A). The BECLEAN model was then reestablished in lab B. The frequency distributions of all the background contaminants identified by BECLEAN models in lab A and lab B are listed in Fig. 5B. The background contaminants identified in the two models can be classified into groups according to their frequency of occurrence: (i) 32 species with relative high occurrence frequency in both models (Fig. 5B, green box on the top), which probably originated from a common source of contaminants; (ii) contaminants with occurrence frequencies that differed greatly between the two models (Fig. 5B, the two boxes in the middle), indicating a different background composition between the 2 labs; and (iii) contaminants with relatively low occurrence frequencies in both models (Fig. 5B, box on the bottom), which may be more susceptible to the random variation. In total, 56 species were thought to be significant background contaminants in lab B, whereas 38 contaminants were identified in lab A. The frequency and linear regression model of Yarrowia lipolytica and Moraxella osloensis in lab A was comparable to that in lab B (Fig. 5C), implying common sources of contaminants. We also observed unique background species between the labs (e.g., Rubrobacter xylanophilus and Pseudomonas azotoformans) (Fig. 5C), implying that part of the microbial flora differed between laboratories.

## DISCUSSION

The successful use of shotgun sequencing in identifying Leptospira santarosai infection in the cerebrospinal fluid of a 14-year-old boy in 2013 (22) set the pedestal for wide application of metagenomic sequencing in the diagnosis of infectious disease, particularly for infections of the central nervous system (18, 23–25). However, the challenge of low biomass in CSF limits the application of metagenomic sequencing (26). In addition, low pathogen loads in CSF samples also constrain efficient nucleic acid extraction, as the samples are frequently laden with microbial contaminants. To address background contamination, Zinter et al. and Davis et al. developed a novel statistical approach for identification and removal of contaminant sequences, either by incorporation of standardized RNA controls to calculate sample input mass and the studentized residual of certain taxon (5) or by bioinformatics analysis of signatures of contaminant DNA (19). These approaches are both based on the principle of inverse proportionality of the microbe sequence read count to total sample input mass. These methods do not rely on presequencing of environmental or reagent-only samples and readily fit into existing mNGS workflows. In fact, the R package decontam employed by Davis et al. was able to identify contaminants well in a data set consisting of blank NC and negative CSF samples. However, when analyzing a mimic data set that incorporated contaminant-spiked samples, decontam noted the abnormal signals of a Moraxella osloensis-positive sample and Escherichia coli-positive samples but failed to recognize spiked-in Pseudomonas aeruginosa, owing to its similar pattern to contaminants. The decontam is a group analysis tool and will calculate the likelihood of whether a taxon within all samples meets the characteristics of contamination; however, it is not suitable for individual analysis. In real-life medical practice, it is common that some microorganisms detected by mNGS are both true pathogenic positives and background contaminants (e.g., Escherichia coli, Pseudomonas aeruginosa, and Acinetobacter baumannii). Concerns should be taken for such intractable cases, as those microorganisms may be easily misclassified. In addition, decontam or the approach developed by Zinter et al. are unsuitable for analyzing data sets generated from different clinical samples of diverse origins at the same time. Clinical samples of different types can harbor various resident flora containing the same pathogens at different abundance levels. Such a complicated situation will affect the effectiveness of these modeling approaches to identify contamination. Take Escherichia coli as an example: while it is more likely to be a low-abundance contaminant for blood samples due to its ubiquitous existence in the lab environment, Escherichia coli is one of the major inhabitants of the intestines, with high abundance. Thus, the two approaches mentioned above are not applicable to analyze the presence of Escherichia coli in a mixed data set generated from blood, CSF, anal swabs, and stool samples at the same time, because of irregular distributions of abundance of the bacteria in such samples.

Additionally, a short turn-around time of pathogen identification in clinical settings is necessary for mNGS-based methods. In addition to the complicated wet-lab procedure, the collection of a large batch of specimens and controls is time-consuming. Accordingly, there is a continuous search for rapid and accurate clinical mNGS solutions for identification of pathogens, even for a small number of samples. As the approaches developed by Zinter et al. (5) and Davis et al. (19) both depend on large metadata sets generated from large biological samples or controls, without data accumulation it may be unrealistic to apply these methods to quantify and eliminate background noise in small-scale clinical metagenomic data. To address these inherent limitations, we determined the BECLEAN background premodeling before experiments to have preunderstanding of possible contaminants, to minimize the interference in metagenomics of test biological samples. The motivations for decontam and BECLEAN are somewhat divergent, with decontam focusing on large-scale observational microbiome studies and BECLEAN focusing on small-scale clinical diagnostic use. Although premodeling entails additional cost, it allows timely identification of pathogens not only for small numbers of samples but also for varied samples. In addition, it eliminates the need for incorporation of spike-in DNA or RNA controls in the sample.

In addition to the spike-in approaches employed by Zinter et al., alternative ways to measure sample DNA mass are Qubit assays or UV spectroscopy (e.g., using a NanoDrop spectrophotometer). We found that it was not ideal to measure sample DNA mass for BECLEAN or decontam modeling when using Qubit assays or UV spectroscopy (see Fig. S5), especially when the input mass of many samples was lower than the LOD of those approaches and hard to measure. It is not necessary to accurately measure the sample input mass for modeling, as BECLEAN relies on the inverse linear relationship between microbial sequencing reads and library concentration. Because we employ PCR-free-based approaches for library preparation to reduce PCR bias that may affect calculations of abundance (21), it is necessary to precisely measure the concentration of each library before pooling them. It is more advantageous to use library concentration for linear regression modeling, both for BECLEAN and decontam (Fig. 4), because in some instances the input mass is immeasurable due to an ultralow biomass of many samples, while the LOD for the KAPA library quantification kit reaches as low as 0.0002 pM.

While BECLEAN significantly minimizes the noise in metagenomic sequencing, it does not completely remove the background reads incorrectly aligned to the taxa genome with high similarity, and it does not eliminate cross-contamination arising from index switching or other causes, which can nonetheless be resolved by other approaches (27). Because different bioinformatics databases or pipelines for microbiome analysis impact contaminant profiling and downstream interpretation, it is necessary to use the same bioinformatics database and pipeline for BECLEAN premodeling and subsequent decontamination.

In addition, BECLEAN can only identify the most probable background within the scope of the wet-lab procedure employed during premodeling, and therefore it cannot correctly identify contamination introduced at sample collection or pretreatment steps (Xanthomonas campestris) (Fig. 3A and B). Moreover, a BECLEAN model is susceptible to batch effect, which may compromise its performance when the reagents in the library preparation step used for premodeling change. Changes in laboratory environmental variables like reagents, equipment, staff members, and experimental protocols can alter the composition of the laboratory background and thus impact the effectiveness of the pretrained model. BECLEAN is not a “once and done” solution for filtering metagenomics data against identified laboratory contamination; rather, it requires regular monitoring and revalidation over time. We strongly recommend use of the same batch of reagents, consumables, and protocols for premodeling and sample test. PCoA and PERMANNOVA of a few low-biomass NC samples in each run may provide an indicator for the adjustment of the model as well. Finally, a requirement of using a PCR-free library preparation approach is also a limitation of BECLEAN, because the true library concentration will alter in PCR and purification steps for the PCR-based methodology.

In summary, BECLEAN is an effective statistical tool that can separate contaminants from true positives. With the features of its premodeling process and individual analysis interpretation, BECLEAN provides a time-saving and convenient mNGS noise-filtering solution for clinical laboratory staff. Along with decontam and other existing methods, BECLEAN is a complementary method for identifying contaminants, particularly in various clinical settings.

## MATERIALS AND METHODS

### Establishment of premodeling training set for BECLEAN.

A 2,672-bp artificial DNA (sequence is provided in Table S1 in the supplemental material) was first designed, synthesized, and thereafter amplified using PCR (with PrimeSTAR HS DNA polymerase, TaKaRa catalog number R044). It was then purified using magnetic beads (Matridx catalog number MD005T). The experiments were performed in a clean and controlled environment (a hood in PCR rooms with constant temperature and humidity). Qubit fluorometric quantitation was performed on the final amplicons (Qubit dsDNA HS assay kit, Thermo Fisher catalog number Q32854), which were serially diluted (5 pg/μL to 5 ng/μL) in subsequent experiments. On the other hand, we used 20 μL of artificial DNA (different input masses of 0.1, 0.5, 1, 2, 5, 10, 30, and 100 ng) for the automated library preparation independent of PCR (Matridx catalog number MD014). Enzymatic fragmentation, end repairing, terminal adenylation, and adaptor ligation were performed by an automated NGSmaster cartridge-based library preparation system (Matridx catalog number MAR002) (28). All samples were independently extracted and underwent library preparation in separate cartridges. The complete training set for BECLEAN consisted of 9 (or 10) duplicate groups of 8 libraries of different biomass input. The concentrations of the 72 (or 80) training set libraries were determined by KAPA qPCR (Roche) according to the manufacturer’s instructions, followed by equimolar pooling. Two rounds of 75-bp single-end sequencing were performed using the NextSeq 500 platform based on the NextSeq High reagent kit v2 (Illumina), to an average depth of 10 million reads per library (~32 to 40 libraries per run).

### Verification using contaminant-spiked samples.

Yarrowia lipolytica, Moraxella osloensis, Acinetobacter johnsonii, and Acinetobacter junii were collected and cultured at Peking Union Medical College laboratories. The concentration of each bacteria or yeast suspension was standardized by comparing the turbidity of the suspension with that of a 0.5 McFarland standard. Then, 4 liquid suspensions were mixed together. The mixture containing 1 × 10^6^ CFU/mL of Yarrowia lipolytica, 1 × 10^8^ CFU/mL of Moraxella osloensis, 1 × 10^8^ CFU/mL of Acinetobacter johnsonii, and 1 × 10^8^ CFU/mL Acinetobacter junii was thereafter serially diluted (range from 1:0 to 1:10^6^). Except for the negative-control group, 0.01 mL of the different dilutions were spiked with either 0.99 mL of 1 × 10^4^ (mimicking low biomass input) or 1 × 10^5^ (mimicking median biomass input) of immortalized human T lymphocytes to a final volume of 1 mL. Finally, the contaminant-spiked samples with or without the spike-in mixture underwent ultrasonic disruption for 5 min, followed by DNA extraction, enzymatic fragmentation, end repairing, terminal adenylation, and adaptor ligation using the NGSmaster cartridge-based automation library preparation system (Matridx catalog number MAR002) (28). The reagents included a nucleic acid extraction cartridge (Matridx catalog number MD014) and DNA library preparation kit (Matridx catalog number MD001T) with the same batch as used in modeling. The concentrations of prepared NGS libraries were determined by KAPA qPCR followed by manual normalization and pooling. Sequencing of the 75-bp single end with 8-bp dual index was performed at a median depth of 10 million reads per sample.

### CSF sample collection and validation using these samples.

A single-blinded, retrospective study was performed to evaluate BECLEAN performance. Sample processing and preparation for mNGS and metagenomic sequencing analysis were also blinded experiments. Unblinding was performed after all the sequencing and bioinformatics analysis were finished. Twenty-eight CSF samples were collected in the clinical laboratory of Peking Union Medical College Hospital between January 2019 and July 2020. Thirteen samples were from patients with suspected central nervous system (CNS) infections, whereas the others were collected from patients diagnosed with non-CNS infection (see Table S2). Bacterial and fungal cultures were performed in-house. The residual CSF samples were used for mNGS detection or PCR-based testing after routine clinical testing in the microbiology laboratory. Positive pathogen(s) and samples must be either detected by culture or qPCR (gold standard) or be adjudicated from clinician input, which was performed before mNGS results. Negative samples were either CNS infection excluded or with no definitive diagnosis. Negative samples were also culture or qPCR negative. Convenience sampling for the collection was performed based on availability (sufficient sample residual volume). Residual samples were stored at −80°C and tested within 14 days of collection. Saline was added into each 400- to 800-μL CSF sample to a final volume of 1 mL, followed by a 5-min ultrasonic disruption. The DNA library was prepared without PCR amplification using the automatic NGSmaster cartridge-based system (Matridx catalog number MAR002) (28), which is composed of a nucleic acid extraction cartridge (Matridx catalog number MD014) and DNA library preparation kit (Matridx catalog number MD001T). The concentrations of the NGS libraries were determined using KAPA qPCR, and later equimolar library solutions were pooled together. Sequencing of the 75-bp single end with 8-bp dual index was performed with the NextSeq 500 platform, based on the NextSeq High reagent kit v2 (75 cycles; Illumina). The median depth was 22.7 million reads per sample.

### Comparison between BECLEAN and decontam.

The isContaminant function in the decontam R package (version 1.6.0) was used to analyze the relative abundance of three background contaminants in the data set. A default threshold (0.1) was applied to the *P* value of the frequency, prevalence, and combined methods returned by the isContaminant function. The frequency patterns were plotted with the plot_frequency function provided by decontam. Blank NC and negative CSF samples and contaminant-spiked samples on the frequency plots were colored using R package ggplot2 (version 3.3.3) to better distinguish these samples (29).

### Bioinformatics analysis.

Quality control was performed on the multiplex sequence reads for each sample using an in-house tool, where those containing >5 nucleotide adapters at the end, with more than two undefined bases, and those containing >10% of bases with a quality score of 2 were all discarded. Clean reads were aligned to a human-specific database constructed from Homo sapiens sequences in the NCBI Nucleotide (NT) database (downloaded on 6 February 2020) using bowtie2 (version 2.3.5.1; “--very--sensitive --ignore-quals -k 50”) (30). Then, the remaining nonhuman reads were aligned to a microbial database based on NT data supplemented with the sequences from RefSeq, using bowtie2, which assigned taxonomic identifiers (taxID) to each read. Taxa with >10 reads were validated by BLAST (blastn; version 2.9.0+; “-qcov_hsp_perc 70 -perc_identity 90”) against NT (31). The large index of bowtie2 was built on a server with 512 CPUs and 1.5 TB of RAM with the command line parameter “--large-index --threads 48.”

### BECLEAN background species profiling.

For BECLEAN background species profiling, the candidate species were screened based on their relative frequency distribution in the training set. To distinguish relatively stable contaminants (i.e., microbes derived from reagents, experimental processing, or laboratory environment) from episodic contaminants, species identified in fewer than 20 samples were first excluded. A linear regression model between the logarithm of RPM to base 2 and the logarithm of library concentration to base 2 was then established for stable contaminants using the least-squares approach, which produced a series of model parameters and training set metrics: slope (*S*), intercept (*I*), coefficient of determination (*R*^2^), and average log_2_ library concentration (*lC*_mean_). Because the log_2_ RPM for each species in a sample has a minimum limit of log_2_[(1 × 10^6^ reads)/(total reads)], the linear relationship was not conformed when the log_2_ RPM reached this extreme. As such, data points outside the linearity range were excluded to establish the true relationship between RPM and library concentration.

After modeling, the log_2_ RPM of each background candidate species was normalized based on the log_2_-transformed library concentration, using the following formula: *lR*_norm_ = *lR* + *S* × (*lC*_mean_ − *lC*), where *lR* denotes the log_2_ RPM and *lC* denotes the log_2_-transformed library concentration. Subsequent analyses were only performed on species whose model residual, *lR* − (*S* × *lC* + *I*), passed the normality test (*P* > 0.05).

### Background filtering.

For each species in a sample, the *Z*-score was calculated to describe the deviation from the model-predicted value. *Z*-scores measure the probability a species is a background contaminant or constitutive in the sample. *Z*-scores were calculated as *Z* = (*lR*_norm_ − *lR*_mean_)/SD, where *lR*_norm_ denotes the normalized log_2_ RPM, *lR*_mean_ stands for average normalized log_2_ RPM of the training set, and SD is the standard deviation of the normalized log_2_ RPM of the training set. The cutoff value for the *Z*-score was 3, to distinguish background contamination from the constitutive sample microbiome (Fig. 1G).

### Statistical analysis.

Statistical analysis were performed using Python. The linear least-squares regression for modeling was carried out using the “linregress” function in the SciPy package (32). Normality test of *lR*_norm_ was based on D’Agostino’s method, and Pearson’s test was performed using the “normaltest” function from SciPy. We used Python scipy.stats.normaltest (kurtosis and skew test) to test the hypothesis of normal distribution of each taxa identified in BECLEAN premodeling process. Sample size of 20 is the minimum value that meets the requirement of the kurtosis test. Significance level was *P* < 0.05. PCoA and PERMANOVA was performed using the “pcoa” and “permanova” functions from the skbio package.

### Ethics approval and consent to participate.

The study was reviewed and approved by the Human Research Ethics Committee of Peking Union Medical College Hospital (S-K1186). This project did not affect the normal diagnosis and treatment of patients. Written informed consent for participation was not required for this study, in accordance with the national legislation and the institutional requirements.

### Data availability.

Data files are available in the Sequence Read Archive under BioProjects PRJNA665328, PRJNA665350, and PRJNA788644. The ipython notebook and all necessary input data to reproduce the analyses in this article are available in a GitHub repository, https://github.com/bioinfo-matridx/BECLEAN.
