# Supplementary material for: Background Filtering of Clinical Metagenomic Sequencing with a Library Concentration-Normalized Model
Source: Microbiol Spectr. 2022 Sep 22;10(5):e01779-22. doi: 10.1128/spectrum.01779-22 (PMC9603461; doi:10.1128/spectrum.01779-22)
Supplement: Supplemental file 1 — Fig. S1 to S5. Download spectrum.01779-22-s0001.pdf, PDF file, 6.4 MB [file spectrum.01779-22-s0001.pdf]

Supplemental Material

Supplemental file 1: Supplemental Figure Dataset 1 - Linear regression models of all taxa with frequency of occurrence ≥ 20 for Figure1 training dataset (n=72).

Supplemental file 2: Supplemental Figure Dataset 2 - A histogram of the residual of the model fits informing the linear regression between log2-transformed RPM and log2-transformed library concentration for 38 background species.

Supplemental file 3: Supplemental Figure Dataset 3 - Coverage maps of 38 background species identified in lab A dataset (automated protocol for library preparation).

Supplemental file 4: Supplemental Figure 4-BECLEAN pre-modeling analysis before the comparison with decontam

Supplemental file 5: Supplemental Figure 5-Decontam frequency-based model analysis of 3 contaminant species using sample DNA concentration

Supplemental file 6: Supplemental Table 1- Sequence of spike DNA & qPCR Primers

Supplemental file 7: Supplemental Table 2- Clinical information and detailed test results of 28 CSF samples

Supplemental Figure 1. Linear regression models of all taxa with frequency of occurrence ≥ 20 for Fig. 1 training dataset (n=72), related to Figure 1.

The linear regressions associating log_2-transformed library concentration (or sample input mass) with log_2-transformed RPM of identified species are described in each panel. The model R^2 and p value are marked in the top right corner of each panel.

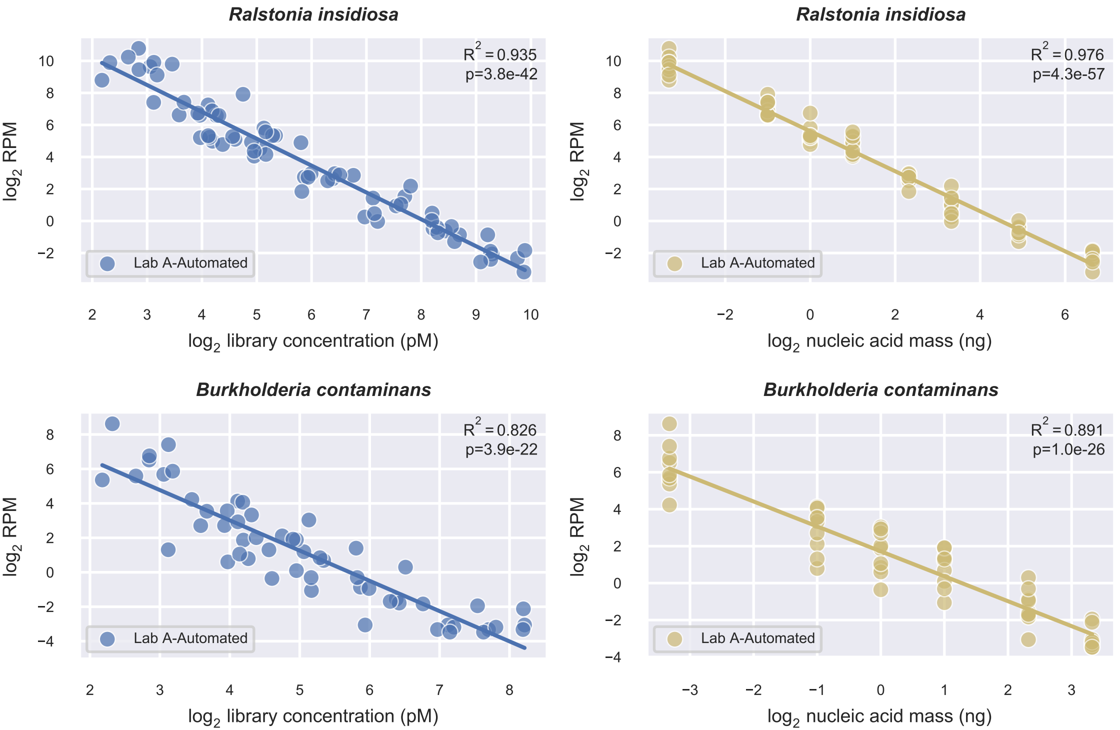

Ralstonia insidiosa
R^2 = 0.935
p=3.8e-42
Lab A-Automated
log2 RPM
log2 library concentration (pM)
Ralstonia insidiosa
R^2 = 0.976
p=4.3e-57
Lab A-Automated
log2 RPM
log2 nucleic acid mass (ng)
Burkholderia contaminans
R^2 = 0.826
p=3.9e-22
Lab A-Automated
log2 RPM
log2 library concentration (pM)
Burkholderia contaminans
R^2 = 0.891
p=1.0e-26
Lab A-Automated
log2 RPM
log2 nucleic acid mass (ng)

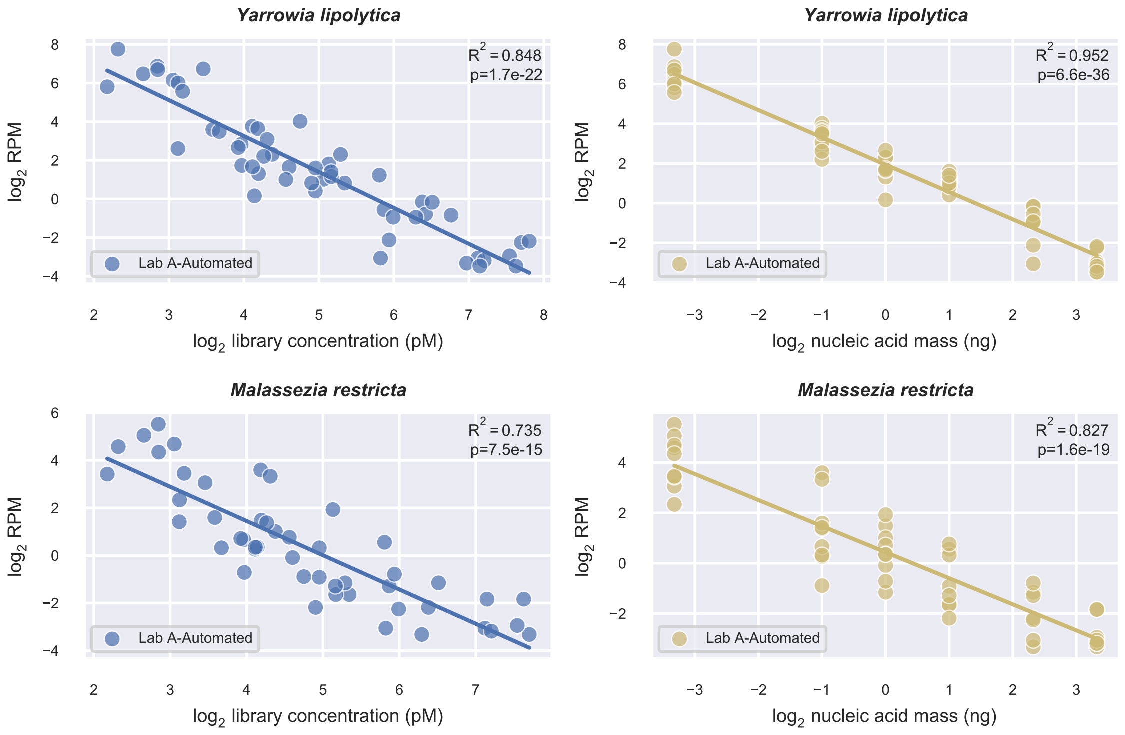

Yarrowia lipolytica
$R^2 = 0.848$
p=1.7e-22
Lab A-Automated
$\log_2$ RPM
$\log_2$ library concentration (pM)
Yarrowia lipolytica
$R^2 = 0.952$
p=6.6e-36
Lab A-Automated
$\log_2$ RPM
$\log_2$ nucleic acid mass (ng)
Malassezia restricta
$R^2 = 0.735$
p=7.5e-15
Lab A-Automated
$\log_2$ RPM
$\log_2$ library concentration (pM)
Malassezia restricta
$R^2 = 0.827$
p=1.6e-19
Lab A-Automated
$\log_2$ RPM
$\log_2$ nucleic acid mass (ng)

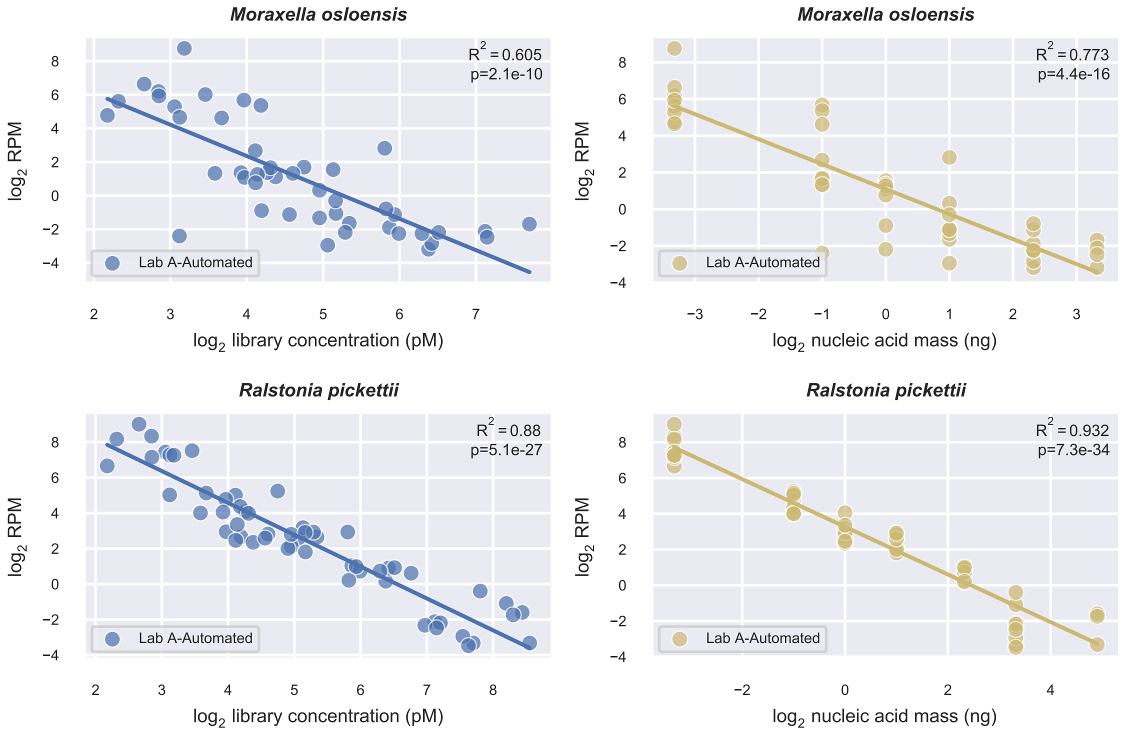

Moraxella osloensis
R^2 = 0.605
p=2.1e-10
Lab A-Automated
log2 RPM
log2 library concentration (pM)
Moraxella osloensis
R^2 = 0.773
p=4.4e-16
Lab A-Automated
log2 RPM
log2 nucleic acid mass (ng)
Ralstonia pickettii
R^2 = 0.88
p=5.1e-27
Lab A-Automated
log2 RPM
log2 library concentration (pM)
Ralstonia pickettii
R^2 = 0.932
p=7.3e-34
Lab A-Automated
log2 RPM
log2 nucleic acid mass (ng)

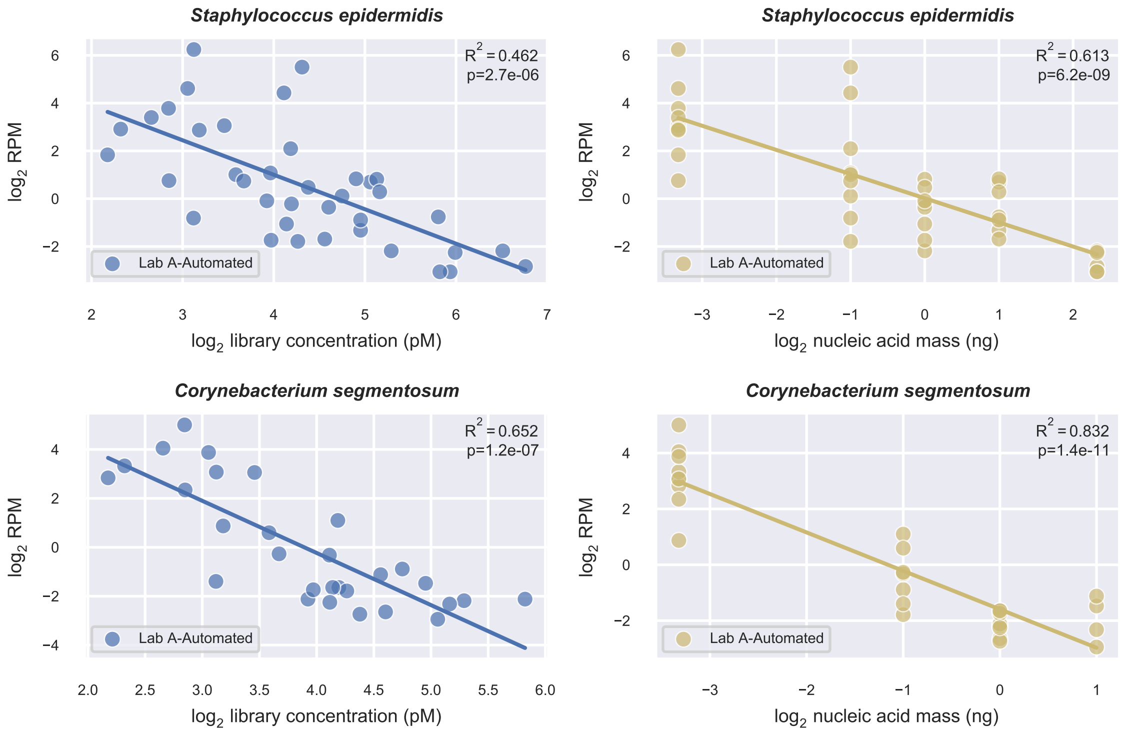

Staphylococcus epidermidis
R² = 0.462
p=2.7e-06
Lab A-Automated
log₂ RPM
log₂ library concentration (pM)
Staphylococcus epidermidis
R² = 0.613
p=6.2e-09
Lab A-Automated
log₂ RPM
log₂ nucleic acid mass (ng)
Corynebacterium segmentosum
R² = 0.652
p=1.2e-07
Lab A-Automated
log₂ RPM
log₂ library concentration (pM)
Corynebacterium segmentosum
R² = 0.832
p=1.4e-11
Lab A-Automated
log₂ RPM
log₂ nucleic acid mass (ng)

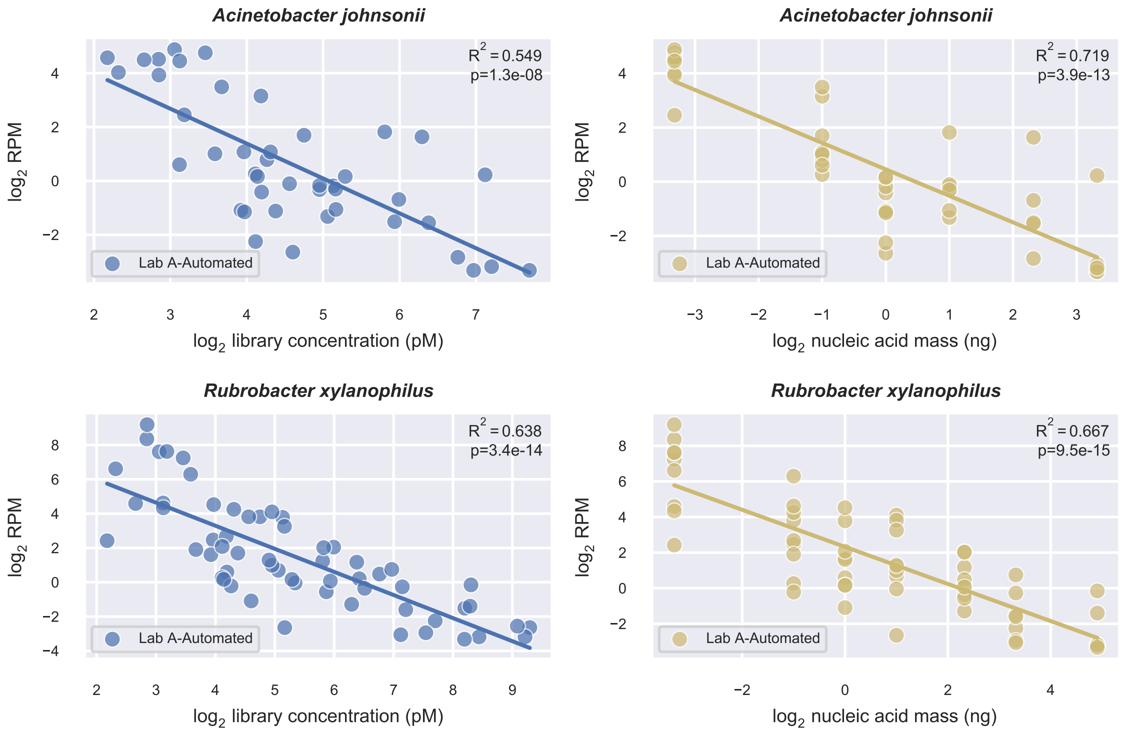

Acinetobacter johnsonii
R2 = 0.549
p=1.3e-08
Lab A-Automated
log2 RPM
log2 library concentration (pM)
Acinetobacter johnsonii
R2 = 0.719
p=3.9e-13
Lab A-Automated
log2 RPM
log2 nucleic acid mass (ng)
Rubrobacter xylanophilus
R2 = 0.638
p=3.4e-14
Lab A-Automated
log2 RPM
log2 library concentration (pM)
Rubrobacter xylanophilus
R2 = 0.667
p=9.5e-15
Lab A-Automated
log2 RPM
log2 nucleic acid mass (ng)

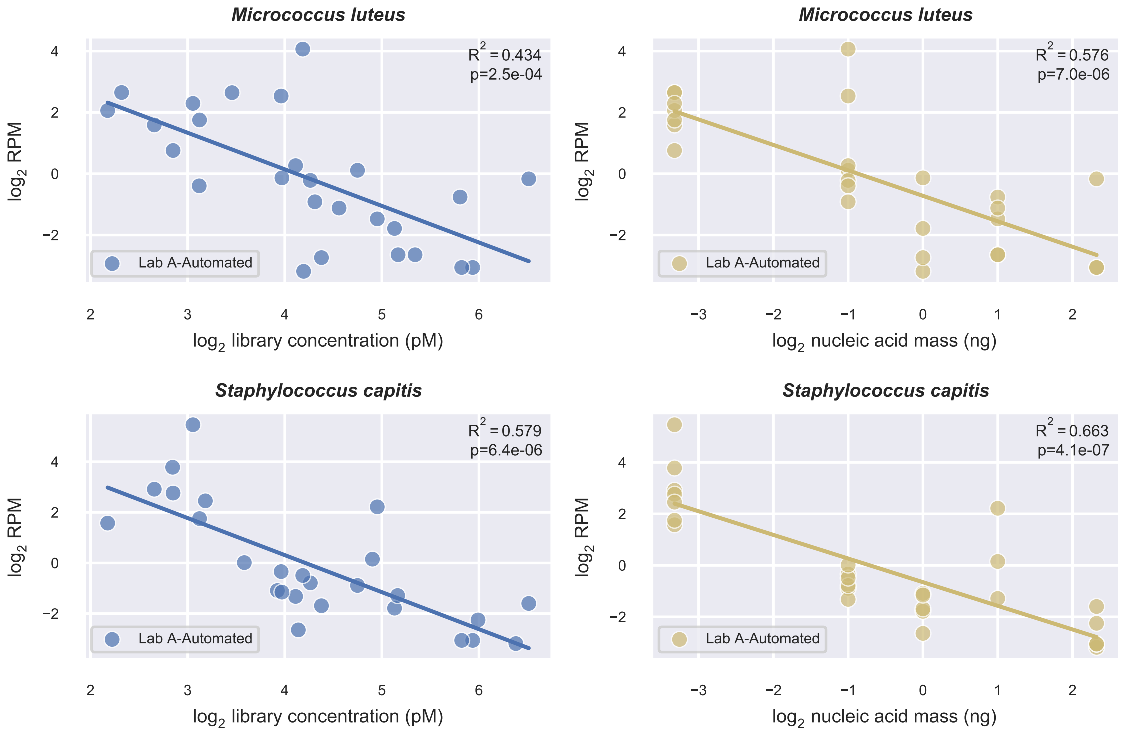

Micrococcus luteus
$R^2 = 0.434$
p=2.5e-04
Lab A-Automated
log2 RPM
log2 library concentration (pM)
Micrococcus luteus
$R^2 = 0.576$
p=7.0e-06
Lab A-Automated
log2 RPM
log2 nucleic acid mass (ng)
Staphylococcus capitis
$R^2 = 0.579$
p=6.4e-06
Lab A-Automated
log2 RPM
log2 library concentration (pM)
Staphylococcus capitis
$R^2 = 0.663$
p=4.1e-07
Lab A-Automated
log2 RPM
log2 nucleic acid mass (ng)

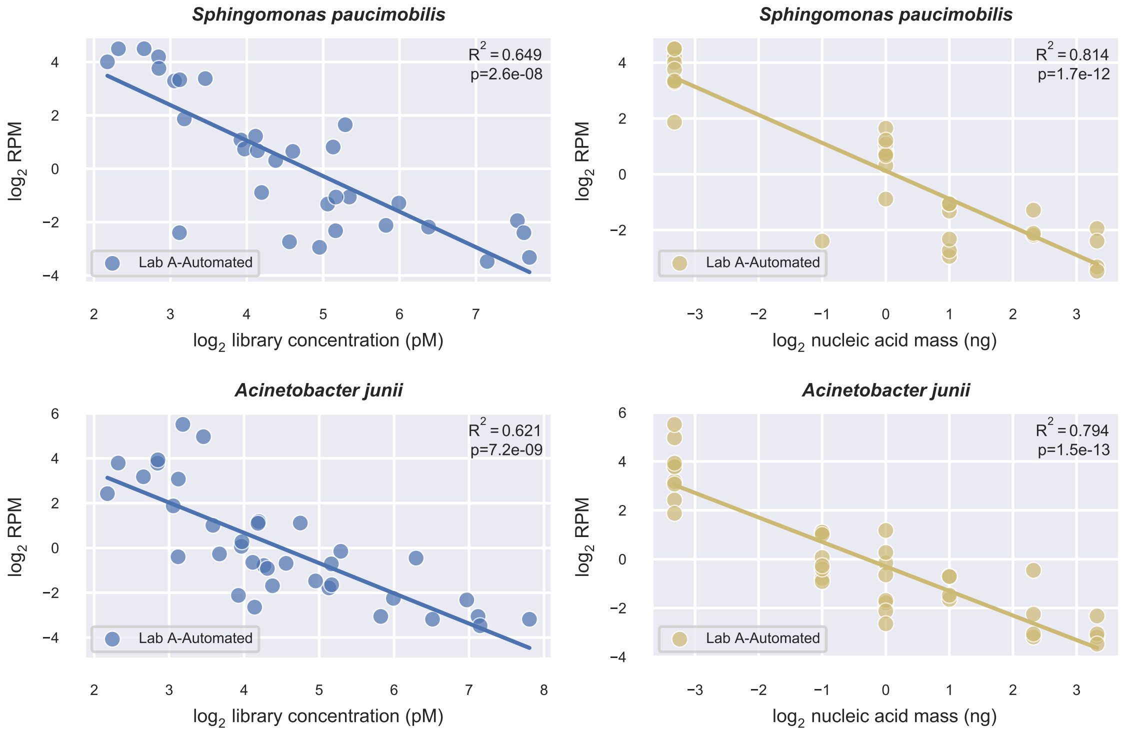

Sphingomonas paucimobilis
R^2 = 0.649
p=2.6e-08
Lab A-Automated
log2 RPM
log2 library concentration (pM)
Sphingomonas paucimobilis
R^2 = 0.814
p=1.7e-12
Lab A-Automated
log2 RPM
log2 nucleic acid mass (ng)
Acinetobacter junii
R^2 = 0.621
p=7.2e-09
Lab A-Automated
log2 RPM
log2 library concentration (pM)
Acinetobacter junii
R^2 = 0.794
p=1.5e-13
Lab A-Automated
log2 RPM
log2 nucleic acid mass (ng)

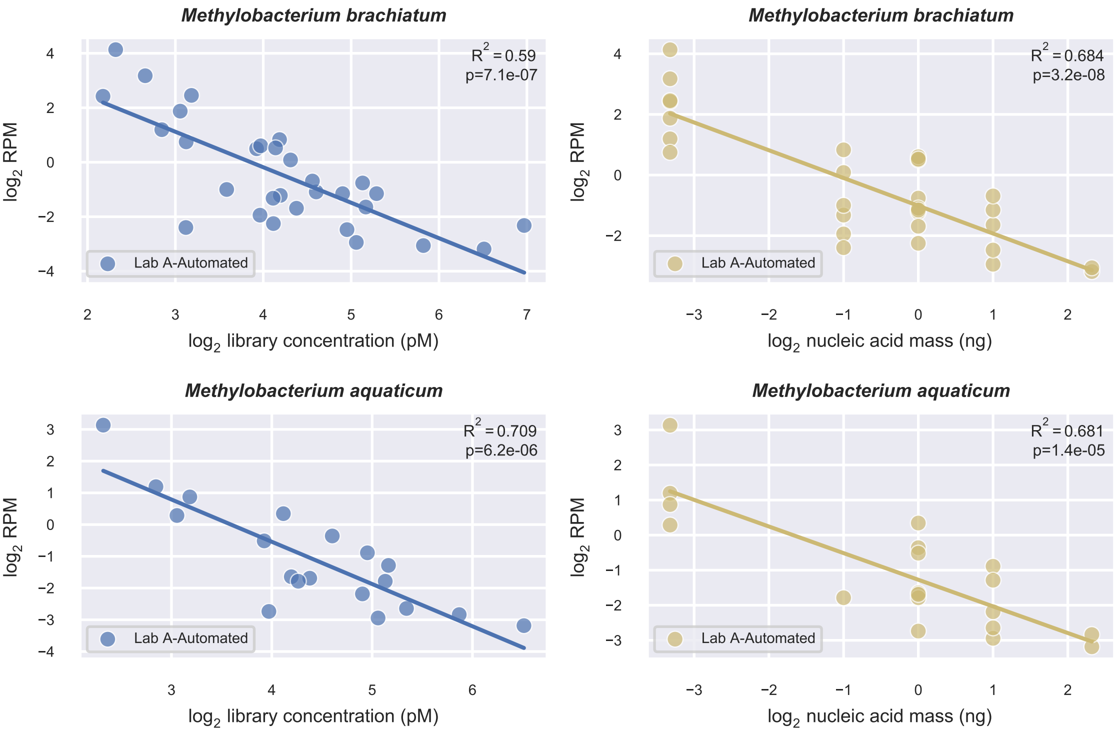

Methylobacterium brachiatum
R^2 = 0.59
p=7.1e-07
Lab A-Automated
log2 RPM
log2 library concentration (pM)
Methylobacterium brachiatum
R^2 = 0.684
p=3.2e-08
Lab A-Automated
log2 RPM
log2 nucleic acid mass (ng)
Methylobacterium aquaticum
R^2 = 0.709
p=6.2e-06
Lab A-Automated
log2 RPM
log2 library concentration (pM)
Methylobacterium aquaticum
R^2 = 0.681
p=1.4e-05
Lab A-Automated
log2 RPM
log2 nucleic acid mass (ng)

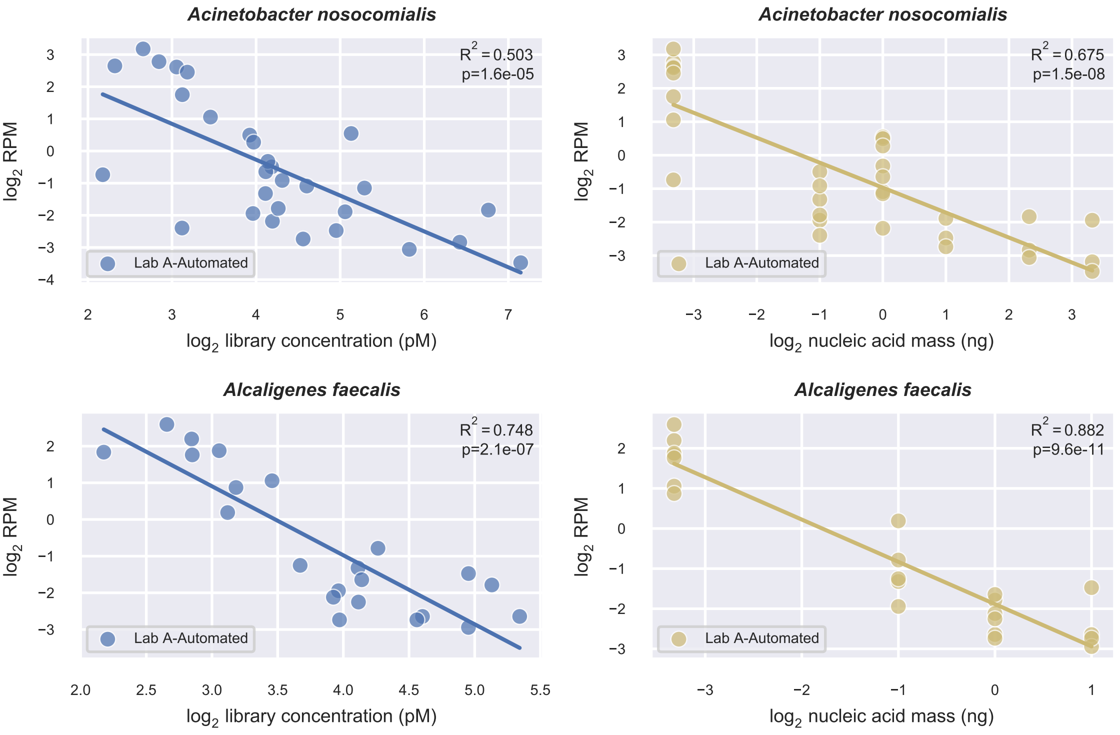

Acinetobacter nosocomialis
$R^2 = 0.503$
p=1.6e-05
Lab A-Automated
$\log_2$ RPM
$\log_2$ library concentration (pM)
Acinetobacter nosocomialis
$R^2 = 0.675$
p=1.5e-08
Lab A-Automated
$\log_2$ RPM
$\log_2$ nucleic acid mass (ng)
Alcaligenes faecalis
$R^2 = 0.748$
p=2.1e-07
Lab A-Automated
$\log_2$ RPM
$\log_2$ library concentration (pM)
Alcaligenes faecalis
$R^2 = 0.882$
p=9.6e-11
Lab A-Automated
$\log_2$ RPM
$\log_2$ nucleic acid mass (ng)

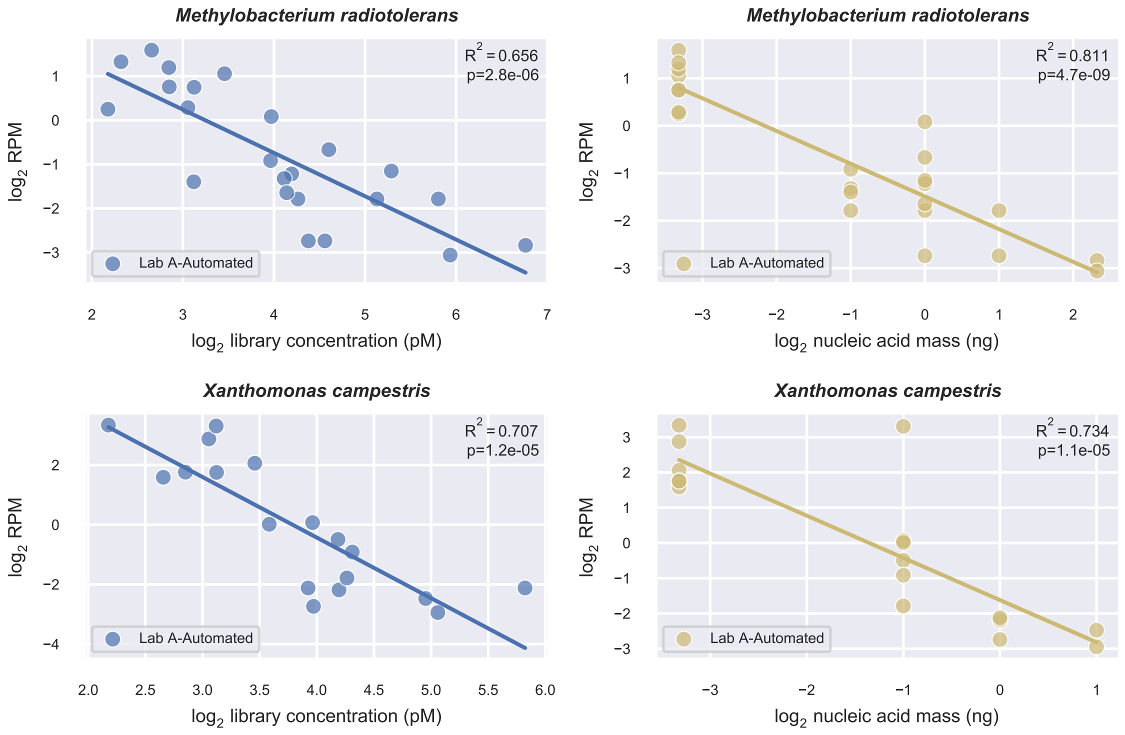

Methylobacterium radiotolerans
$R^2 = 0.656$
p=2.8e-06
Lab A-Automated
log2 RPM
log2 library concentration (pM)
Methylobacterium radiotolerans
$R^2 = 0.811$
p=4.7e-09
Lab A-Automated
log2 RPM
log2 nucleic acid mass (ng)
Xanthomonas campestris
$R^2 = 0.707$
p=1.2e-05
Lab A-Automated
log2 RPM
log2 library concentration (pM)
Xanthomonas campestris
$R^2 = 0.734$
p=1.1e-05
Lab A-Automated
log2 RPM
log2 nucleic acid mass (ng)

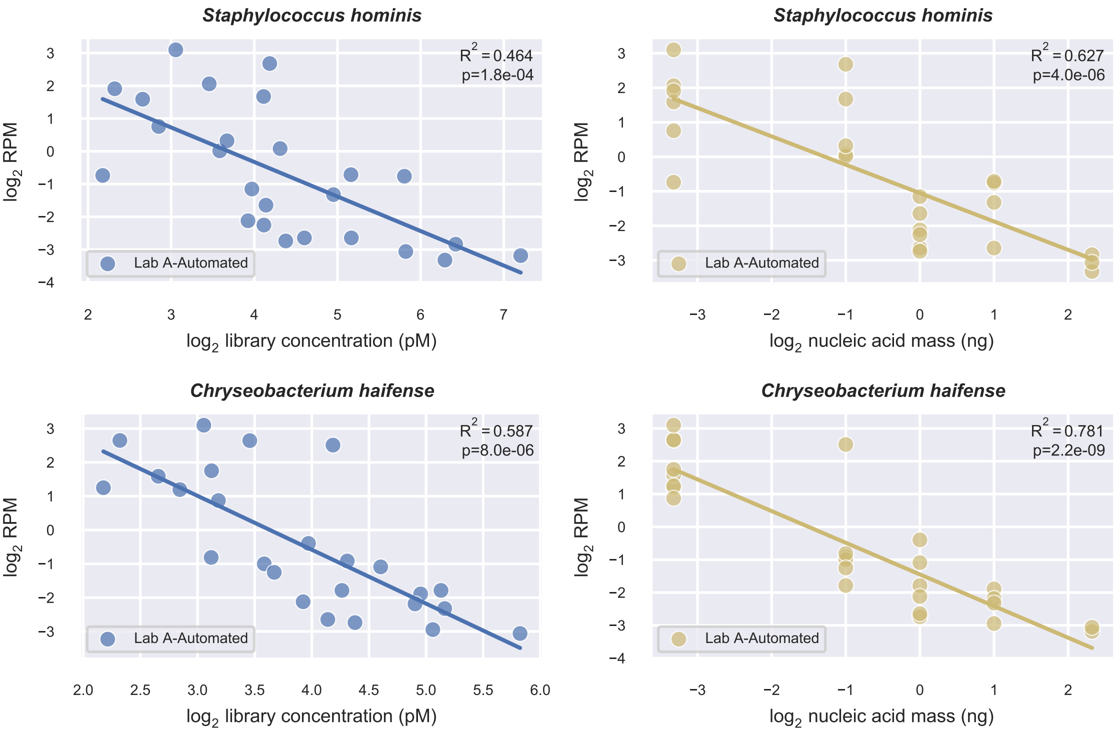
Staphylococcus hominis
$R^2 = 0.464$
p=1.8e-04
Lab A-Automated
$\log_2$ RPM
$\log_2$ library concentration (pM)
Staphylococcus hominis
$R^2 = 0.627$
p=4.0e-06
Lab A-Automated
$\log_2$ RPM
$\log_2$ nucleic acid mass (ng)
Chryseobacterium haifense
$R^2 = 0.587$
p=8.0e-06
Lab A-Automated
$\log_2$ RPM
$\log_2$ library concentration (pM)
Chryseobacterium haifense
$R^2 = 0.781$
p=2.2e-09
Lab A-Automated
$\log_2$ RPM
$\log_2$ nucleic acid mass (ng)

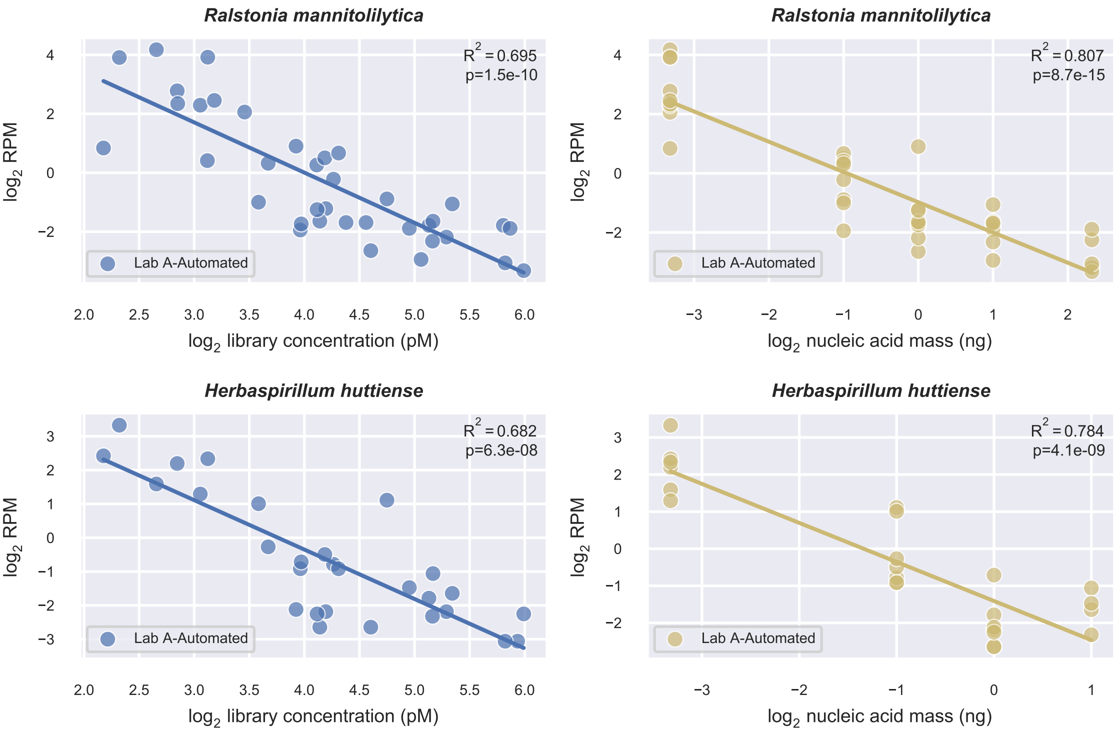

Ralstonia mannitolilytica
R^2 = 0.695
p=1.5e-10
Lab A-Automated
log2 RPM
log2 library concentration (pM)
Ralstonia mannitolilytica
R^2 = 0.807
p=8.7e-15
Lab A-Automated
log2 RPM
log2 nucleic acid mass (ng)
Herbaspirillum huttiense
R^2 = 0.682
p=6.3e-08
Lab A-Automated
log2 RPM
log2 library concentration (pM)
Herbaspirillum huttiense
R^2 = 0.784
p=4.1e-09
Lab A-Automated
log2 RPM
log2 nucleic acid mass (ng)

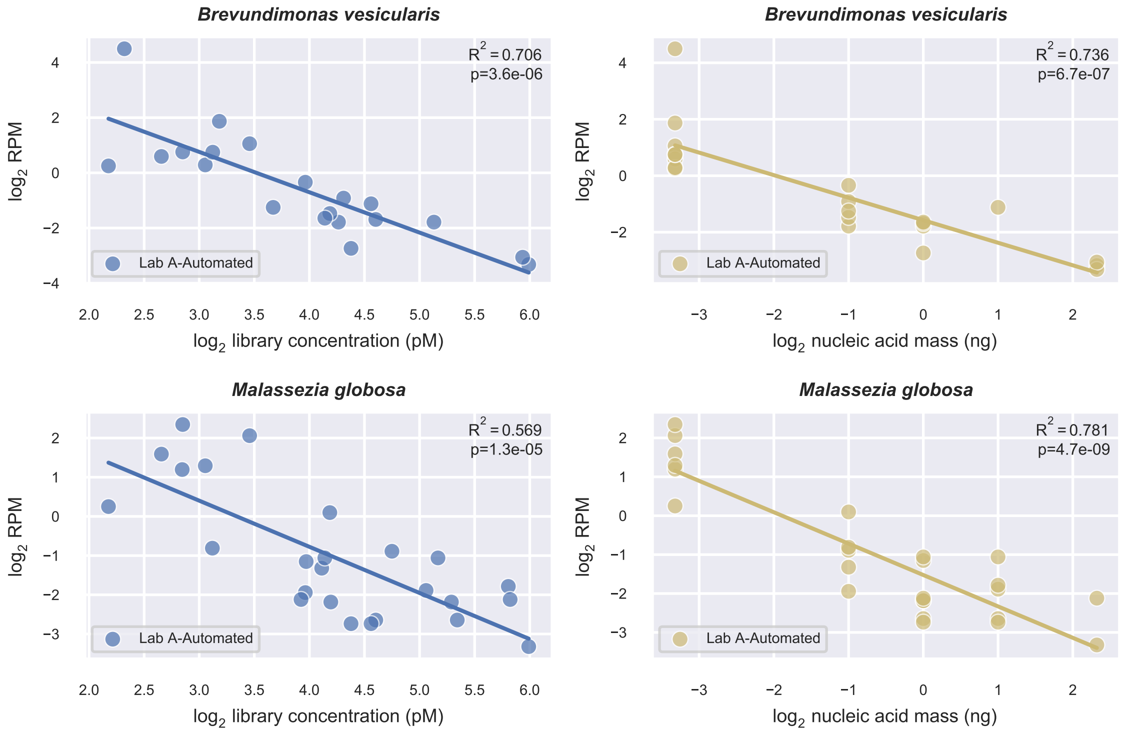
Brevundimonas vesicularis
R² = 0.706
p=3.6e-06
Lab A-Automated
log2 RPM
log2 library concentration (pM)
Brevundimonas vesicularis
R² = 0.736
p=6.7e-07
Lab A-Automated
log2 nucleic acid mass (ng)
Malassezia globosa
R² = 0.569
p=1.3e-05
Lab A-Automated
log2 RPM
log2 library concentration (pM)
Malassezia globosa
R² = 0.781
p=4.7e-09
Lab A-Automated
log2 nucleic acid mass (ng)

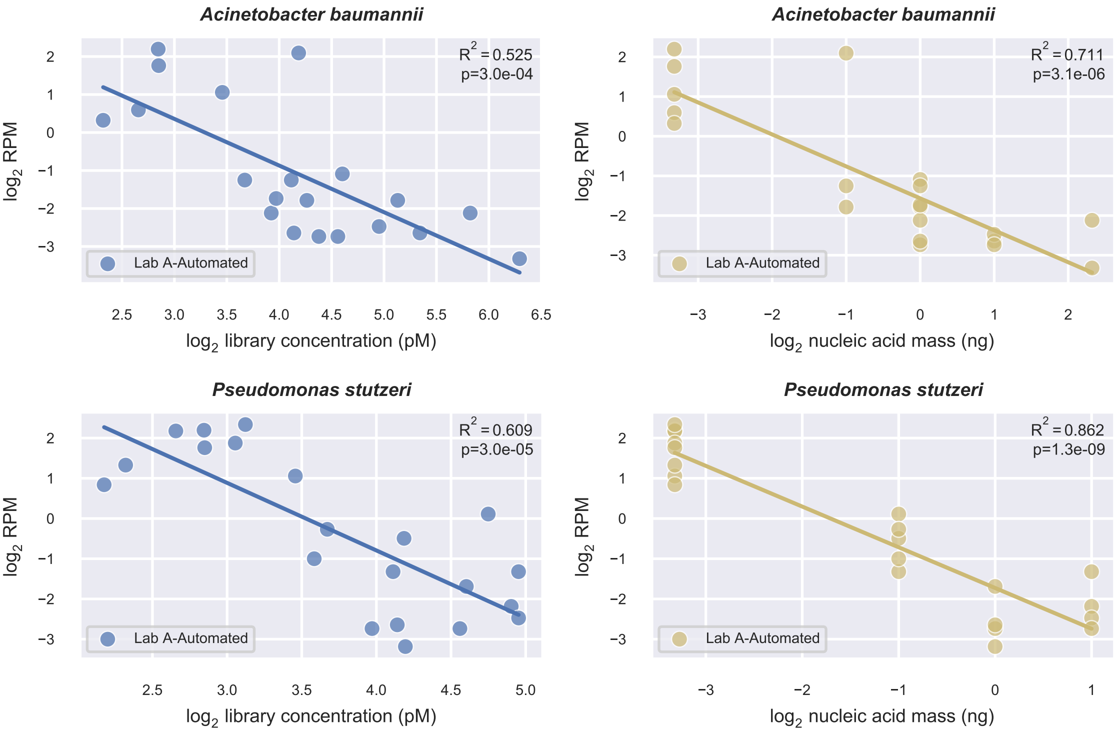
Acinetobacter baumannii
R² = 0.525
p=3.0e-04
Lab A-Automated
log₂ RPM
log₂ library concentration (pM)
Acinetobacter baumannii
R² = 0.711
p=3.1e-06
Lab A-Automated
log₂ RPM
log₂ nucleic acid mass (ng)
Pseudomonas stutzeri
R² = 0.609
p=3.0e-05
Lab A-Automated
log₂ RPM
log₂ library concentration (pM)
Pseudomonas stutzeri
R² = 0.862
p=1.3e-09
Lab A-Automated
log₂ RPM
log₂ nucleic acid mass (ng)

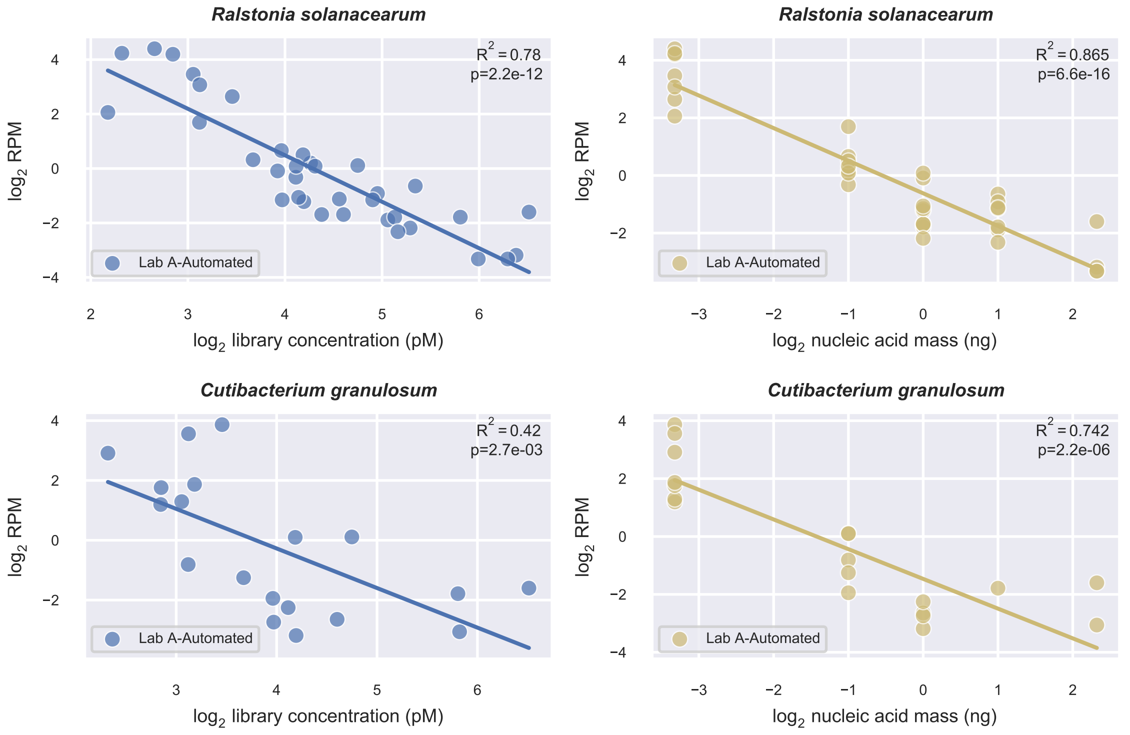

Ralstonia solanacearum
R² = 0.78
p=2.2e-12
Lab A-Automated
log₂ RPM
log₂ library concentration (pM)
Ralstonia solanacearum
R² = 0.865
p=6.6e-16
Lab A-Automated
log₂ RPM
log₂ nucleic acid mass (ng)
Cutibacterium granulosum
R² = 0.42
p=2.7e-03
Lab A-Automated
log₂ RPM
log₂ library concentration (pM)
Cutibacterium granulosum
R² = 0.742
p=2.2e-06
Lab A-Automated
log₂ RPM
log₂ nucleic acid mass (ng)

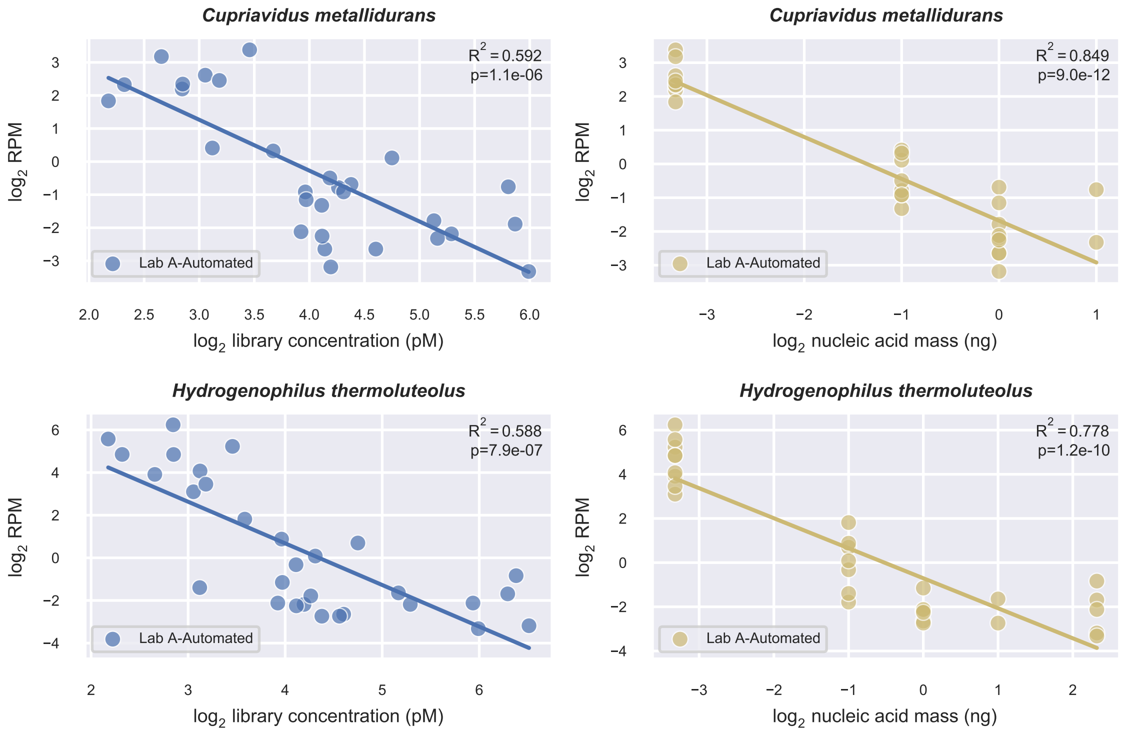

Cupriavidus metallidurans
R^2 = 0.592
p=1.1e-06
Lab A-Automated
log2 RPM
log2 library concentration (pM)
Cupriavidus metallidurans
R^2 = 0.849
p=9.0e-12
Lab A-Automated
log2 RPM
log2 nucleic acid mass (ng)
Hydrogenophilus thermoluteolus
R^2 = 0.588
p=7.9e-07
Lab A-Automated
log2 RPM
log2 library concentration (pM)
Hydrogenophilus thermoluteolus
R^2 = 0.778
p=1.2e-10
Lab A-Automated
log2 RPM
log2 nucleic acid mass (ng)

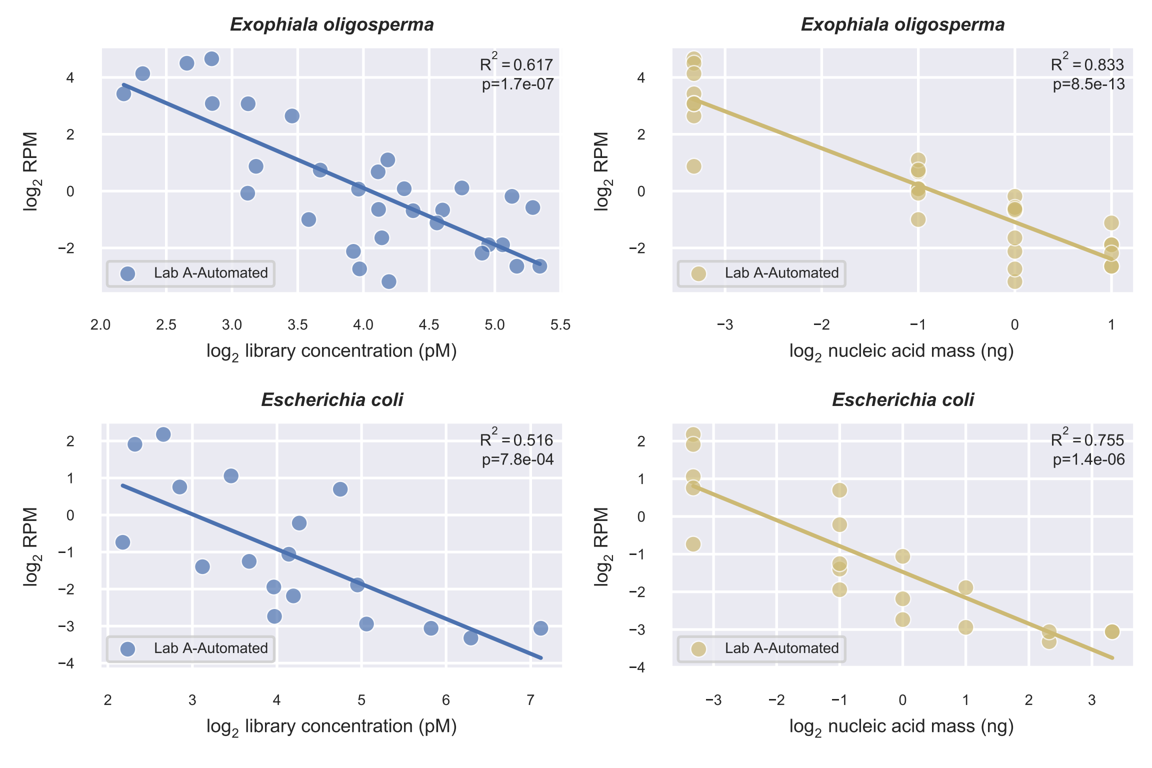
Exophiala oligosperma
$R^2 = 0.617$
p=1.7e-07
Lab A-Automated
log2 RPM
log2 library concentration (pM)
Exophiala oligosperma
$R^2 = 0.833$
p=8.5e-13
Lab A-Automated
log2 RPM
log2 nucleic acid mass (ng)
Escherichia coli
$R^2 = 0.516$
p=7.8e-04
Lab A-Automated
log2 RPM
log2 library concentration (pM)
Escherichia coli
$R^2 = 0.755$
p=1.4e-06
Lab A-Automated
log2 RPM
log2 nucleic acid mass (ng)

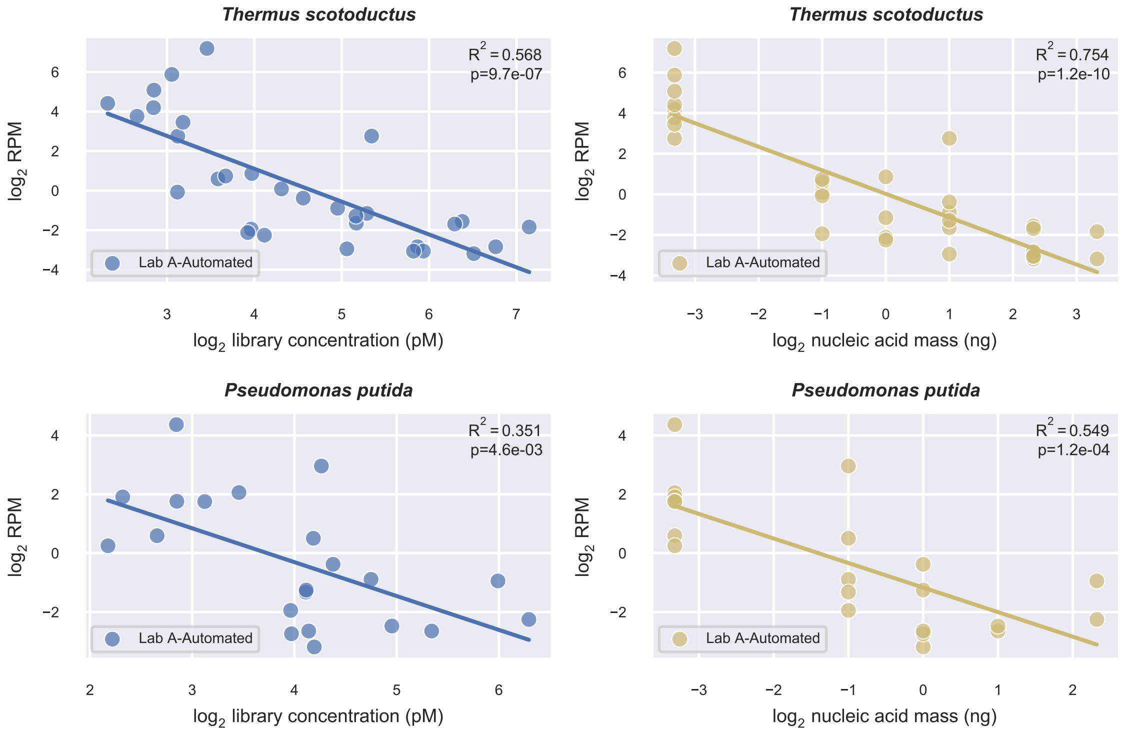

Thermus scotoductus
R² = 0.568
p=9.7e-07
Lab A-Automated
log2 RPM
log2 library concentration (pM)
Thermus scotoductus
R² = 0.754
p=1.2e-10
Lab A-Automated
log2 RPM
log2 nucleic acid mass (ng)
Pseudomonas putida
R² = 0.351
p=4.6e-03
Lab A-Automated
log2 RPM
log2 library concentration (pM)
Pseudomonas putida
R² = 0.549
p=1.2e-04
Lab A-Automated
log2 RPM
log2 nucleic acid mass (ng)

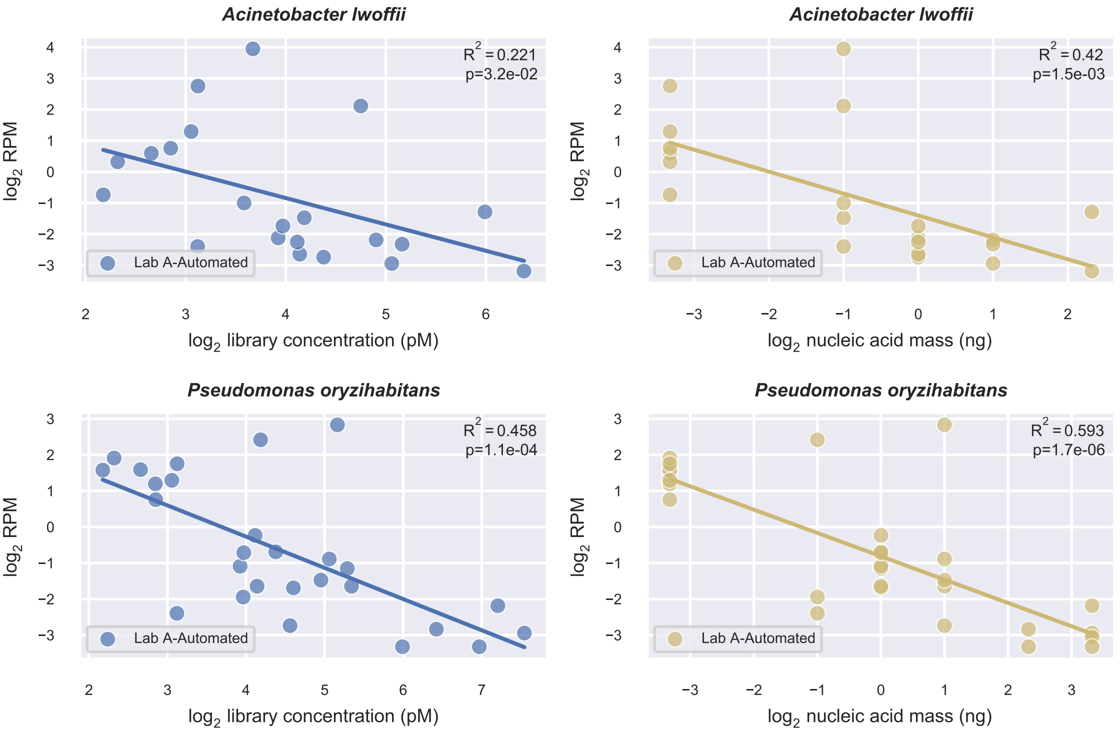

Acinetobacter lwoffii
R^2 = 0.221
p=3.2e-02
Lab A-Automated
log2 library concentration (pM)
log2 RPM
Acinetobacter lwoffii
R^2 = 0.42
p=1.5e-03
Lab A-Automated
log2 nucleic acid mass (ng)
log2 RPM
Pseudomonas oryzihabitans
R^2 = 0.458
p=1.1e-04
Lab A-Automated
log2 library concentration (pM)
log2 RPM
Pseudomonas oryzihabitans
R^2 = 0.593
p=1.7e-06
Lab A-Automated
log2 nucleic acid mass (ng)
log2 RPM

Supplemental Figure 2. Normality test of 38 background contaminant species identified in Figure 1 model (Lab A, automated protocol for library preparation), related to Figure 1.

Histograms of the residual for each observation informing the linear regression between log_2-transformed RPM and log_2-transformed library concentration of each taxon. Except for *Ralstonia pickettii, Ralstonia solanacearum, Sphingomonas paucimobilis, Pseudomonas oryzihabitans, Acinetobacter lwoffii* and *Methylobacterium aquaticum,* the residuals of the model fits for other taxa were normally distributed. The p value of the normality test is labeled in the top right corner of each panel.

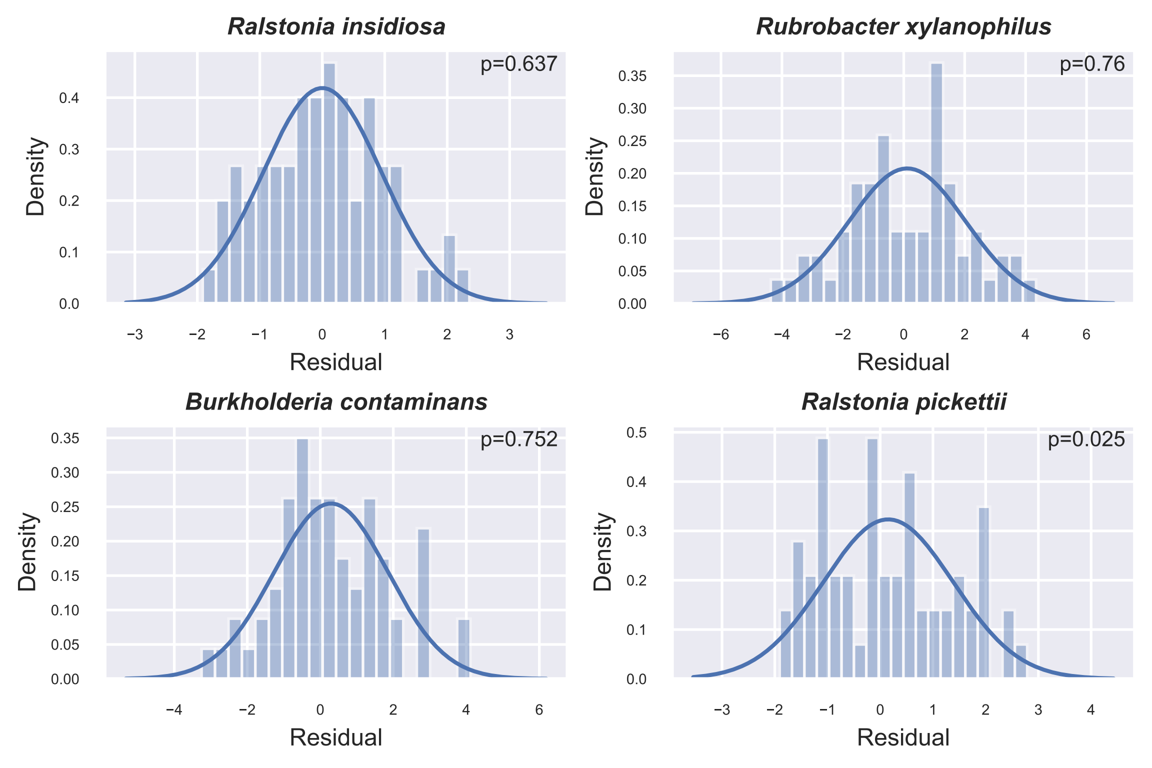

Ralstonia insidiosa
p=0.637
Density
Residual
Rubrobacter xylanophilus
p=0.76
Density
Residual
Burkholderia contaminans
p=0.752
Density
Residual
Ralstonia pickettii
p=0.025
Density
Residual

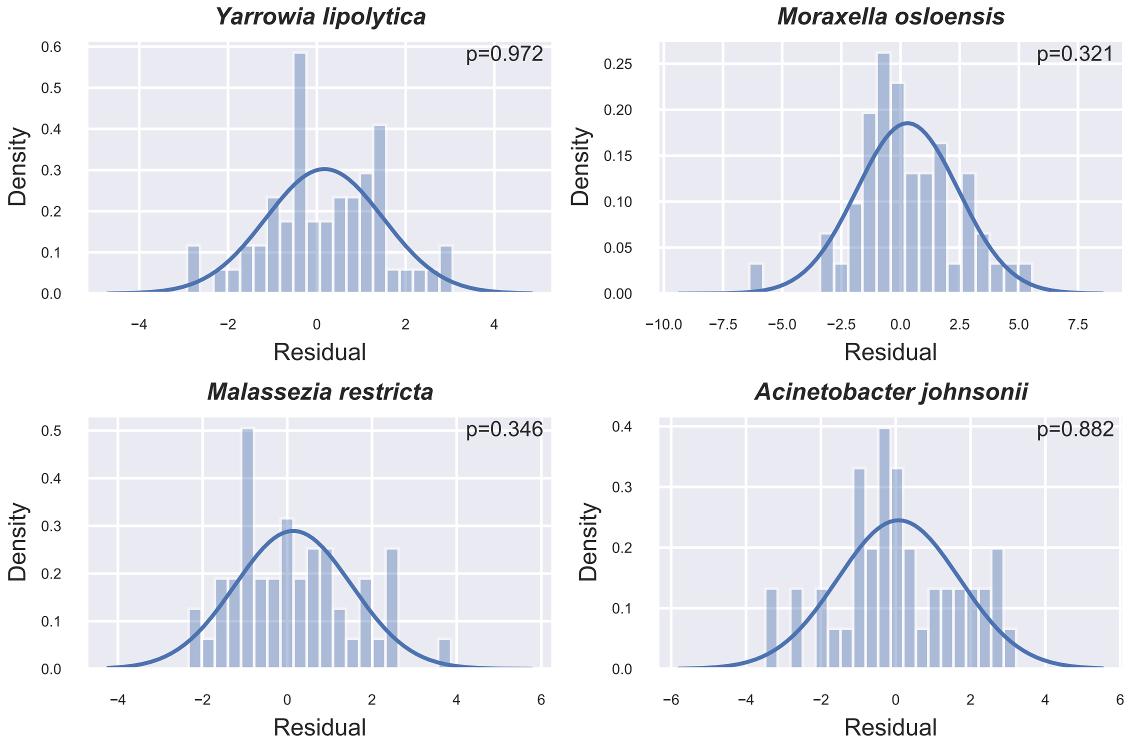
Yarrowia lipolytica
p=0.972
Density
Residual
Moraxella osloensis
p=0.321
Density
Residual
Malassezia restricta
p=0.346
Density
Residual
Acinetobacter johnsonii
p=0.882
Density
Residual

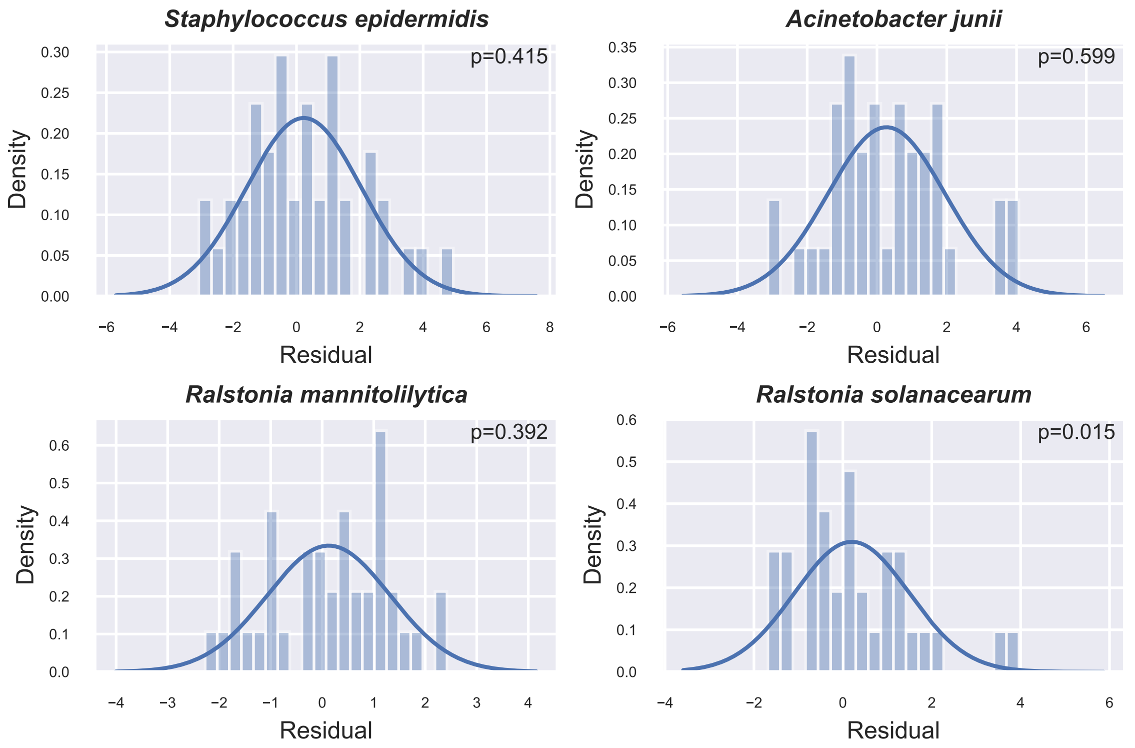

Staphylococcus epidermidis
p=0.415
Acinetobacter junii
p=0.599
Ralstonia mannitolilytica
p=0.392
Ralstonia solanacearum
p=0.015
Density
Residual

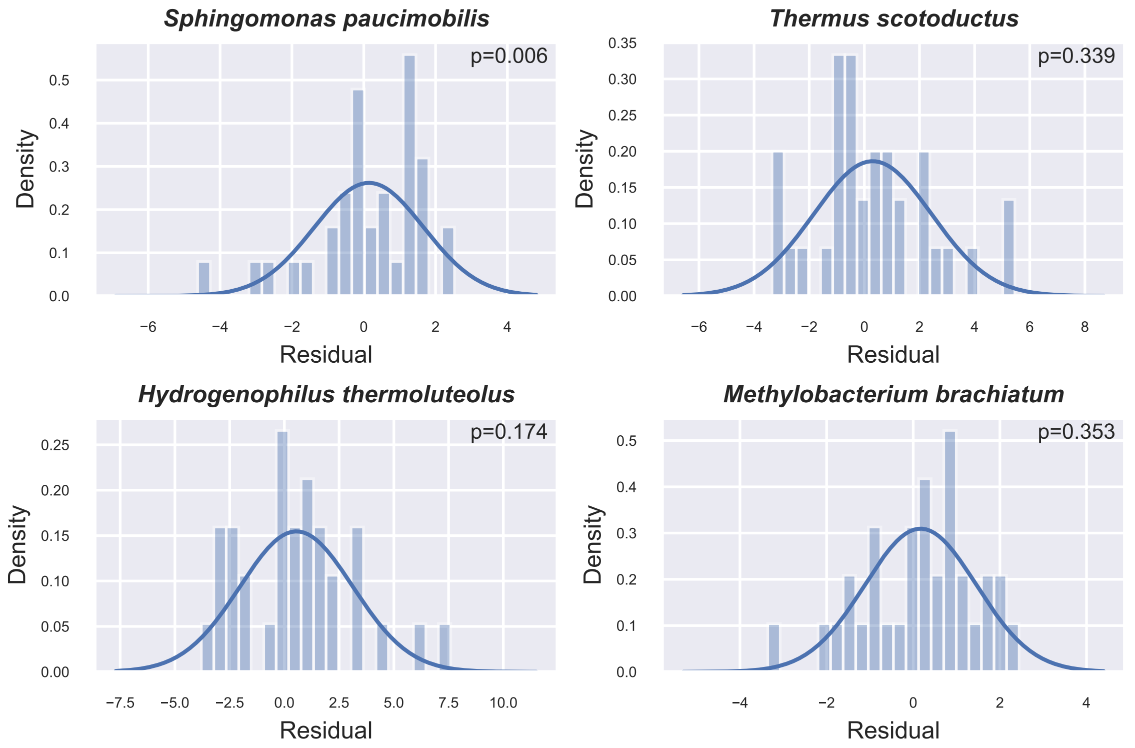
Sphingomonas paucimobilis
p=0.006
Density
Residual
Thermus scotoductus
p=0.339
Density
Residual
Hydrogenophilus thermoluteolus
p=0.174
Density
Residual
Methylobacterium brachiatum
p=0.353
Density
Residual

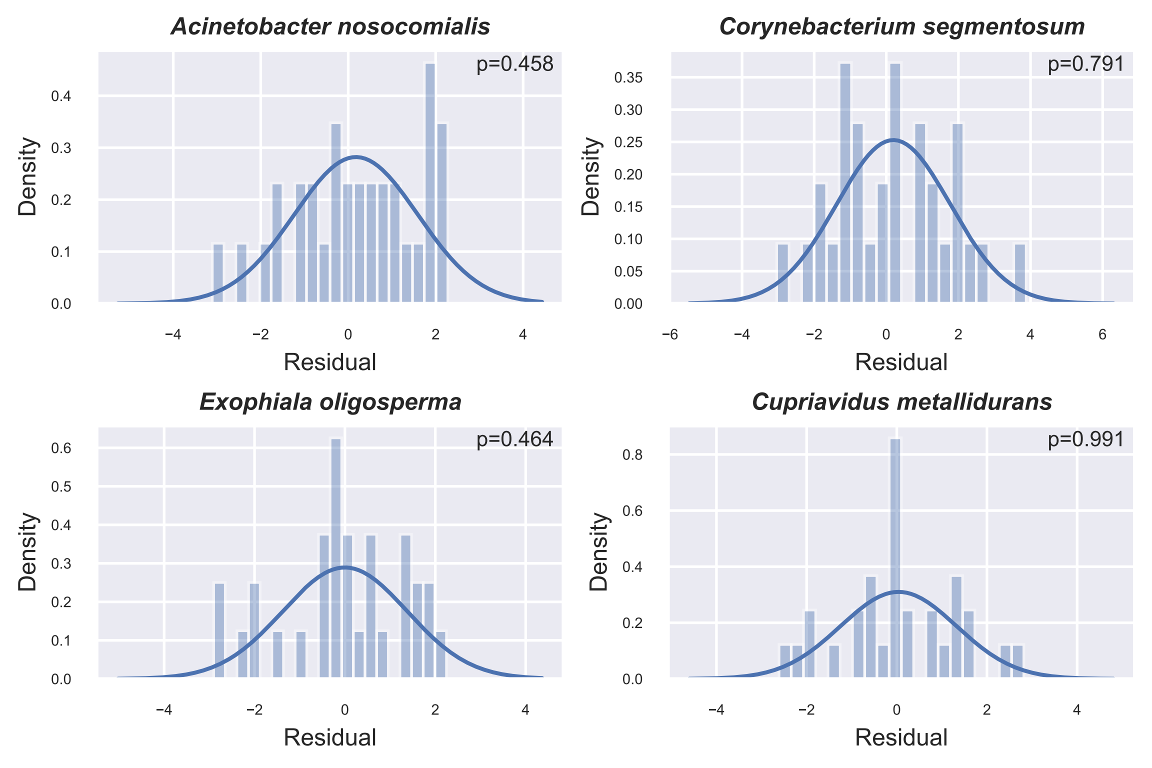

Acinetobacter nosocomialis
p=0.458
Density
Residual
Corynebacterium segmentosum
p=0.791
Density
Residual
Exophiala oligosperma
p=0.464
Density
Residual
Cupriavidus metallidurans
p=0.991
Density
Residual

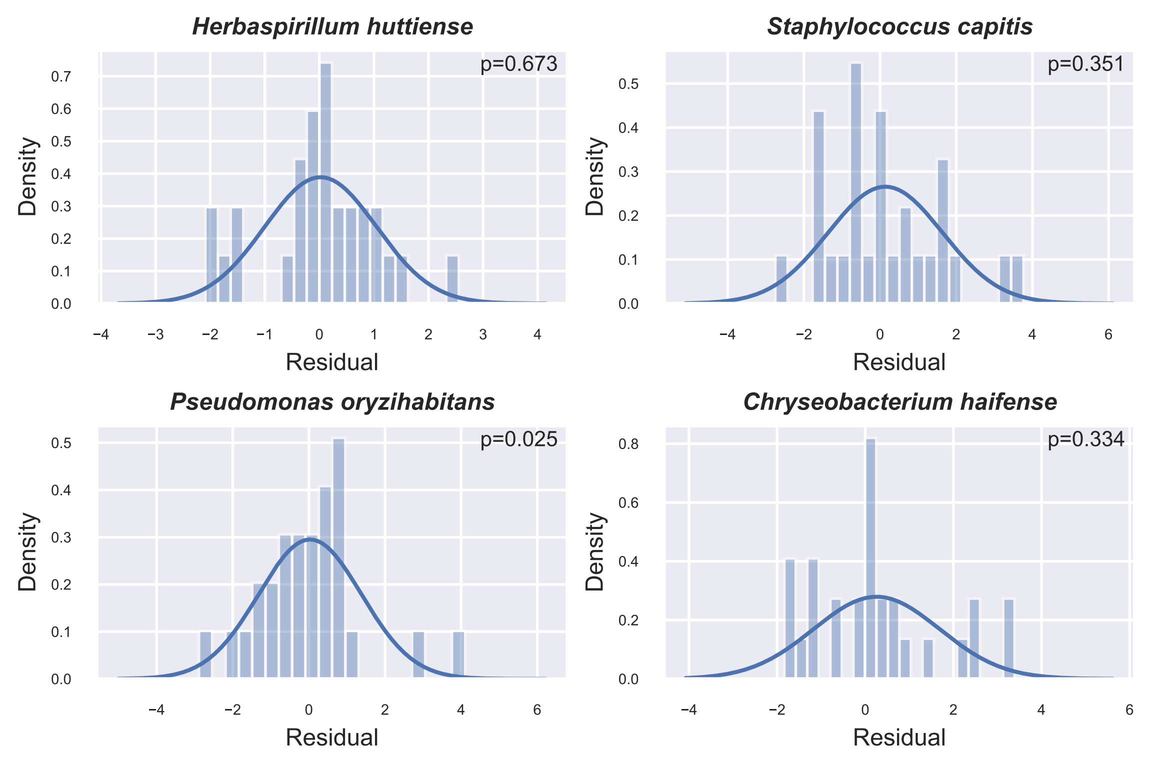
Herbaspirillum huttiense
p=0.673
Staphylococcus capitis
p=0.351
Pseudomonas oryzihabitans
p=0.025
Chryseobacterium haifense
p=0.334
Density
Residual

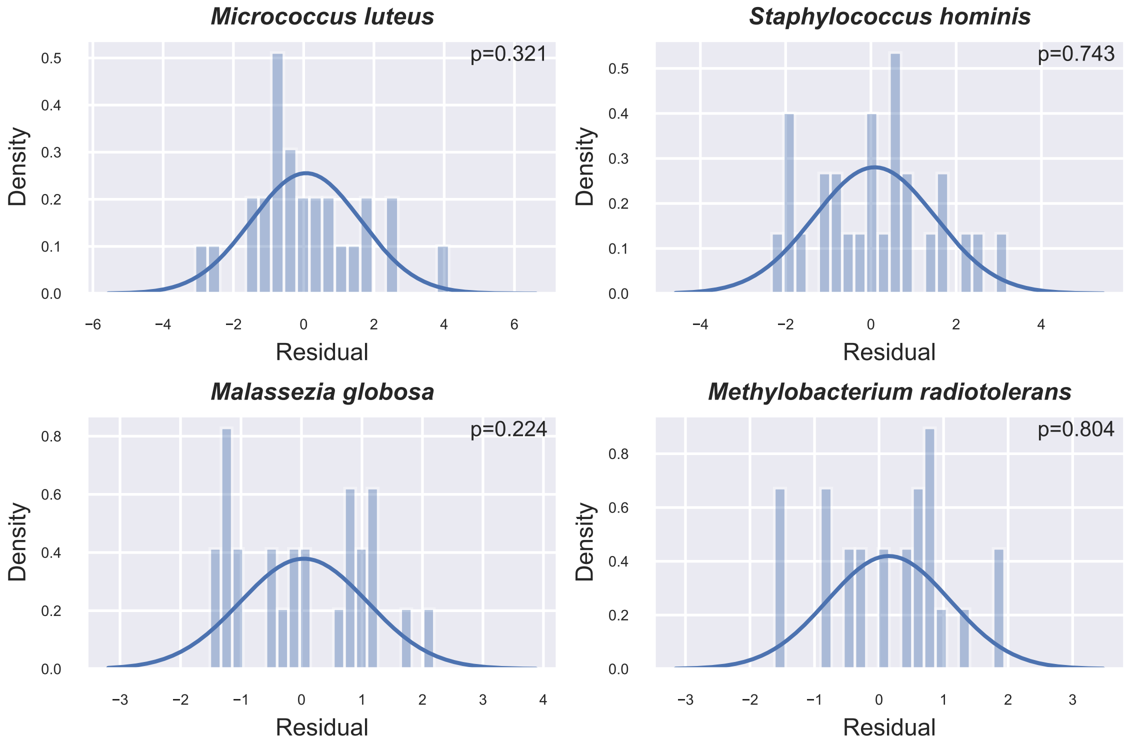
Micrococcus luteus
p=0.321
Density
Residual
Staphylococcus hominis
p=0.743
Density
Residual
Malassezia globosa
p=0.224
Density
Residual
Methylobacterium radiotolerans
p=0.804
Density
Residual

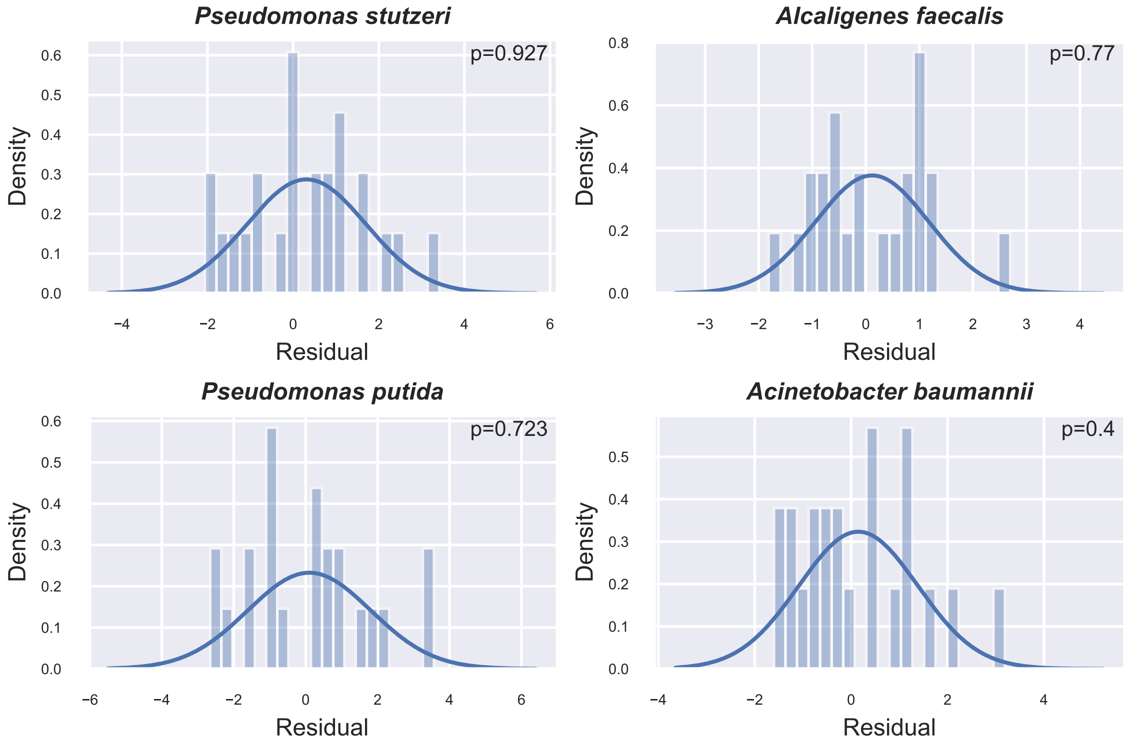

Pseudomonas stutzeri
p=0.927
Alcaligenes faecalis
p=0.77
Pseudomonas putida
p=0.723
Acinetobacter baumannii
p=0.4
Density
Residual

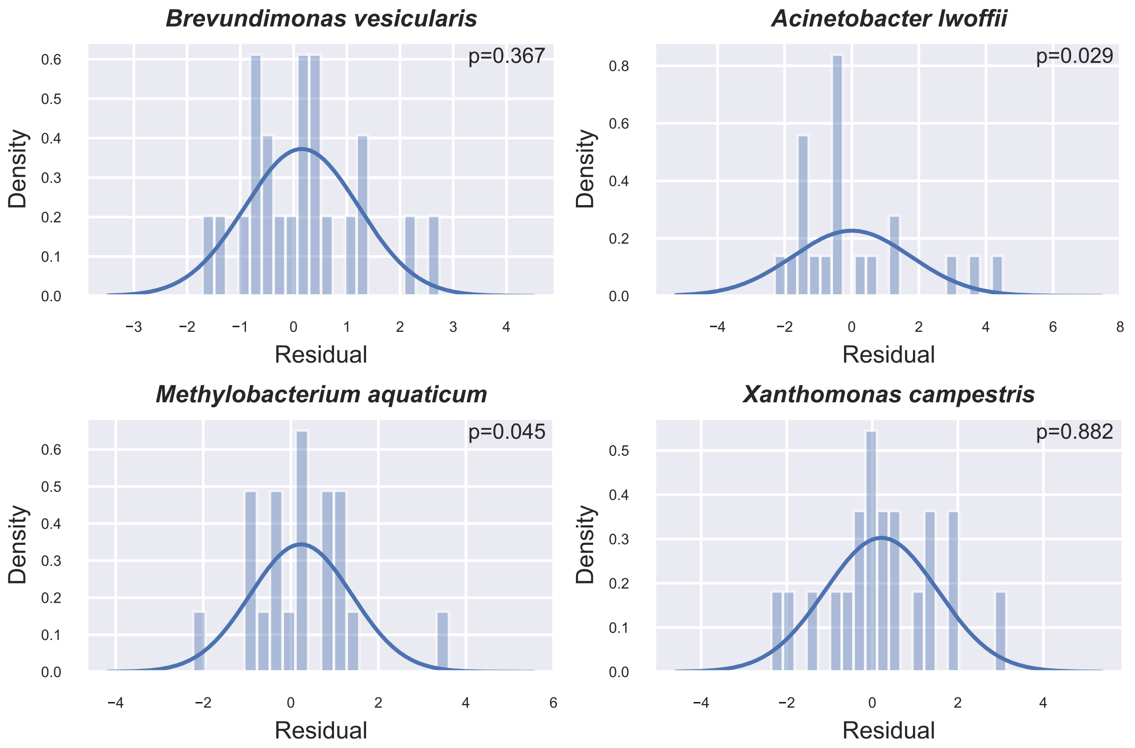

Brevundimonas vesicularis
p=0.367
Density
Residual
Acinetobacter lwoffii
p=0.029
Density
Residual
Methylobacterium aquaticum
p=0.045
Density
Residual
Xanthomonas campestris
p=0.882
Density
Residual

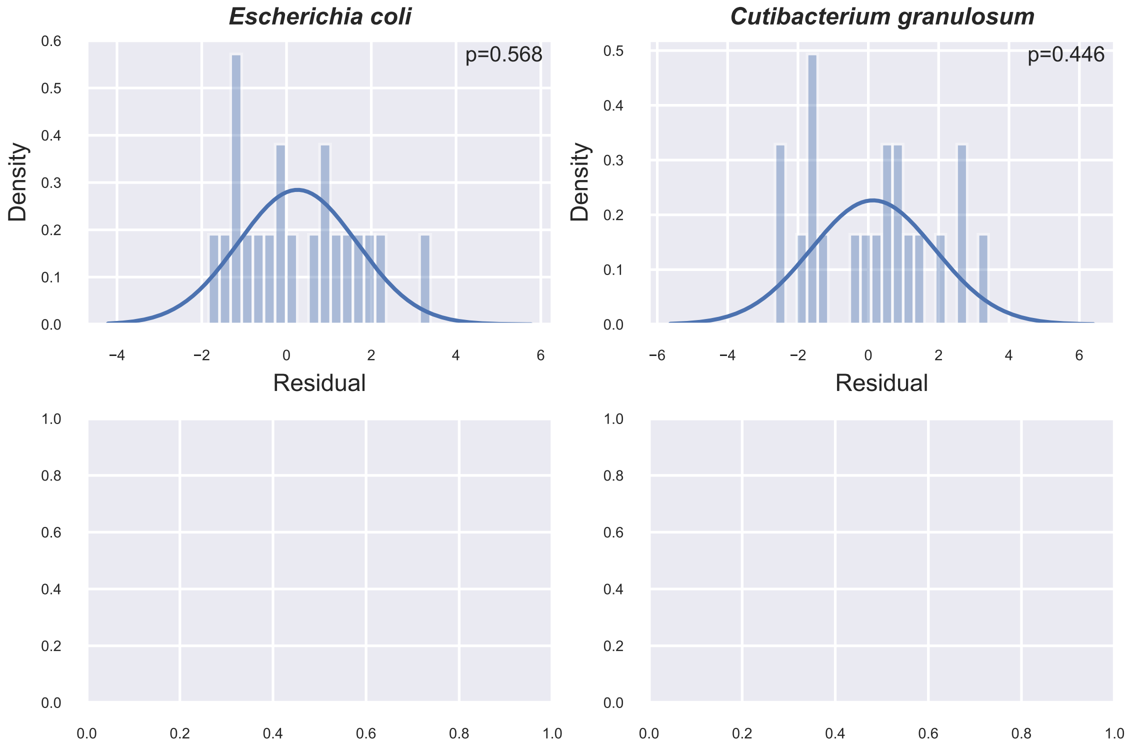

Escherichia coli
p=0.568
Density
Residual
Cutibacterium granulosum
p=0.446
Density
Residual

Supplemental Figure 3. Genome coverage maps of 38 background contaminant species identified in Figure 1 model (Lab A, automated protocol for library preparation), related to Figure 1.

To exclude nucleic acid contamination originate from aerosolized PCR amplicons, the genome distribution of reads for each taxon was also investigated.

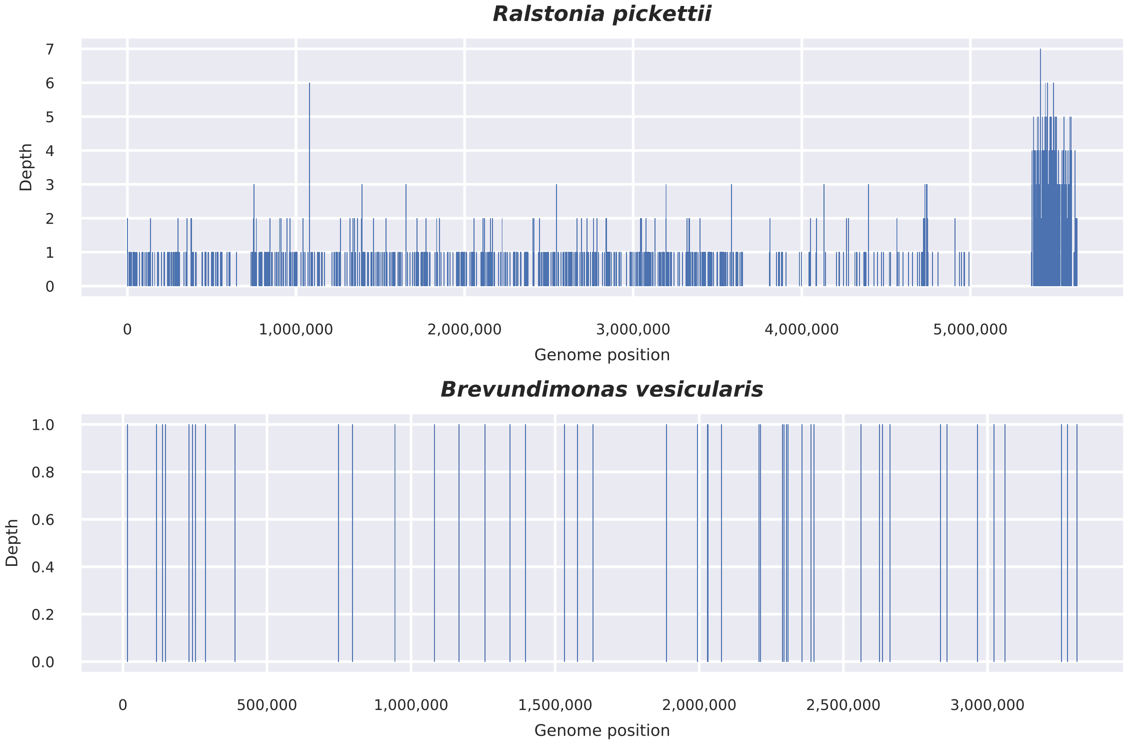

Ralstonia pickettii
Depth
0
1
2
3
4
5
6
7
0
1,000,000
2,000,000
3,000,000
4,000,000
5,000,000
Genome position
Brevundimonas vesicularis
Depth
0.0
0.2
0.4
0.6
0.8
1.0
0
500,000
1,000,000
1,500,000
2,000,000
2,500,000
3,000,000
Genome position

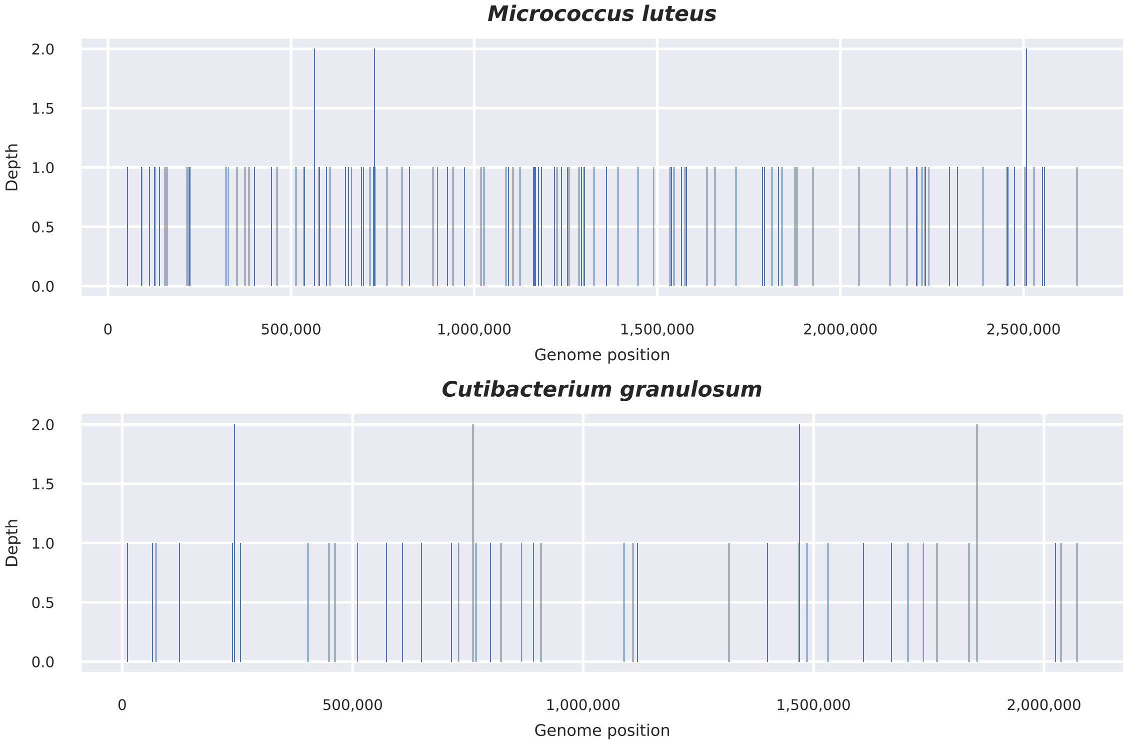

Micrococcus luteus
Depth
0.0
0.5
1.0
1.5
2.0
0
500,000
1,000,000
1,500,000
2,000,000
2,500,000
Genome position
Cutibacterium granulosum
Depth
0.0
0.5
1.0
1.5
2.0
0
500,000
1,000,000
1,500,000
2,000,000
Genome position

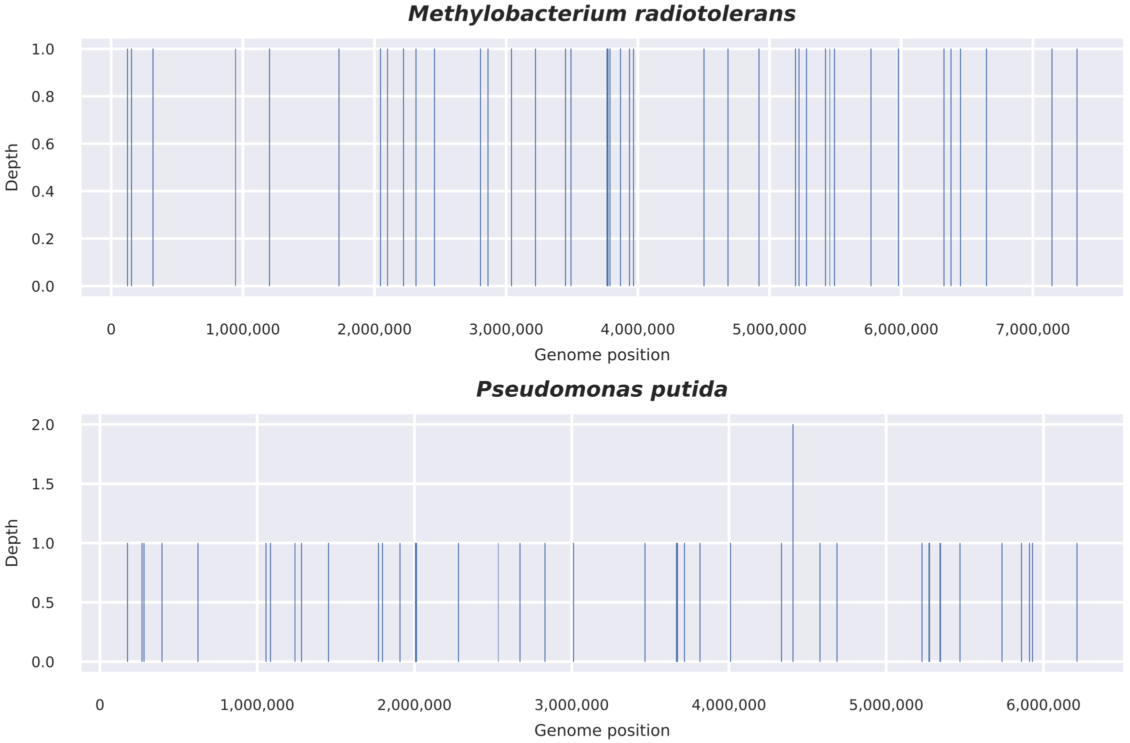
Methylobacterium radiotolerans
Depth
1.0
0.8
0.6
0.4
0.2
0.0
0
1,000,000
2,000,000
3,000,000
4,000,000
5,000,000
6,000,000
7,000,000
Genome position
Pseudomonas putida
Depth
2.0
1.5
1.0
0.5
0.0
0
1,000,000
2,000,000
3,000,000
4,000,000
5,000,000
6,000,000
Genome position

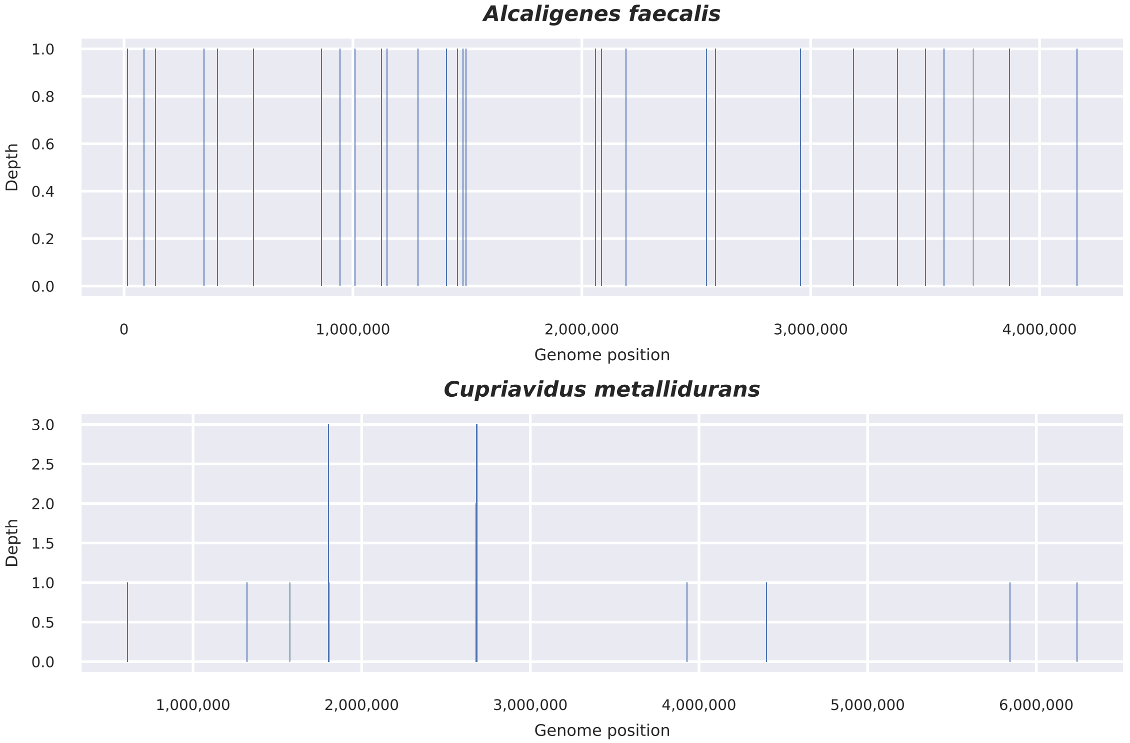
Alcaligenes faecalis
Depth
1.0
0.8
0.6
0.4
0.2
0.0
0
1,000,000
2,000,000
3,000,000
4,000,000
Genome position
Cupriavidus metallidurans
Depth
3.0
2.5
2.0
1.5
1.0
0.5
0.0
1,000,000
2,000,000
3,000,000
4,000,000
5,000,000
6,000,000
Genome position

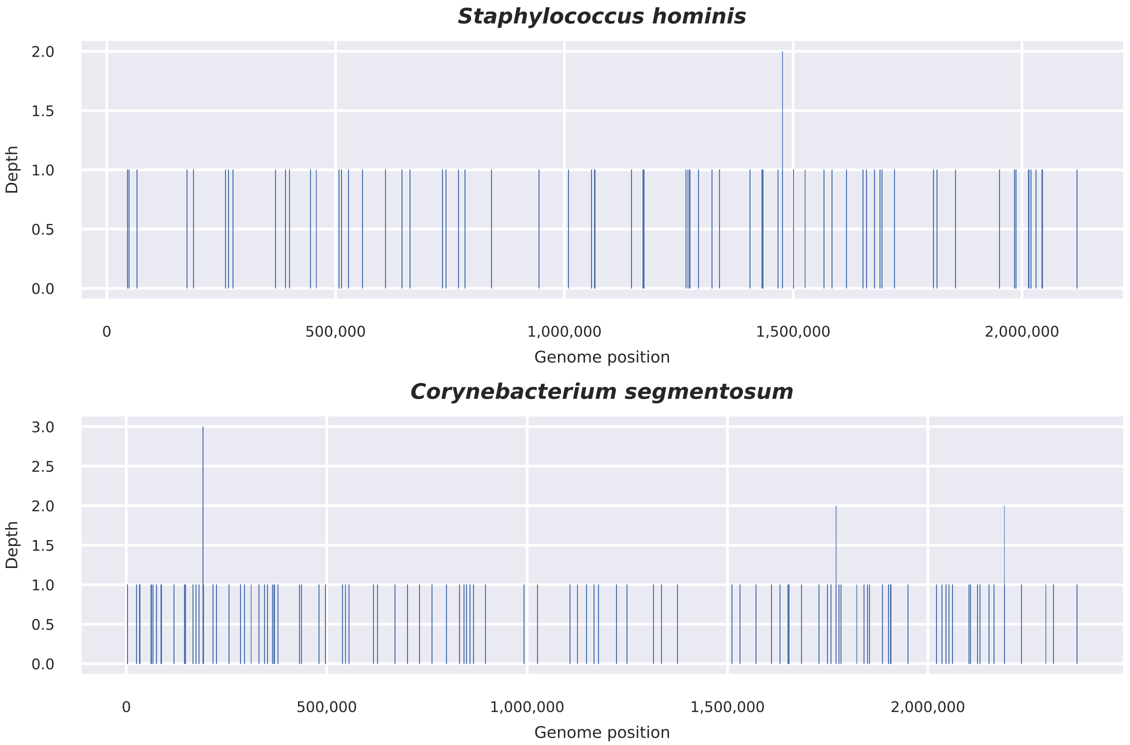
Staphylococcus hominis
Depth
2.0
1.5
1.0
0.5
0.0
0
500,000
1,000,000
1,500,000
2,000,000
Genome position
Corynebacterium segmentosum
Depth
3.0
2.5
2.0
1.5
1.0
0.5
0.0
0
500,000
1,000,000
1,500,000
2,000,000
Genome position

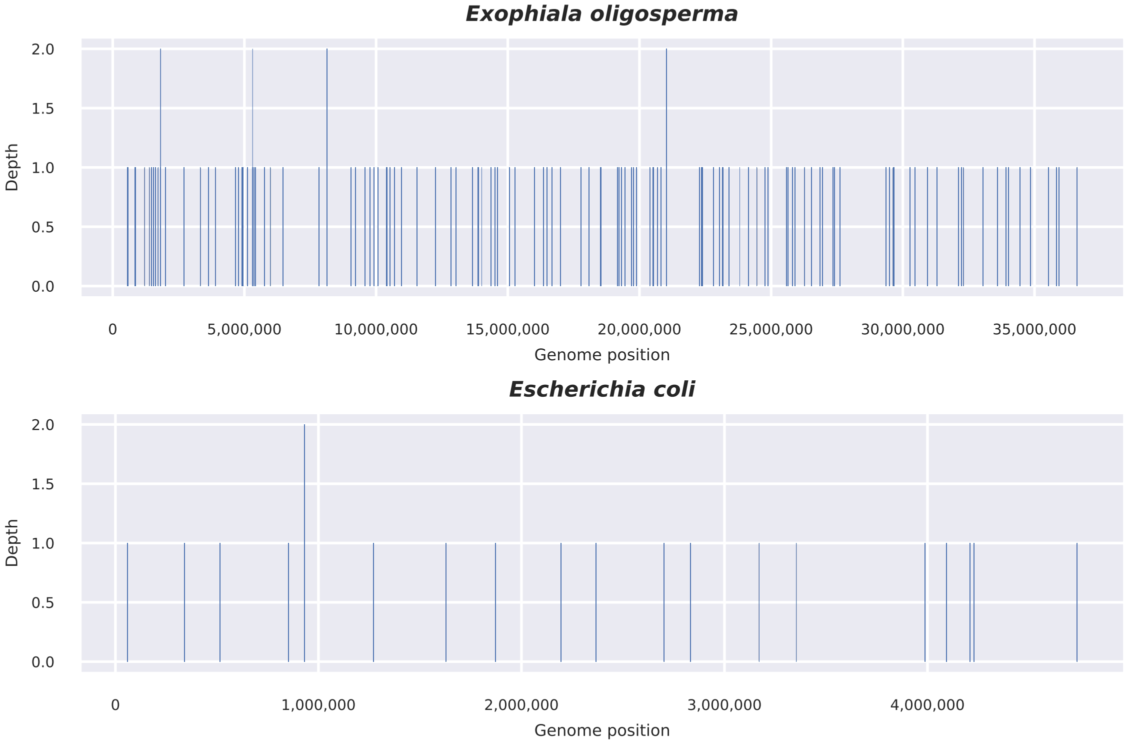

Exophiala oligosperma
Depth
2.0
1.5
1.0
0.5
0.0
0
5,000,000
10,000,000
15,000,000
20,000,000
25,000,000
30,000,000
35,000,000
Genome position
Escherichia coli
Depth
2.0
1.5
1.0
0.5
0.0
0
1,000,000
2,000,000
3,000,000
4,000,000
Genome position

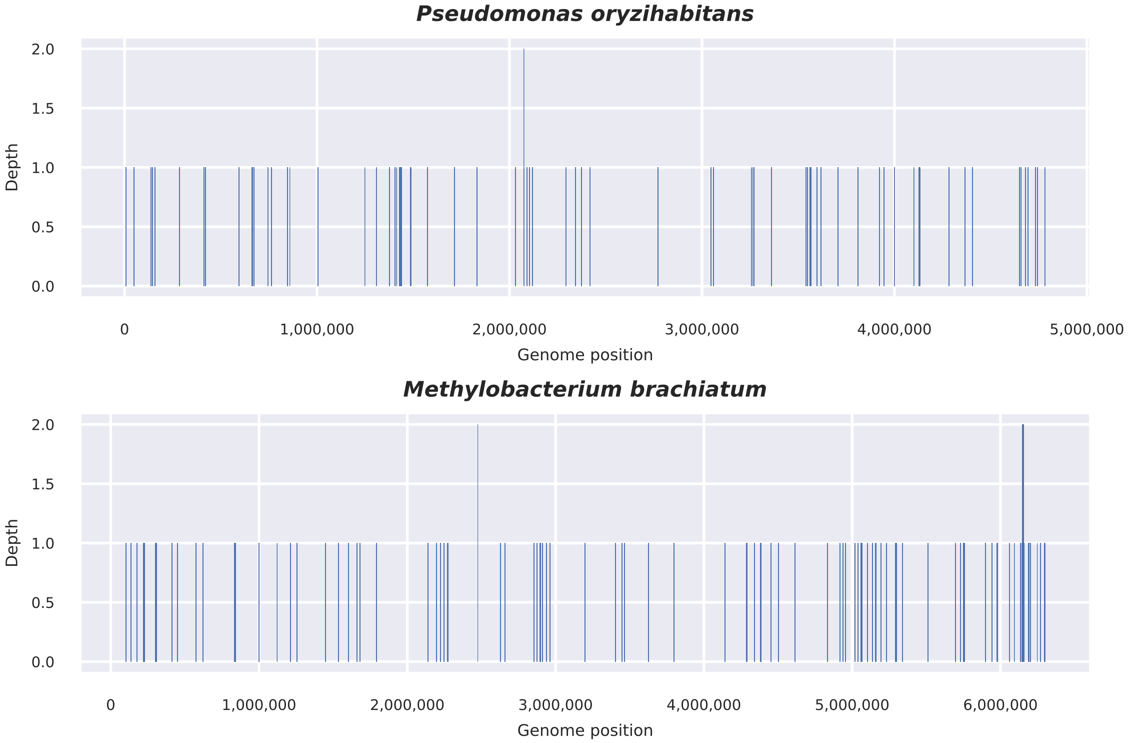

Pseudomonas oryzihabitans
Depth
2.0
1.5
1.0
0.5
0.0
0
1,000,000
2,000,000
3,000,000
4,000,000
5,000,000
Genome position
Methylobacterium brachiatum
Depth
2.0
1.5
1.0
0.5
0.0
0
1,000,000
2,000,000
3,000,000
4,000,000
5,000,000
6,000,000
Genome position

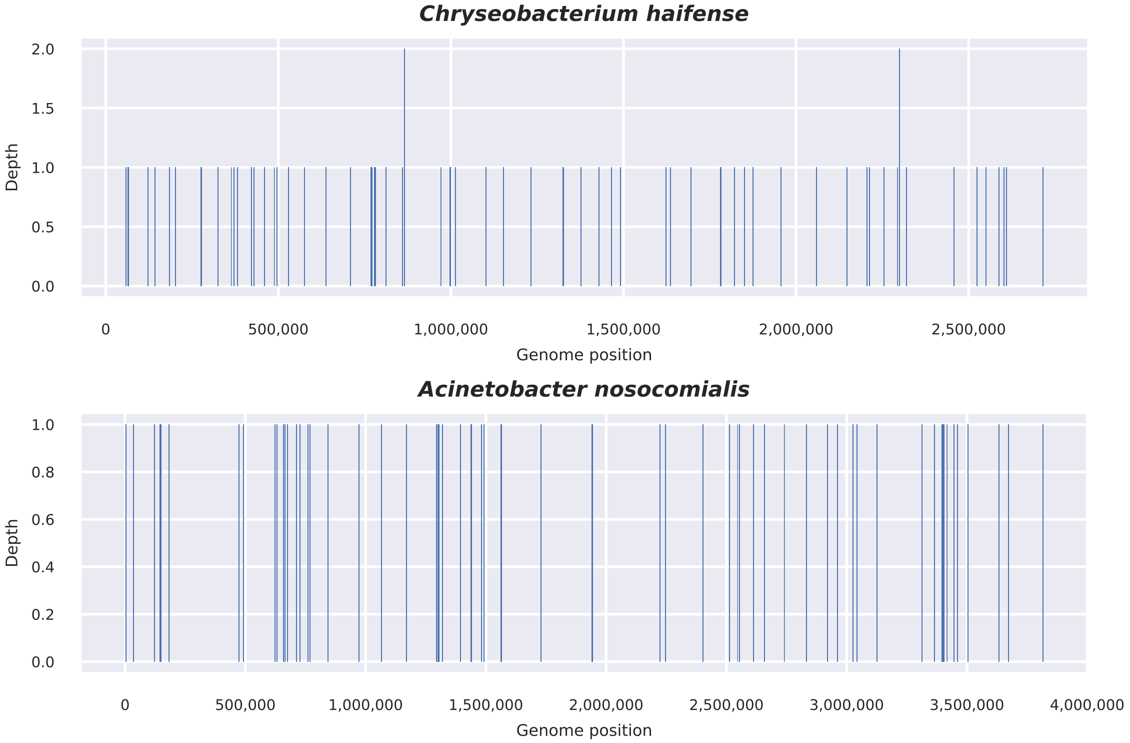

Chryseobacterium haifense
Depth
2.0
1.5
1.0
0.5
0.0
0
500,000
1,000,000
1,500,000
2,000,000
2,500,000
Genome position
Acinetobacter nosocomialis
Depth
1.0
0.8
0.6
0.4
0.2
0.0
0
500,000
1,000,000
1,500,000
2,000,000
2,500,000
3,000,000
3,500,000
4,000,000
Genome position

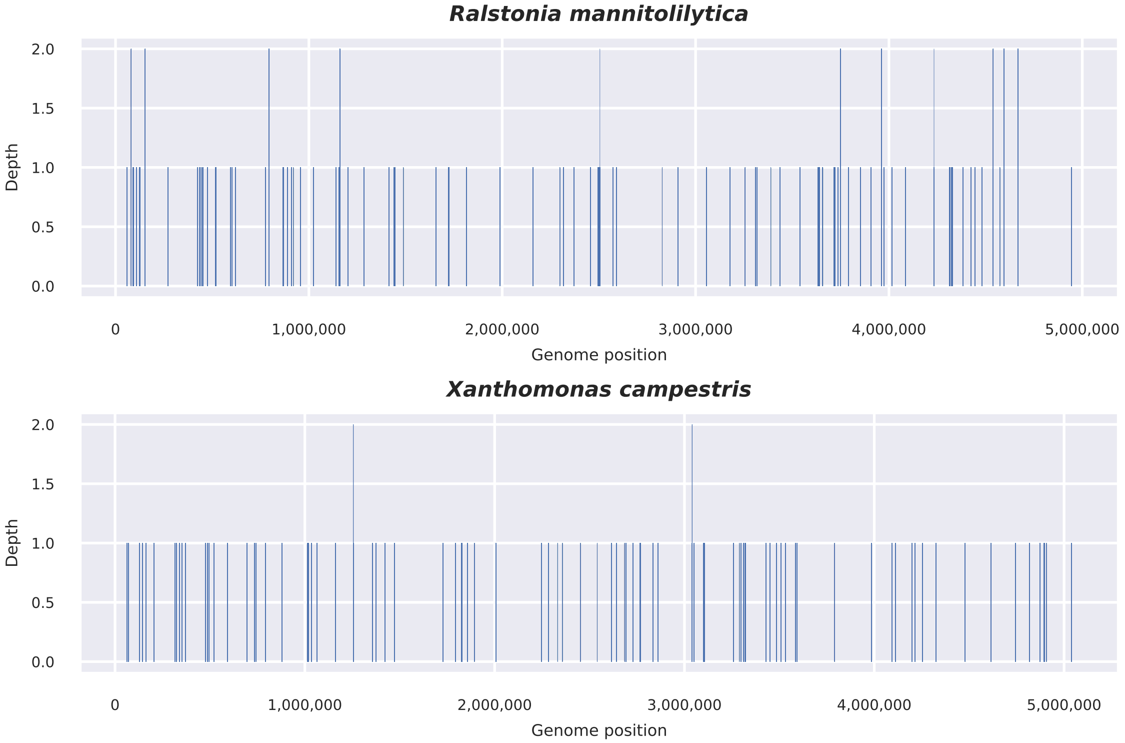

Ralstonia mannitolilytica
Depth
2.0
1.5
1.0
0.5
0.0
0
1,000,000
2,000,000
3,000,000
4,000,000
5,000,000
Genome position
Xanthomonas campestris
Depth
2.0
1.5
1.0
0.5
0.0
0
1,000,000
2,000,000
3,000,000
4,000,000
5,000,000
Genome position

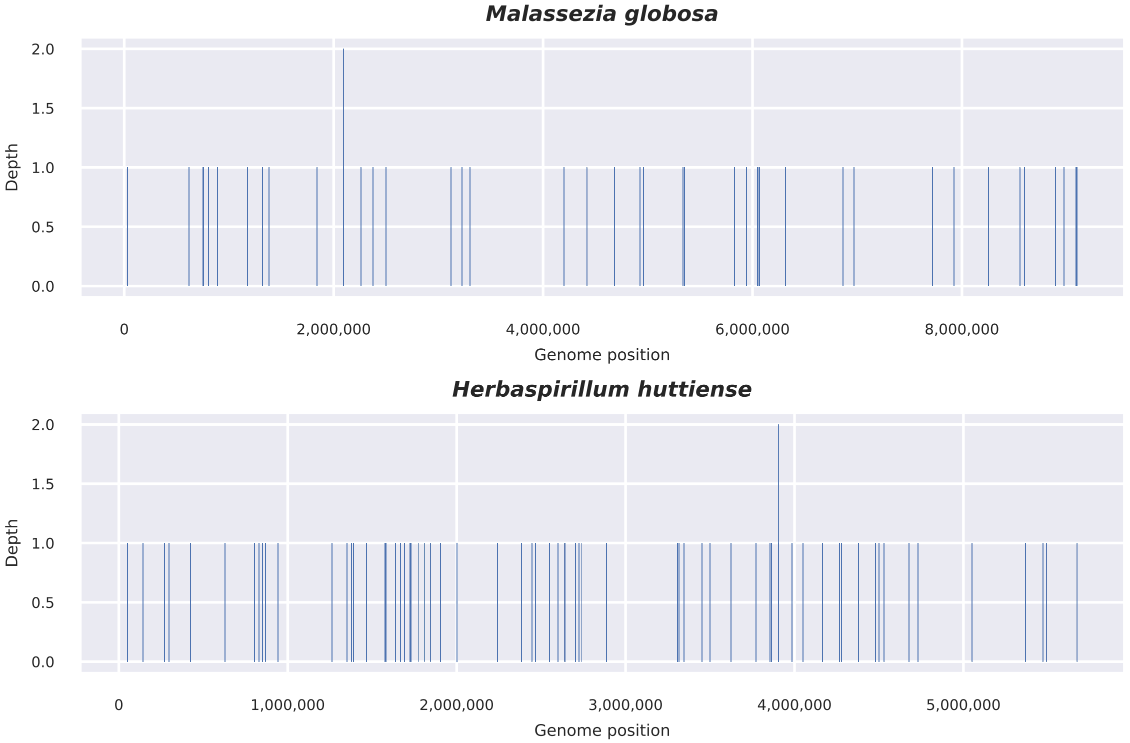
Malassezia globosa
Depth
2.0
1.5
1.0
0.5
0.0
0
2,000,000
4,000,000
6,000,000
8,000,000
Genome position
Herbaspirillum huttiense
Depth
2.0
1.5
1.0
0.5
0.0
0
1,000,000
2,000,000
3,000,000
4,000,000
5,000,000
Genome position

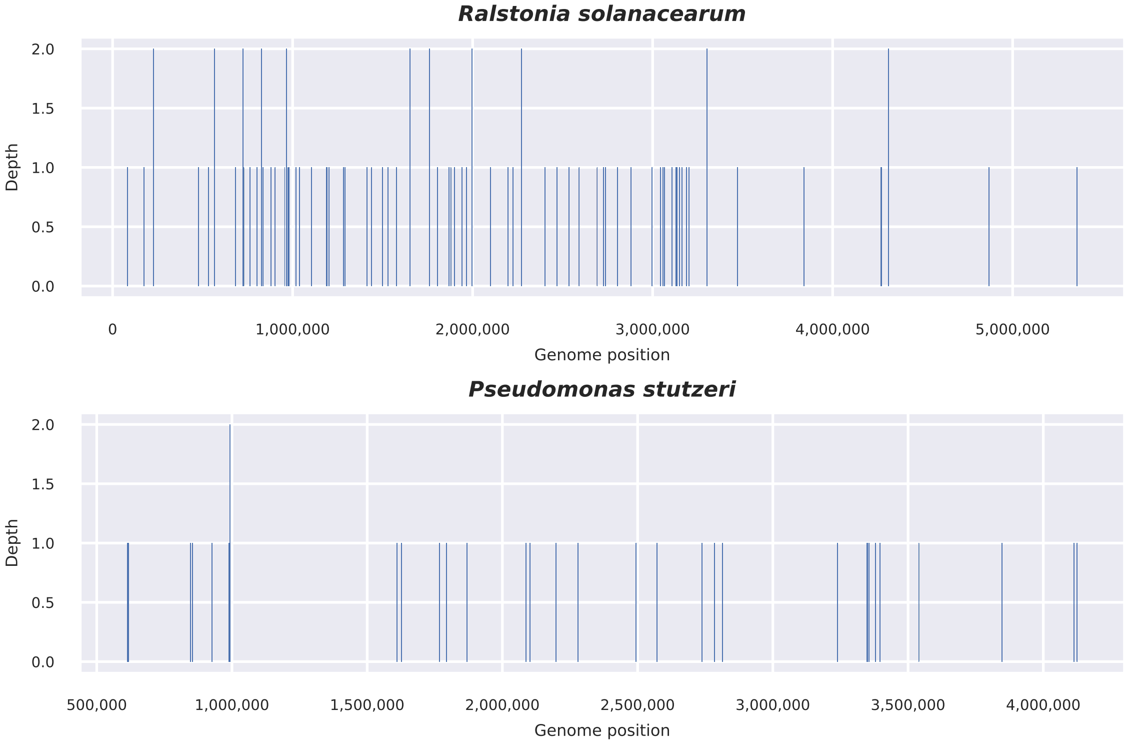

Ralstonia solanacearum
Depth
2.0
1.5
1.0
0.5
0.0
0
1,000,000
2,000,000
3,000,000
4,000,000
5,000,000
Genome position
Pseudomonas stutzeri
Depth
2.0
1.5
1.0
0.5
0.0
500,000
1,000,000
1,500,000
2,000,000
2,500,000
3,000,000
3,500,000
4,000,000
Genome position

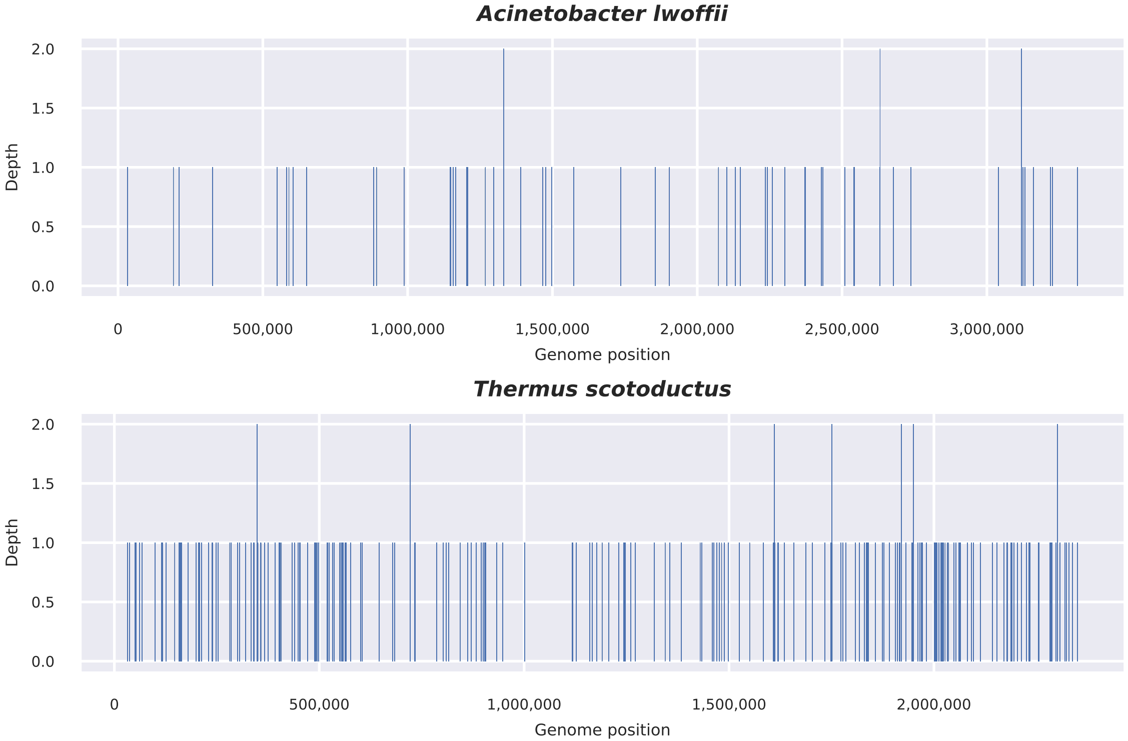

Acinetobacter lwoffii
Depth
2.0
1.5
1.0
0.5
0.0
0
500,000
1,000,000
1,500,000
2,000,000
2,500,000
3,000,000
Genome position
Thermus scotoductus
Depth
2.0
1.5
1.0
0.5
0.0
0
500,000
1,000,000
1,500,000
2,000,000
Genome position

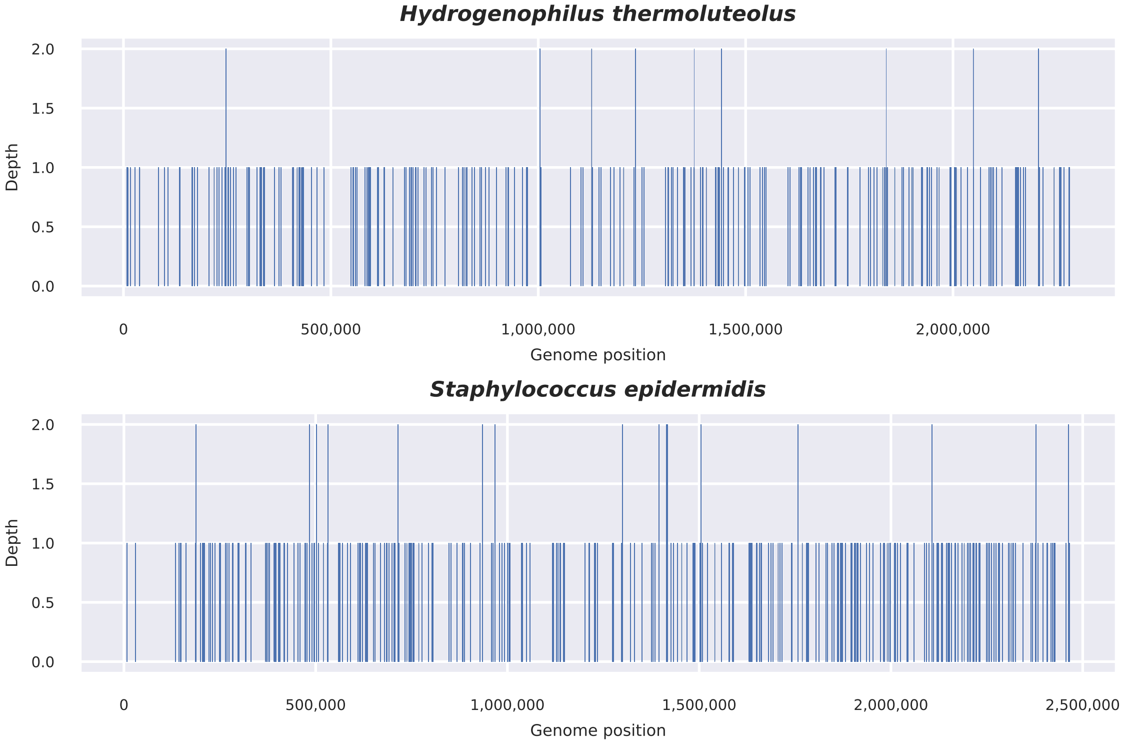
Hydrogenophilus thermoluteolus
Depth
2.0
1.5
1.0
0.5
0.0
0
500,000
1,000,000
1,500,000
2,000,000
Genome position
Staphylococcus epidermidis
Depth
2.0
1.5
1.0
0.5
0.0
0
500,000
1,000,000
1,500,000
2,000,000
2,500,000
Genome position

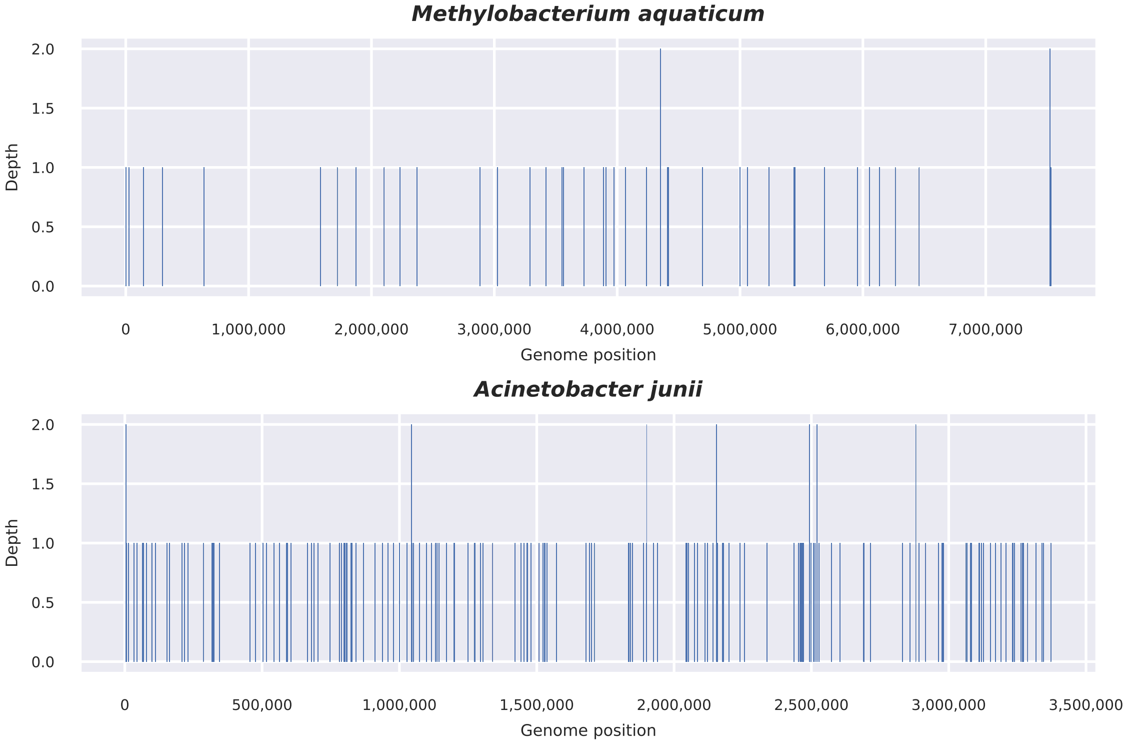

Methylobacterium aquaticum
Depth
2.0
1.5
1.0
0.5
0.0
0
1,000,000
2,000,000
3,000,000
4,000,000
5,000,000
6,000,000
7,000,000
Genome position
Acinetobacter junii
Depth
2.0
1.5
1.0
0.5
0.0
0
500,000
1,000,000
1,500,000
2,000,000
2,500,000
3,000,000
3,500,000
Genome position

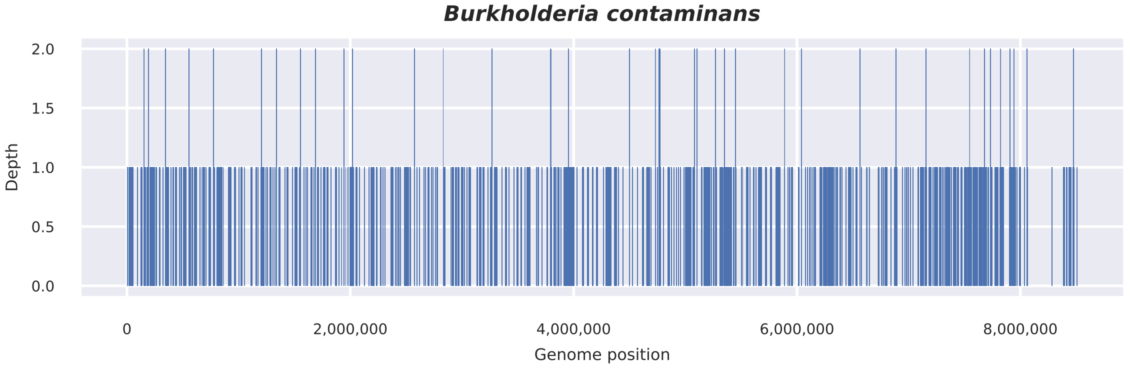

Burkholderia contaminans
Depth
2.0
1.5
1.0
0.5
0.0
0
2,000,000
4,000,000
6,000,000
8,000,000
Genome position

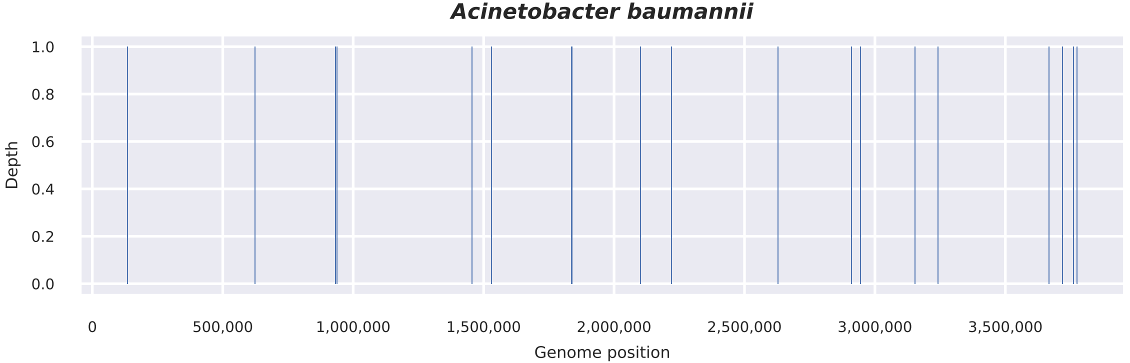

Acinetobacter baumannii
Depth
1.0
0.8
0.6
0.4
0.2
0.0
0
500,000
1,000,000
1,500,000
2,000,000
2,500,000
3,000,000
3,500,000
Genome position

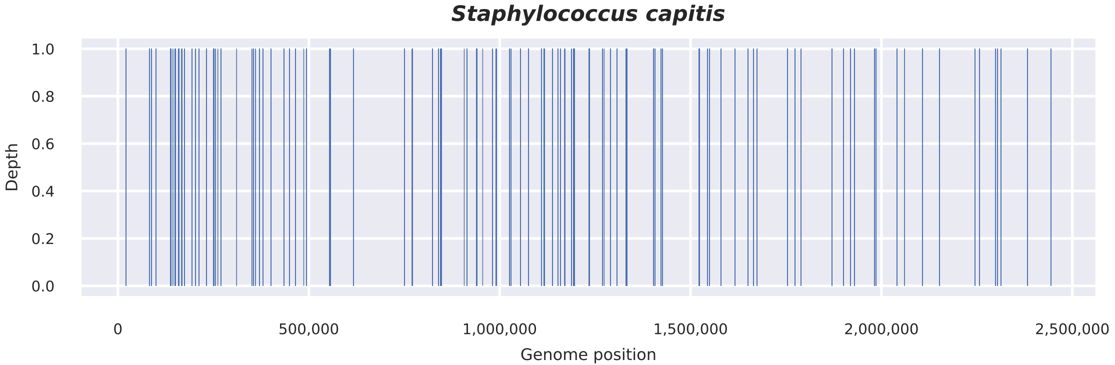

Staphylococcus capitis
Depth
1.0
0.8
0.6
0.4
0.2
0.0
0
500,000
1,000,000
1,500,000
2,000,000
2,500,000
Genome position

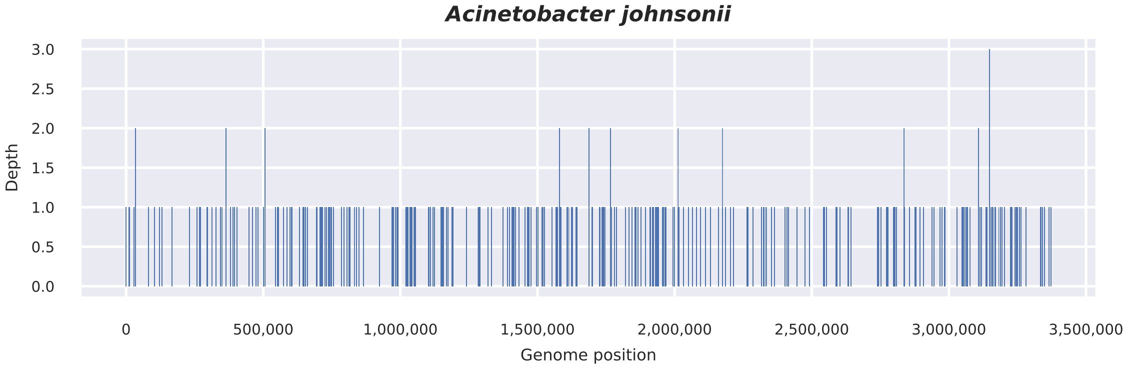

Acinetobacter johnsonii
Depth
3.0
2.5
2.0
1.5
1.0
0.5
0.0
0
500,000
1,000,000
1,500,000
2,000,000
2,500,000
3,000,000
3,500,000
Genome position

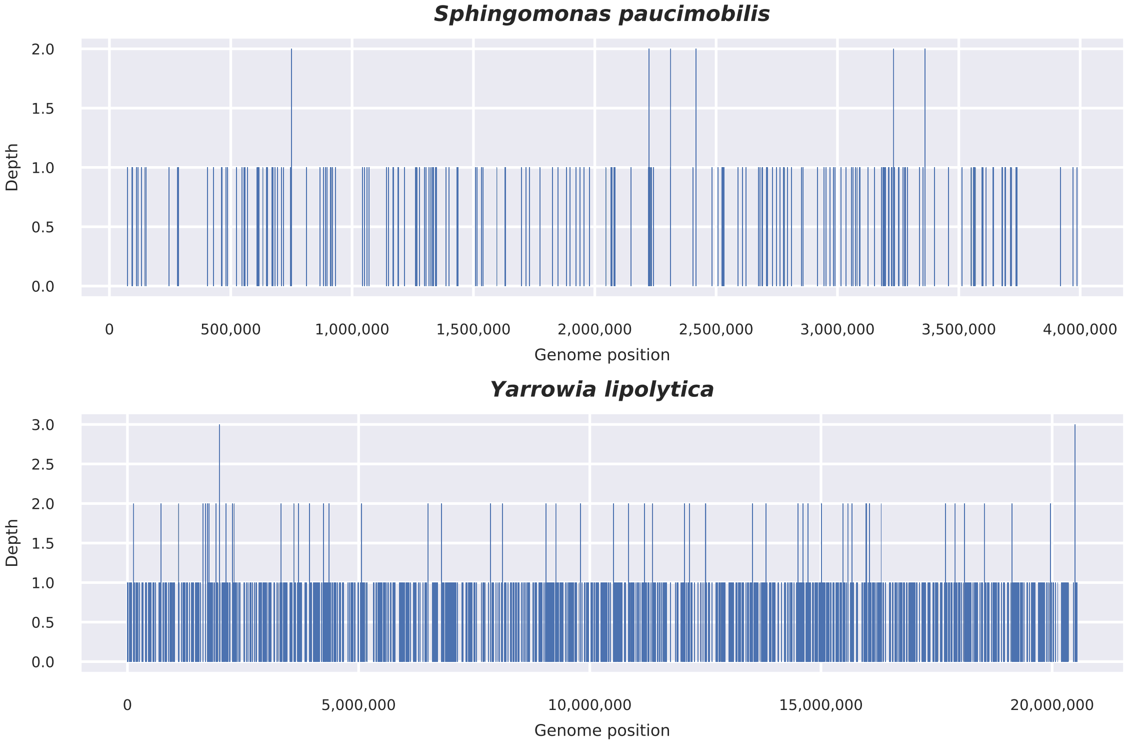

Sphingomonas paucimobilis
Depth
0.0
0.5
1.0
1.5
2.0
0
500,000
1,000,000
1,500,000
2,000,000
2,500,000
3,000,000
3,500,000
4,000,000
Genome position
Yarrowia lipolytica
Depth
0.0
0.5
1.0
1.5
2.0
2.5
3.0
0
5,000,000
10,000,000
15,000,000
20,000,000
Genome position

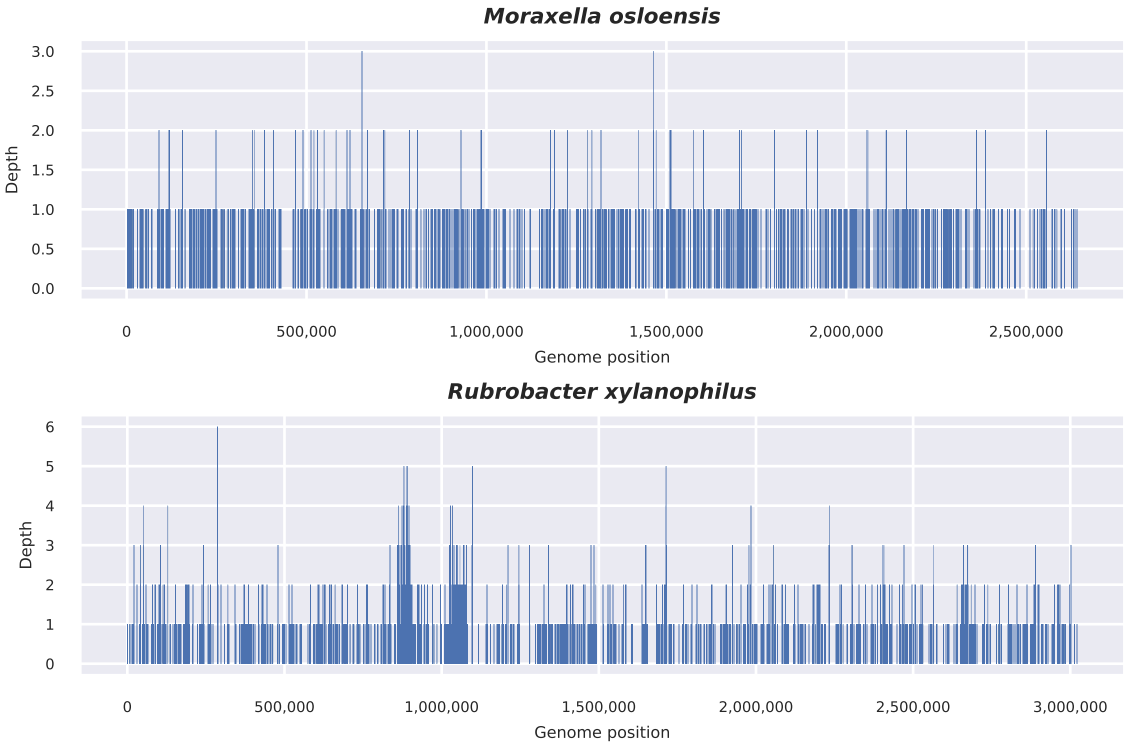

Moraxella osloensis
Depth
0.0
0.5
1.0
1.5
2.0
2.5
3.0
0
500,000
1,000,000
1,500,000
2,000,000
2,500,000
Genome position
Rubrobacter xylanophilus
Depth
0
1
2
3
4
5
6
0
500,000
1,000,000
1,500,000
2,000,000
2,500,000
3,000,000
Genome position

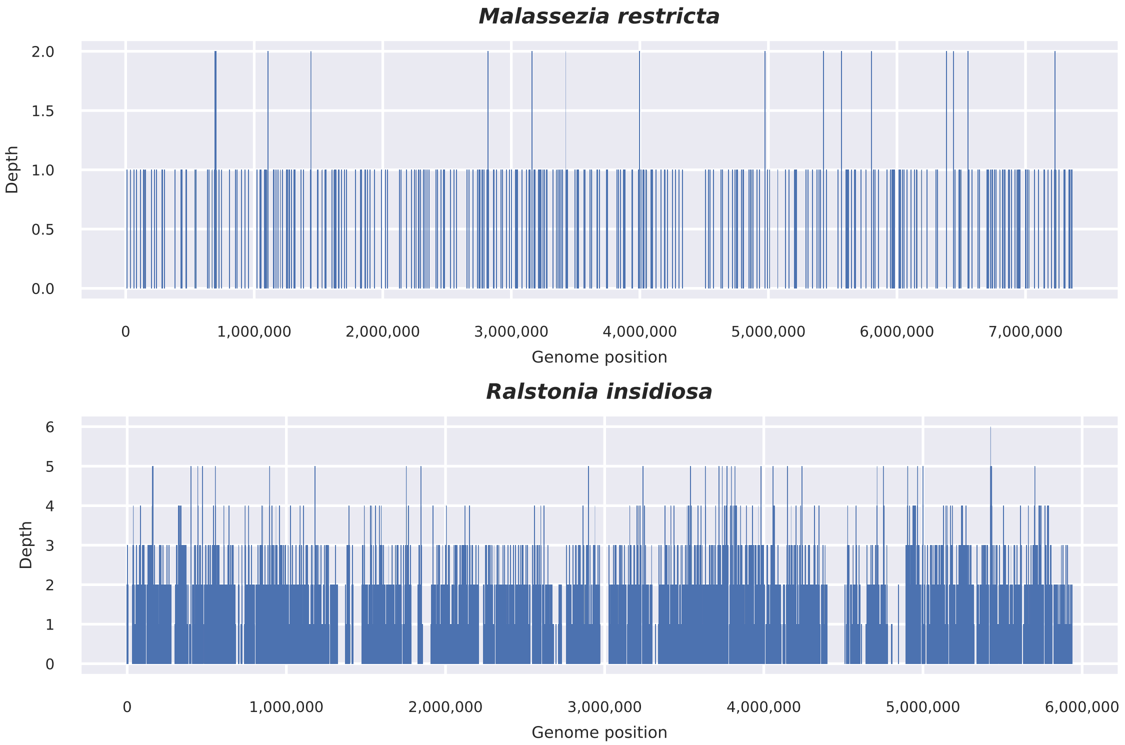
Malassezia restricta
Depth
2.0
1.5
1.0
0.5
0.0
0
1,000,000
2,000,000
3,000,000
4,000,000
5,000,000
6,000,000
7,000,000
Genome position
Ralstonia insidiosa
Depth
6
5
4
3
2
1
0
0
1,000,000
2,000,000
3,000,000
4,000,000
5,000,000
6,000,000
Genome position

Supplementary Figure 4

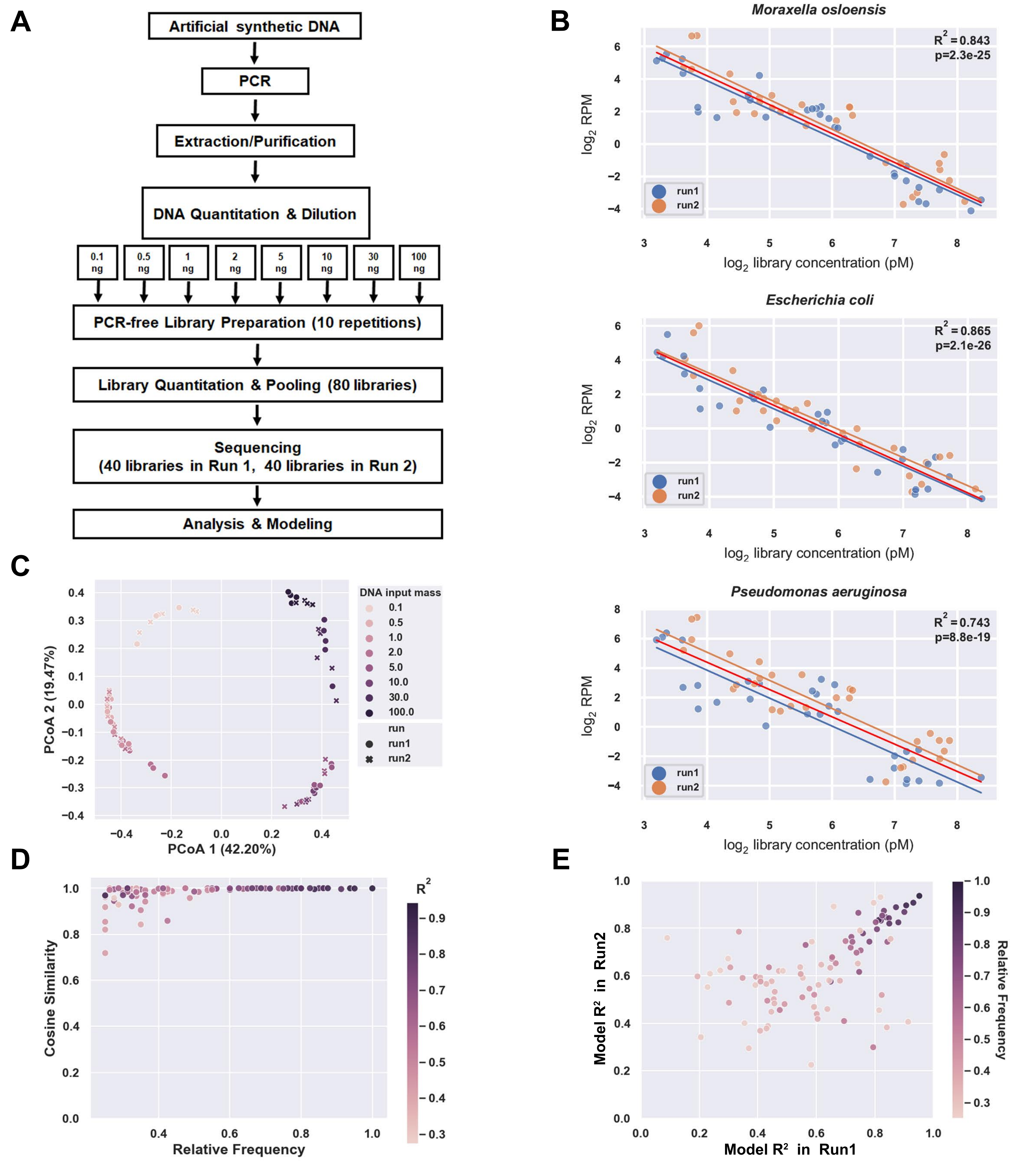

Supplemental Figure 4. BECLEAN pre-modeling analysis before the comparison with decontam, related to Figure 4

A. Flowchart of experiment design for BECLEAN modeling in Lab B. **B.** BECLEAN models of *Moraxella osloensis*, *Escherichia coli*, and *Pseudomonas aeruginosas*. Linear regression models were based on dataset of run1 (blue), run2 (tangerine) or two runs combined (red) respectively. Model R^2 and p values of the combined dataset were shown in the upper right corner of each diagram. **C.** PCoA based on Bray-Curtis distances of serial diluted artificial DNA samples sequenced in two runs. Color of data point gradually deepened as DNA input mass increased from 0.1ng to 100ng. Dots and crosses represent libraries sequenced in run1 and run2, respectively. The microbial profile varied with DNA input mass, but no significant difference in microbial profile was found between 2 sequencing runs. **D.** Similarity of background contaminant models based on two separate runs. All taxa with different relative frequency showed a high cosine similarity (close to 1.0) between two models. Each point represents a background species. X-coordinate value indicates the relative frequency of a certain background species in two runs, and y-coordinate value indicates the cosine value of the angle between two model vectors. Color bar shows the average R^2 of two models. **E.** Scatter plot of model R^2 of two sequencing runs. The model R^2 of two runs were higher and more similar if the relative frequencies of certain contaminant taxa were higher. Each point represents a background species. Color bar shows the total relative frequency of certain background species in two runs combined.

Supplementary Figure 5

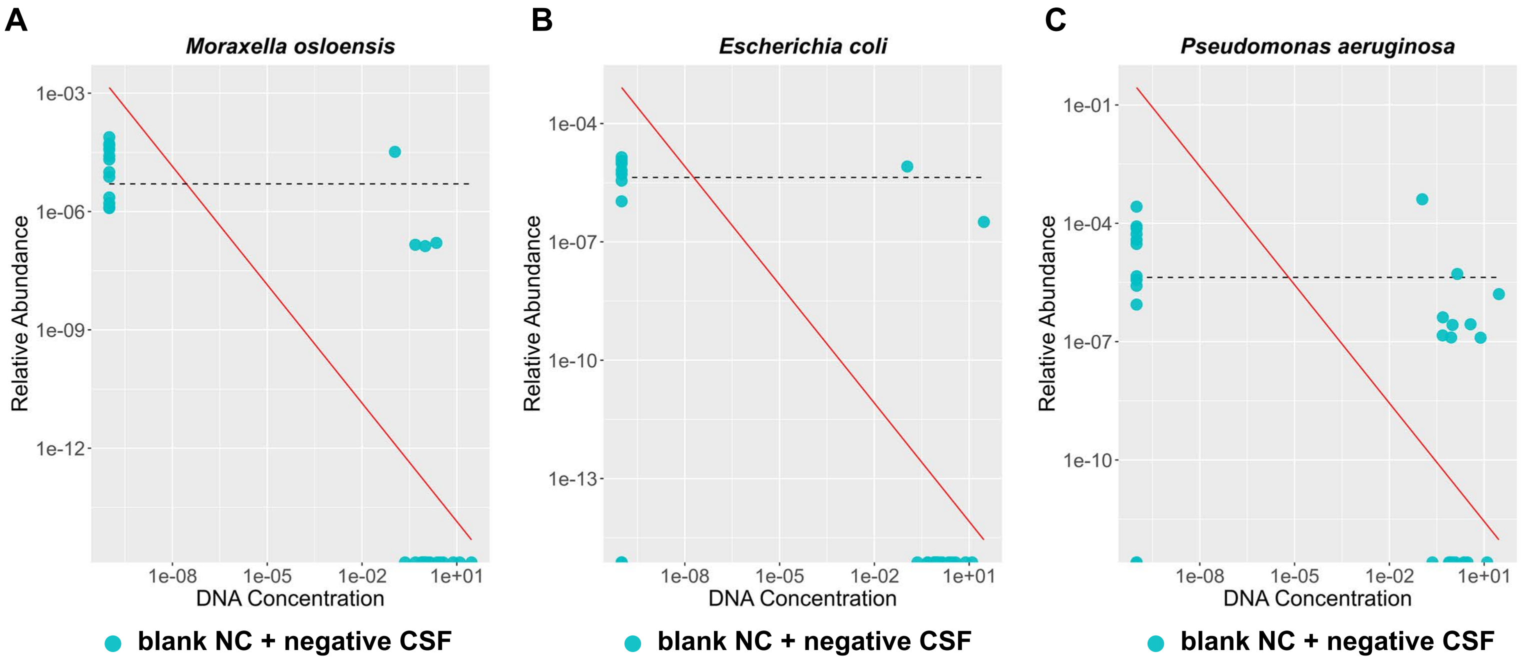

Supplemental Figure 5.
decontam modeling and analysis for *Moraxella osloensis* **(A)**, *Escherichia coli* **(B)** and *Pseudomonas aeruginosas* **(C)** using sample DNA mass. Related to Figure 4.
